# Pyranonaphthoquinones and Naphthoquinones from the Stem Bark of *Ventilago harmandiana* and Their Anti-HIV-1 Activity

**DOI:** 10.1021/acs.jnatprod.2c00980

**Published:** 2023-02-14

**Authors:** Suwannee Saisin, Kanda Panthong, Sakchai Hongthong, Chutima Kuhakarn, Sariyarach Thanasansurapong, Arthit Chairoungdua, Kanoknetr Suksen, Radeekorn Akkarawongsapat, Chanita Napaswad, Samran Prabpai, Narong Nuntasaen, Vichai Reutrakul

**Affiliations:** † Department of Chemistry and Center of Excellence for Innovation in Chemistry (PERCH-CIC), Faculty of Science, Mahidol University, Rama VI Road, Bangkok 10400, Thailand; ‡ Division of Physical Sciences and Center of Excellence for Innovation in Chemistry (PERCH-CIC), Faculty of Science, Prince of Songkla University, Songkhla 90112, Thailand; § Division of Chemistry, Faculty of Science and Technology, Rajabhat Rajanagarindra University, Chachoengsao 24000, Thailand; ∥ Department of Physiology, Faculty of Science, Mahidol University, Rama VI Road, Bangkok 10400, Thailand; ⊥ Department of Microbiology, Faculty of Science, Mahidol University, Rama VI Road, Bangkok 10400, Thailand; # The Forest Herbarium National Park, Wildlife and Plant Conservation Department, Ministry of Natural Resources and Environment, Bangkok 10900, Thailand

## Abstract

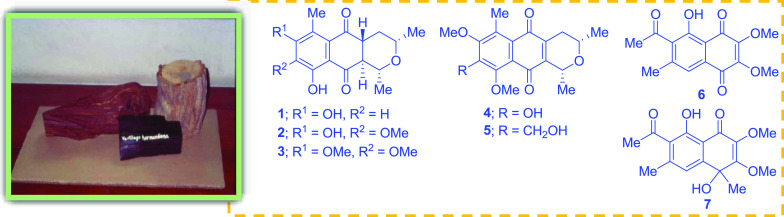

Seven previously undescribed compounds, including five pyranonaphthoquinones (ventilanones L–P) and two naphthoquinones (ventilanones Q and R), along with 15 known compounds were isolated from the stem bark of *Ventilago harmandiana* (Rhamnaceae). The structures were established by extensive analysis of their spectroscopic data. The absolute configuration of ventilanone L was established from single crystal X-ray crystallographic analysis using Cu Kα radiation and from its electronic circular dichroism data. Anti-HIV-1 activity using a syncytium inhibition assay and the cytotoxic activities of some isolated compounds were evaluated. Compounds **12**, **13**, **15**, and **16** showed activity against syncytium formation with half maximal effective concentration (EC_50_) values ranging from 9.9 to 47 μM (selectivity index (SI) 2.4–4.5).

The *Ventilago* genus belongs to the Rhamnaceae family and is found in tropical and subtropical areas. A number of plants belonging to the *Ventilago* genus play a role in traditional medicines. For example, *Ventilago africana* has been used for treatment of dysmenorrhea and as a febrifuge.^1^ The root bark of *Ventilago madraspatana* has been used as a carminative, stomachic, tonic, and stimulant. A mixture of the powdered stem bark of *Ventilago madraspatana* and sesame seed oil has been applied externally to treat skin diseases and itching.^2^ In Taiwan, the stems of *Ventilago leiocarpa* have been used as a folk medicine to cure rheumatism, hepatitis, and neuralgia.^3^ The leaves of *Ventilago denticulata* are often used as tea products. In Thai folk medicine, the stems of *V. denticulata* have been used to cure diuresis and arthritis, as well as to reduce cholesterol and sugar contents in blood.^4^

*Ventilago harmandiana* Pierre, called in Thai *Khon Tee Dum*, is a climber and is endemic to Thailand. *V. harmandiana* can be found in the limestone mountain areas located in the southern parts of Thailand. In Thai traditional medicine, the water decoction of the heartwood and stem bark of *V. harmandiana* has been used to treat diabetes, wounds, and chronic inflammation. Previous ethnopharmacological investigation of the MeOH extracts of the heartwood, stem bark, and stemwood of *V. harmandiana* revealed that they exhibited anti-inflammatory effects in both acute and chronic inflammatory assays.^5^ Recently, our research group reported the isolation of ventilanones A–K from the heartwood of *V. harmandiana*.^6^ In our continuing efforts to search for biologically active substances which exhibit cytotoxicity, anti-HIV-1 activity, and anti-inflammatory activity, the MeOH extract of the stem bark of *V. harmandiana* was investigated and led to the isolation of seven previously undescribed compounds including five pyranonaphthoquinones, ventilanones L–P (**1**–**5**), and two naphthoquinones, ventilanones Q and R (**6** and **7**), together with 15 known compounds (Supporting Information). The structures of these compounds were elucidated using the information from spectroscopic data and by comparison with those of the related analogues previously reported in the literature. The absolute configuration of ventilanone L (**1**) was established by a combination of single crystal X-ray diffraction analysis using Cu Kα radiation and its electronic circular dichroism (ECD) data. The structures and absolute configuration of ventilanones M–P (**2**–**5**) and ventilanone R (**7**) were established by comparison of their spectroscopic data and ECD patterns with those of ventilanone L (**1**) and related known compounds. The biological activities of some of the isolated compounds were evaluated in a cytotoxicity assay against a panel of cultured mammalian cancer cell lines, and anti-HIV-1 activity was evaluated using a syncytium inhibition assay. Most of the compounds screened for their cytotoxic activity were found inactive (half maximal inhibitory concentration (IC_50_) > 20 μM). In the anti-HIV-1 activity, some of the tested compounds exhibited moderate to good inhibitory activity against syncytium formation with half maximal effective concentration (EC_50_) values in the range 9.9–47 μM (selectivity index (SI) 2.4–4.5). A structure–activity relationship of the pyranonaphthoquinones toward their anti-HIV-1 activity against syncytium formation is also discussed in this article.

## Results and Discussion

**Chart 1:**
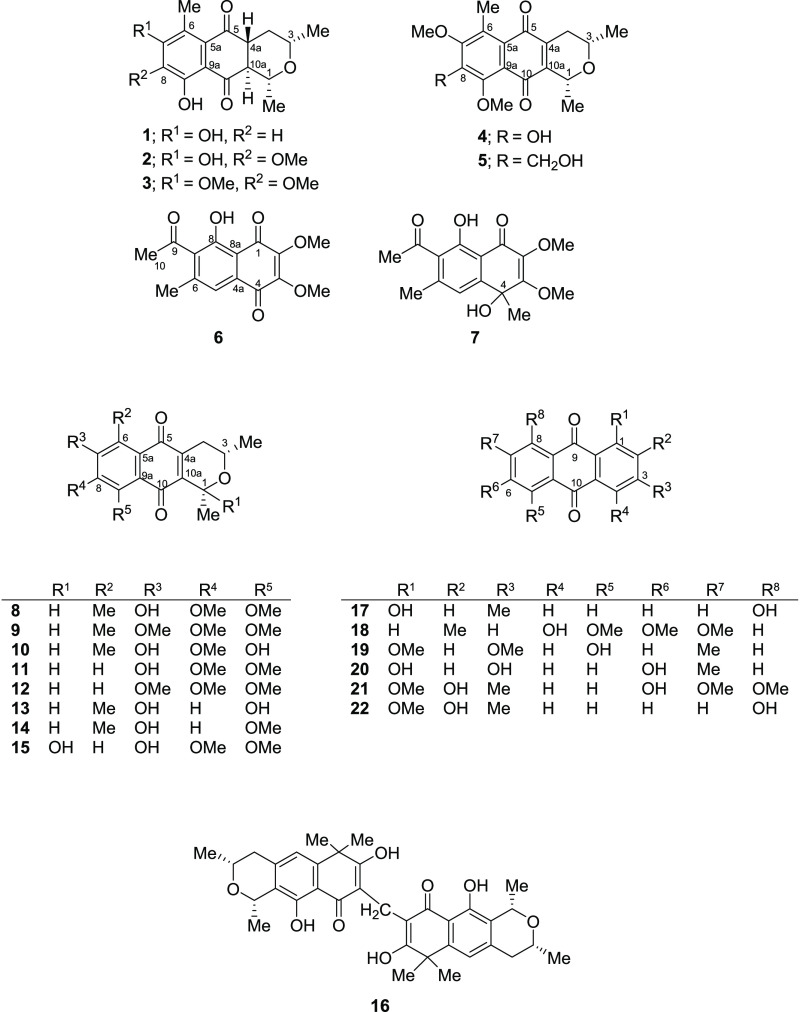


Phytochemical investigation of the MeOH extract obtained from the stem bark of *V. harmandiana* led to the isolation of seven previously undescribed compounds, including five pyranonaphthoquinones, ventilanones L–P (**1**–**5**), and two naphthoquinones, ventilanones Q and R (**6** and **7**). Fifteen known compounds were identified as ventilanones A–E (**8**–**12**) and G–I (**13**–**15**);^6^ a dimer of pyranonaphthoquinone, 8,8′-methylenebis(7,10-dihydroxy-1,3,6,6-tetramethyl-3,4-dihydro-1*H*-benzo[*g*]isochromen-9-one) (**16**);^7^ chrysophanol (**17**);^8^ 4-hydroxy-5,6,7-trimethoxy-2-methylanthraquinone (**18**);^9^ 5-hydroxy-1,3-dimethoxy-7-methylanthraquinone (**19**);^10^ demethylmacrosporine I (**20**);^11^ 2,6-dihydroxy-1,7,8-trimethoxy-3-methylanthraquinone (**21**);^12^ and obtusifolin (**22**)^13^ (Chart 1). It is worth emphasizing that the spectroscopic data of compounds **16** and **21** have never been reported. The present work describes for the first time complete spectroscopic data of both compounds (see the Supporting Information).

All of the new pyranonaphthoquinones reported in this work exhibited characteristic UV and FTIR patterns similar to those of known pyranonaphthoquinones as follows: (1) approximate major UV absorption bands at 350, 288, and 256 nm;^14,15^ (2) approximate FTIR absorption bands at ν_max_ 1700–1650 cm^–1^ (conjugated C=O stretching), 1550–1400 cm^–1^ (C=C stretching), and 1200–1100 cm^–1^ (C–O stretching).^16,17^

**Figure 1:**
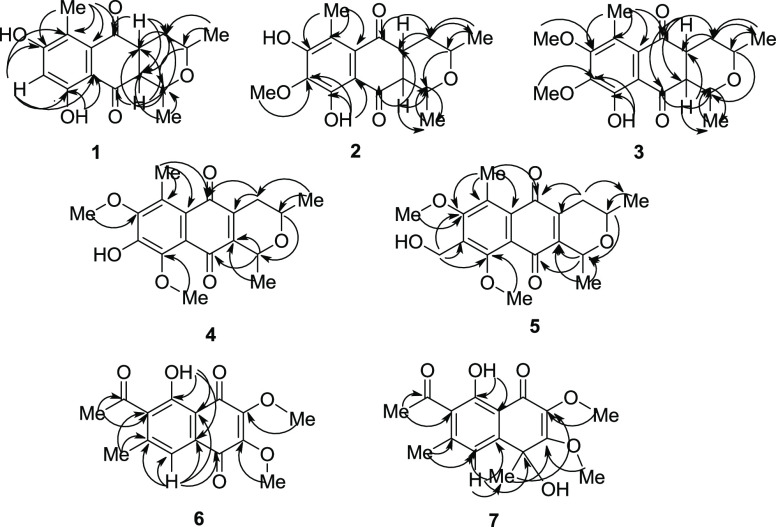
Key HMBC correlations for compounds **1**–**7**.

**Figure 2:**
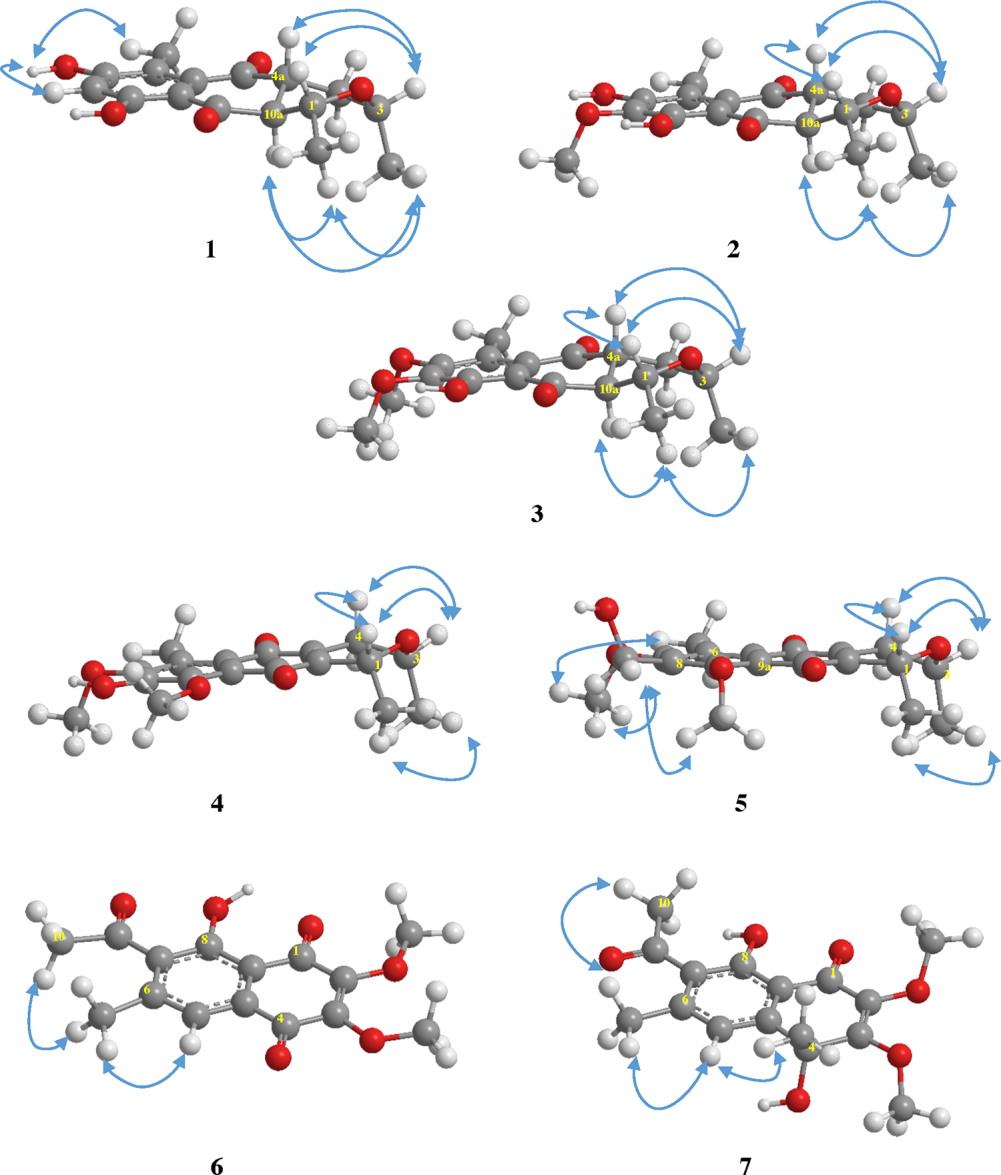
Key NOESY correlations for compounds **1**–**7**. The 3D structures were obtained by MM2 optimization using Chem 3D software.

**Figure 3:**
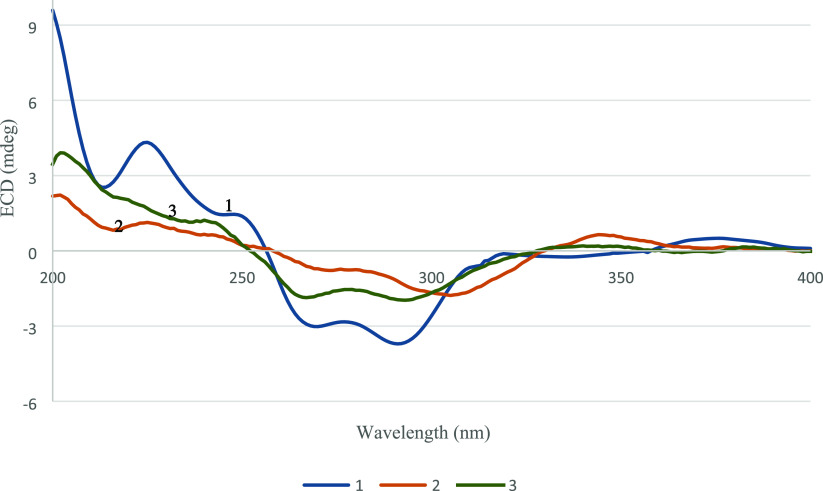
ECD spectra (MeOH) of **1**–**3**.

**Figure 4:**
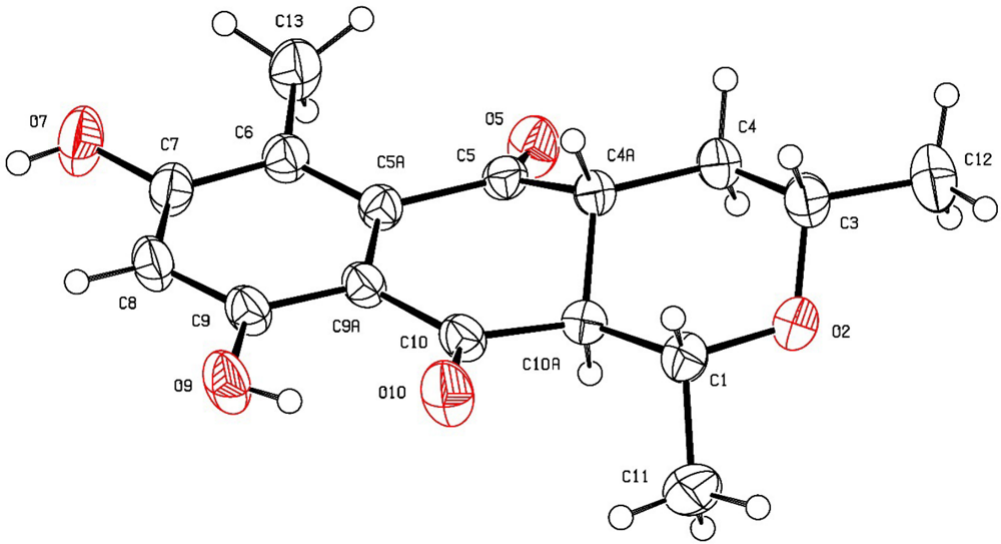
ORTEP diagram of **1**. Displacement ellipsoids are drawn at 40% probability.

Compound **1** was isolated as orange rods with mp 254–255 °C (crystallized from CH_2_Cl_2_–hexane). Its molecular formula, C_16_H_18_O_5_, was established by high-resolution electrospray ionization mass spectrometry (HRESIMS), which showed a sodium adduct ion peak at *m*/*z* 313.1056 [M + Na]^+^. The ^1^H NMR data (Table 1) of **1** showed resonances at δ_H_ 3.85 (dq, *J* = 9.1, 5.9 Hz, H-1), 3.51 (ddq, *J* = 11.2, 1.8, 6.1 Hz, H-3), 1.96 (ddd, *J* = 13.8, 4.0, 1.8 Hz, Ha-4), 1.68 (m, Hb-4), 1.58 (d, *J* = 5.9 Hz, CH_3_-1), and 1.28 (d, *J* = 6.1 Hz, CH_3_-3). According to the ^1^H–^13^C correlations in the heteronuclear multiple quantum coherence (HMQC) spectrum, these resonances connected to the carbons at δ_C_ 72.7 (C-1), 71.5 (C-3), 32.5 (C-4), 21.9 (CH_3_-1), and 21.8 (CH_3_-3), respectively. The locations of an aromatic proton at δ_H_ 6.57 (s), a methyl group at δ_H_ 2.42 (s), and a proton at δ_H_ 12.52 (s) with intramolecular hydrogen bonding were assigned to be H-8, CH_3_-3, and OH-9, respectively, based on the heteronuclear multiple bond coherence (HMBC) correlations (H-8/C-6, C-7, C-9a; CH_3_-6/C-5, C-5a, C-6, C-7; OH-9/C-9, C-9a (Figure 1). The ^1^H and ^13^C NMR data of compound **1** were similar to those of ventilanone G (**13**).^6^ The key difference between the two compounds was that compound **1** possessed additional resonances of two methine protons at δ_H_ 2.55 (dd, *J* = 13.4, 9.1 Hz) and 3.09 (ddd, *J* = 13.4, 11.5, 4.0 Hz). By comparison with the previously reported ^1^H NMR data of dihydrofusarubin, these signals were assigned as methine protons at C-10a and C-4a, respectively.^18^ These assignments were supported by the correlations of H-10a/C-1, C-4, C-4a, and C-5 as well as H-4a/C-10a, C-4, and C-5 in the HMBC experiments (Figure 1). The magnitude of the vicinal coupling constant observed between H-4a and H-10a (^3^*J* = 13.4 Hz) established a *trans*-diaxial relationship similar to those of dihydrofusarubin.^18−20^ The relative configuration of compound **1** was also confirmed by NOESY experiments and led to the assignment of the relative configuration of compound **1** as shown in Figure 2. The NOESY correlations of H-1 and H-4a with H-3 suggested that they were oriented on the same face of the preferred chairlike conformation (Figure 2). In addition, the NOESY correlations of H_3_-1/H-10a, H_3_-3/H-10a, and H_3_-1/H_3_-3 further supported that H-10a, H_3_-1, and H_3_-3 were located on the same face. Finally, the chemical structure and the absolute configuration of compound **1** were also concluded by electronic circular dichroism (ECD) data (Figure 3) and single crystal X-ray crystallographic analysis using Cu Kα radiation (Figure 4). Compound **1** formed monoclinic crystals with space group *P*2_1_ (No. 4), *a* = 4.8054(11) Å, *b* = 7.5474(6) Å, *c* = 19.632(3) Å, β = 94.39(2)°, *V* = 709.9(2) Å^3^, and *Z* = 2. The crystal data were collected at *T* = 273(2) K with Flack parameter = 0.12(8). The ECD spectrum indicated a negative Cotton effect (CE) at 337, 292, and 270 nm and a positive CE at 249, and 226 nm suggesting the π–π* and n−π* transitions of pyranonaphthoquinones.^21−24^ On the basis of the analysis data described for compound **1**, the absolute configuration of **1** was assigned as 1*R*, 3*S*, 4a*R*, and 10a*S*, and thus compound **1** was identified as (1*R*,3*S*,4a*R*,10a*S*)-7,9-dihydroxy-1,3,6-trimethyl-3,4,4a,10a-tetrahydro-1*H*-naphtho[2,3-*c*]pyran-5,10-dione and was named ventilanone L.

**Table 1:** ^1^H (400 MHz) NMR Spectroscopic Data of Compounds **1**–**5** in CDCl_3_

	δ_H_, mult (*J* in Hz)				
position	**1**	**2**	**3**	**4**	**5**
1	3.85, dq (9.1, 5.9)	3.84, dq (9.1, 5.9)	3.86, dq (9.1, 5.9)	4.80, ddq (6.6, 3.8, 2.6)	4.80, ddq (6.6, 3.8, 2.6)
3	3.51, ddq (11.2, 1.8, 6.1)	3.50, ddq (11.6, 1.7, 6.2)	3.51, ddq (11.4, 1.6, 6.1)	3.55, ddq (10.3, 6.0, 2.6)	3.55, ddq (10.3, 6.0, 2.6)
4	(a) 1.96, ddd (13.8, 4.0, 1.8)	(a) 1.97, ddd (13.8, 4.0, 1.6)	(a) 1.99, ddd (13.7, 4.0, 1.6)	(a) 2.1, ddd (18.4, 10.3, 3.8)	(a) 2.1, ddd (18.4, 10.3, 3.8)
	(b) 1.68, m	(b) 1.63, dt (13.8, 11.6)	(b) 1.64, dt (13.6, 11.4)	(b) 2.75, dt (18.4, 2.6)	(b) 2.85, dt (18.4, 2.6)
8	6.57, s	–	–	–	–
4a	3.09, ddd (13.4, 11.5, 4.0)	3.04, ddd (13.5, 11.6, 4.0)	3.04, ddd (13.5, 11.4, 4.0)	–	–
10a	2.55, dd (13.4, 9.1)	2.56, dd (13.5, 9.1)	2.58, dd (13.5, 9.1)	–	–
1-CH_3_	1.58, d (5.9)	1.57, d (5.9)	1.57, d (5.9)	1.50, d (6.6)	1.50, d (6.6)
3-CH_3_	1.28, d (6.1)	1.27, d (6.2)	1.28, d (6.1)	1.35, d (6.1)	1.35, d (6.0)
6-CH_3_	2.42, s	2.44, s	2.45, s	2.50, s	2.55, s
7-OH	6.17, br s	6.75, br s	–	–	–
8-OH	–	–	–	6.43, br s	–
9-OH	12.52, s	12.71, s	12.52, s	–	–
7-OCH_3_	–	–	3.95, s	3.90, s	3.85, s
8-OCH_3_	–	4.06, s	3.98, s	–	–
9-OCH_3_	–	–		3.93, s	3.95, s
8-CH_2_	–	–		–	4.80, s

Compound **2** was obtained as yellow rods with mp 174–175 °C (crystallized from CH_2_Cl_2_–hexane). Its molecular formula, C_17_H_20_O_6_, was determined by HRESIMS, which showed a sodium adduct ion peak at *m*/*z* 343.1157 [M + Na]^+^. ^1^H and ^13^C NMR data of **2** (Tables 1 and 2, respectively) were similar to those of **1** except for the replacement of an aromatic proton at δ_H_ 6.57 (s, H-8) in **1** by a methoxy group (δ_H_ 4.06 (s) and δ_C_ 60.9). This assignment was supported by HMBC data (Figure 1) of H_3_-8 to C-8. In addition, OH-9 also showed HMBC correlations to oxygenated aromatic carbons C-8 and C-9. Complete assignments of ^1^H (Table 1) and ^13^C (Table 2) NMR data for each proton and carbon were established using HMBC information (Figure 1). The correlations of H_3_-1/H-10a, H_3_-1/H_3_-3, H-1/H-3, H-1/H-4a, and H-3/H-4a in the NOESY spectrum (Figure 2) showed that compound **2** shares the same relative configuration as **1** with the *trans*-diaxial orientation of H-4a and H-10a as well as the cofacial relationships between H-10a, CH_3_-1, and CH_3_-3. The absolute configuration of **2** was confirmed by a comparison of the experimental ECD data of **2** with those of **1** (Figure 3). Therefore, compound **2** was identified as (1*R*,3*S*,4a*R*,10a*S*)-7,9-dihydroxy-8-methoxy-1,3,6-trimethyl-3,4,4a,10a-tetrahydro-1*H*-naphtho[2,3-*c*]pyran-5,10-dione and was named ventilanone M.

**Table 2:** ^13^C (100 MHz) NMR Spectroscopic Data of Compounds **1**–**5** in CDCl_3_

	δ_C_, type				
position	**1**	**2**	**3**	**4**	**5**
1	72.7, CH	72.5, CH	72.5, CH	69.6, CH	69.7, CH
3	71.5, CH	71.5, CH	71.5, CH	68.9, CH	69.0, CH
4	32.5, CH_2_	32.5, CH_2_	32.6, CH_2_	29.8, CH_2_	29.6, CH_2_
5	201.4, C=O	200.4, C=O	202.8, C=O	184.4, C=O	185.5, C=O
6	118.8, C	119.4, C	126.4, C	123.6, C	131.3, C
7	161.7, C	153.9, C	157.8, C	149.7, C	162.6, C
8	107.3, CH	136.9, C	144.8, C	147.3, C	134.2, C
9	162.3, C	153.1, C	155.0, C	146.1, C	158.2, C
10	198.5, C=O	197.5, C=O	197.3, C=O	184.0, C=O	183.8, C=O
4a	50.6, CH	50.3, CH	50.1, CH	141.9, C	141.4, C
5a	134.7, C	129.0, C	128.5, C	132.5, C	132.5, C
9a	112.9, C	113.2, C	115.4, C	122.3, C	122.7, C
10a	53.3, CH	53.4, CH	53.6, CH	146.0, C	147.2, C
1-CH_3_	21.9, CH_3_	21.8, CH_3_	21.7, CH_3_	20.2, CH_3_	20.2, CH_3_
3-CH_3_	21.8, CH_3_	21.9, CH_3_	21.9, CH_3_	21.1, CH_3_	21.2, CH_3_
6-CH_3_	11.6, CH_3_	12.1, CH_3_	12.7, CH_3_	13.8, CH_3_	14.0, CH_3_
7-OCH_3_	–	–	61.0, CH_3_	60.0, CH_3_	62.1, CH_3_
8-OCH_3_	–	60.9, CH_3_	60.8, CH_3_	–	–
9-OCH_3_	–	–	–	62.0, CH_3_	63.0, CH_3_
6-CO	–	–	–	–	–
8-CH_2_	–	–	–	–	55.5, CH_2_

Compound **3** was obtained as pale yellow rods with mp 116–117 °C (crystallized from CH_2_Cl_2_–hexane). Its molecular formula, C_18_H_22_O_6_, was determined by HRESIMS, which showed a sodium adduct ion peak at *m*/*z* 357.1309 [M + Na]^+^. A comparison of its ^1^H and ^13^C NMR experimental data (Tables 1 and 2, respectively) with those of compound **2** suggested that these compounds have related structures with the only difference being the resonance of a phenol proton at δ_H_ 6.75 (s) at C-7 was replaced by the resonance of methoxy protons at δ_H_ 3.95 (s, 3H). This assumption was supported by the HMBC correlation between H_3_-7 (δ_H_ 3.95) and C-7 (δ_C_ 157.8). On the basis of the NOESY correlations, compound **3** shares the same relative configurations as those of **1** and **2**. The absolute configuration of **3** was confirmed by comparison of its experimental ECD data with those of **1** and **2** (Figure 3). From the aforementioned data, compound **3** was identified as (1*R*,3*S*,4a*R*,10a*S*)-7,8-dimethoxy-9-hydroxy-1,3,6-trimethyl-3,4,4a,10a-tetrahydro-1*H*-naphtho[2,3-*c*]pyran-5,10-dione and was named ventilanone N.

**Figure 5:**
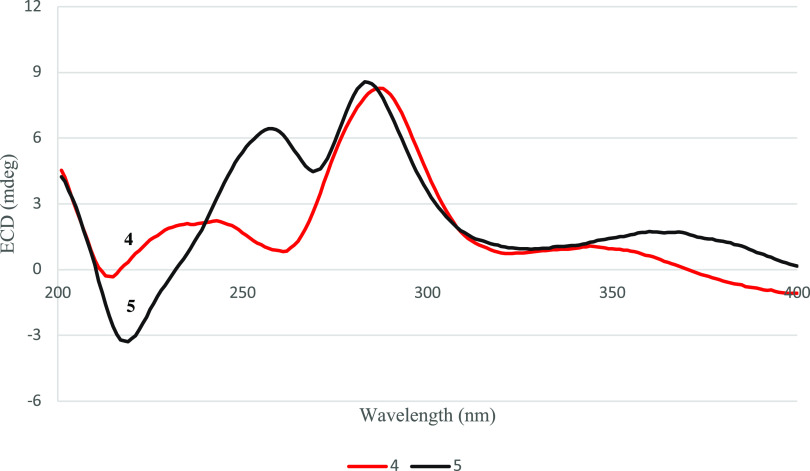
Overlay of ECD curves of **4** and **5**.

Compound **4** was obtained as yellow needles with mp 136–137 °C (crystallized from CH_2_Cl_2_–hexane). Its molecular formula, C_18_H_20_O_6,_ was deduced from HRESIMS data which displayed a sodium adduct ion peak at *m*/*z* 355.1163 [M + Na]^+^. The ^1^H and ^13^C NMR spectroscopic data of **4** (Tables 1 and 2, respectively) closely resembled those of ventilanone A (**8**).^6^ The key difference was the shift of a methoxy resonance at δ_H_ 4.03 (OCH_3_-8) in compound **8** to a new methoxy group at δ_H_ 3.90 (OCH_3_-7)/δ_C_ 60.0 (OCH_3_-7) in compound **4**. The HMBC data shown in Figure 1 supported the assignment of the location of the methoxy group at C-7 [δ_H_ 3.90 (H_3_-7) to δ_C_ 149.7 (C-7)]. The complete assignment of each ^1^H and ^13^C signal (see Tables 1 and 2, respectively) was deduced by 2D NMR data (HMQC and HMBC). The NOESY correlations between H-1 and H-3 as well as between H_3_-1 and H_3_-3 (Figure 2) suggested the *cis*-relationship of CH_3_-1 and CH_3_-3. Therefore, the relative configuration of compound **4** was concluded to be as shown. The ECD spectrum of compound **4** (Figure 5) was similar to that of compound **8**, whose absolute configuration was previously established by means of X-ray crystallographic analysis of its *p*-bromobenzenesulfonate derivative, with the ECD data suggesting the (1*R*,3*S*) absolute configuration.^7^ On the basis of the spectroscopic data of **4** above and the comparison of its spectroscopic data with those of **8**, compound **4** was assigned as (1*R*,3*S*)-7,9-dimethoxy-8-hydroxy-1,3,6-trimethyl-3,4-dihydro-1*H*-naphtho[2,3-*c*]pyran-5,10-dione and was named ventilanone O.

Compound **5** was isolated as yellow needles with mp 126–127 °C (crystallized from CH_2_Cl_2_–hexane). Its molecular formula, C_19_H_22_O_6_, was determined by HRESIMS data which displayed a sodium adduct peak at *m*/*z* 369.1313 [M + Na]^+^. The ^1^H and ^13^C NMR spectroscopic data of **5** (Tables 1 and 2, respectively) were similar to those of **4**, except that the OH-8 group was replaced by a hydroxymethylene (CH_2_OH) (δ_H_ 4.80 (s, 2H)/δ_C_ 55.5). The hydroxymethylene was located at C-8 according to the HMBC correlations of δ_H_ 4.80 with C-7 (δ_C_ 162.6), C-8 (δ_C_ 134.2), and C-9 (δ_C_ 158.2) (Figure 1). This assignment was also confirmed by the NOESY correlations of H_2_-8 to H_3_-7 and H_3_-9 (Figure 2). The (1*R*,3*S*) absolute configuration of **5** was concluded by comparison of its ECD data with those of **4** (Figure 5). Therefore, compound **5** was identified as (1*R*,3*S*)-7,9-dimethoxy-8-hydroxymethyl-1,3,6-trimethyl-3,4-dihydro-1*H*-naphtho[2,3-*c*]pyran-5,10-dione and was named ventilanone P.

The 4a,10a-dihydropyranonaphthoquinones are a unique class of compounds and are commonly found in fungi and related microorganisms.^25−27^ Some of the 4a,10a-dihydropyranonaphthoquinones possessed potent biological activities, e.g., cytotoxicity and anti-HIV-1 and antibacterial activities.^28−31^ Dihydrofusarubin, a 4a,10a-dihydropyranonaphthoquinone derivative, is a secondary metabolite obtained from various species of *Fusarium* and exhibited antifungal activity.^20^ From our exhaustive search (SciFinder Scholar database), this work is the first report on the isolation of 4a,10a-dihydropyranonaphthoquinone derivatives from plants in the *Ventilago* genus.

Compound **6** was obtained as orange rods with mp 146–147 °C (crystallized from CH_2_Cl_2_–hexane). Its molecular formula, C_15_H_14_O_6_, was established from HRESIMS data which displayed a sodium adduct ion peak at *m*/*z* 313.0691 [M + Na]^+^. ^1^H and ^13^C NMR spectroscopic data of **6** (Table 3) indicated the presence of an aromatic proton at δ_H_ 7.45 (s, H-5), two methyl groups at δ_H_ 2.30 (s, CH_3_-6) and 2.56 (s, CH_3_-10), and two methoxy groups at δ_H_ 4.10 (s, OCH_3_-3) and 4.13 (s, OCH_3_-2). According to the ^1^H–^13^C correlations in the HMQC data, these resonances connected to the respective carbons at δ_C_ 121.4 (C-5), 20.1 (CH_3_-6), 31.9 (CH_3_-10), 61.7 (OCH_3_-3), and 61.5 (OCH_3_-2). A hydrogen-bonded hydroxy resonance [δ_H_ 12.20 (s)] was located at C-8 according to the HMBC cross-peak with δ_C_ 158.2 (C-8) (Figure 1). The ^1^H and ^13^C NMR data of **6** were similar to those of 2-methoxystypandrone except for the replacement of an olefinic proton (δ_H_ 6.10, s, H-2) in 2-methoxystypandrone by a methoxy (δ_H_ 4.13, s, H_3_-2) at C-2.^32,33^ This assignment was supported by an HMBC correlation of δ_H_ 4.13 to δ_C_ 148.1 (C-2) (Figure 1). Complete assignments of ^1^H and ^13^C NMR data of **6** as shown in Table 3 were deduced by HMBC data (Figure 1). Additionally, the NOESY experiment (Figure 2) that showed correlations of H-5/H_3_-6 and H_3_-6/H_3_-10 led to the assignment of the location of a methyl group at C-6 and an acetyl group at C-7. On the basis of the aforementioned data and by comparison of the spectroscopic data of **6** with those of the previously reported compounds, compound **6** was identified as 7-acetyl-2,3-dimethoxy-8-hydroxy-6-methylnaphthalene-1,4-dione and was named ventilanone O.

**Table 3:** ^1^H (400 MHz) and ^13^C (100 MHz) NMR Spectroscopic Data of Compounds **6** and **7** in CDCl_3_

	**6**		**7**	
position	δ_C_, type	δ_H_, mult (*J* in Hz)	δ_C_, type	δ_H_, mult (*J* in Hz)
1	186.8, C=O	–	187.0, C=O	–
2	148.1, C	–	133.6, C	–
3	146.7, C	–	163.3, C	–
4	180.9, C=O	–	71.7, C=O	–
5	121.4, CH	7.45, s	118.6, CH	7.05, s
6	144.2, C	–	143.4, C	–
7	136.0, C	–	129.5, C	–
8	158.2, C	–	158.8, C	–
9	202.7, C=O	–	203.9, C=O	–
10	31.9, CH_3_	2.56, s	32.1, CH_3_	2.58, s
4a	130.4, C	–	143.4, C	–
8a	111.5, C	–	110.7, C	–
4-CH_3_	–	–	32.6, CH_3_	1.66, s
6-CH_3_	20.1, CH_3_	2.34, s	20.4, CH_3_	2.33, s
2-OCH_3_	61.5, CH_3_	4.13, s	61.3, CH_3_	3.81, s
3-OCH_3_	61.7, CH_3_	4.10, s	61.4, CH_3_	4.30, s
8-OH	–	12.2, s	–	12.93, s

Compound **7** was obtained as pale yellow needles with mp 139–140 °C (crystallized from CH_2_Cl_2_–hexane). Its molecular formula was established as C_16_H_18_O_6_ from HRESIMS data which displayed a sodium adduct peak at *m*/*z* 329.1002 [M + Na]^+^]. The NMR spectroscopic data of **7** are similar to those of **6** (Table 3). The differences included the disappearance of a carbonyl resonance at δ_C_ 180.9 (C-4) in **6** but the existence of an additional methyl group at δ_H_ 1.66 (s, CH_3_-4)/δ_C_ 32.6 (CH_3_-4) found in **7**. This methyl group was located at C-4 according to its HMBC correlations with an aromatic proton at δ_H_ 7.05 (H-5) and an oxygenated nonhydroxygenated carbon at δ_C_ 71.7 (C-4). This assignment was also confirmed by the NOESY correlations of H_3_-4 to the nearby aromatic proton, H-5 (Figure 2). The lack of an optical rotation and the flat ECD spectrum indicated that **7** was a racemic mixture. Subsequently, the enantiomers of **7** were resolved approximately in a ratio of 1:1 by HPLC using a Chiralpak OD-H column. The determination of absolute configurations was performed by comparison of specific rotation and ECD data with those of the previously reported derivatives (Supporting Information).^34−36^ Thus, compound **7** was determined to be 7-acetyl-4,5-dihydroxy-2,3-dimethoxy-4,7-dimethylnaphthalen-1-(1*H*)-one and was named ventilanone R.

Some isolated pyranonaphthoquinone and naphthoquinone derivatives have been evaluated for their cytotoxic effects and anti-HIV-1 activities. In this study, the anti-HIV-1 activity was studied using a syncytium inhibitory assay (Table 4). In the present work, in order to focus on the active compounds, the EC_50_ maximum cutoff was set at 50 μM. Compounds **12**, **13**, and **15** along with a dimeric **16** showed moderate to good inhibitory activities in the syncytium reduction assay with EC_50_ values in the range 9.9–47 μM (SI 2.4–4.5) while 4a,10a-dihydropyranonaphthoquinone derivatives are inactive (EC_50_ > 50 μM). Compound **15** was the most active in the syncytium reduction assay with an EC_50_ value of 9.9 μM (SI 4.5). Almost all the compounds screened for cytotoxicity against a panel of cultured mammalian cancer cell lines did not show cytotoxicity (IC_50_ > 20 μM) (see Table S1).

**Table 4:** Anti-HIV-1 in the Syncytium Reduction Assay of Compounds **1**–**16**

	syncytium (^ΔTat/Rev^MC99 + 1A2) assay^a^			
compound	IC_50_ (μM)	EC_50_ (μM)	SI (IC_50_/EC_50_)	activity
**1**	130	120	1.1	A
**2**	300	110	2.7	A
**3**	>370	–	–	I
**4**	130	170	0.76	I
**5**	200	110	1.8	A
**6**	<13	–	–	T
**7**	410	160	2.6	A
**8**	190	160	1.2	A
**9**	290	100	2.9	A
**10**	130	140	0.93	I
**11**	280	280	1.0	I
**12**	98	41	2.4	A
**13**	98	36	2.7	A
**14**	250	200	1.3	A
**15**	44	9.9	4.5	A
**16**	210	47	4.5	A

a Cytotoxic assay: IC_50_ = dose of compound that inhibited 50% metabolic activity of uninfected 1A2 cells. AZT, averaged from three experiments, IC_50_ > 10^–2^ μM (less than 50% inhibition at this concentration). Syncytium assay: EC_50_ = dose of compound that reduced 50% syncytium formation by ^ΔTat/Rev^MC99 virus in 1A2 cells. Positive control, AZT, averaged from three experiments, EC_50_ = 4.6 × 10^–3^ μM, SD = 5.7 × 10^–4^ μM, SI > 2.1. SI, selectivity index: IC_50_/EC_50_. Activity: A, active (SI > 1); I, inactive (SI < 1); T, toxic (IC_50_ is less than the lowest concentration tested).

Based on the preliminary screening results on anti-HIV-1 activities of pyranonaphthoquinones, the structure–activity relationship can be discussed. The presence of the unsaturation bond between C-4a and C-10a in the structure of a pyranonaphthoquinone is important for the anti-HIV-1 activity in the syncytium reduction assay, while the position and number of oxygenated groups on the naphthoquinone moiety at C-7, C-8, and C-9 had no effect. Additionally, the hydroxy group at C-1 of the pyran ring found in **15** had a significant effect and enhanced inhibitory activity against syncytium formation.

## Experimental Section

### General Experimental Procedures

Melting points (uncorrected) were measured on an Electrothermal 9100 melting point apparatus. Optical rotations were measured on a JASCO DIP-370 digital polarimeter by using a 50 mm microcell (1 mL). The UV spectra were recorded on a JASCO V-530 spectrometer. The ECD spectra were measured on a JASCO J-815 spectropolarimeter by using 10 and 50 mm microcells. The IR spectra were recorded on a PerkinElmer System 2000 FTIR spectrophotometer. The NMR spectra were recorded on a Bruker DPX 300, Bruker Ascend 400, or Jeol NMR 400 MHz spectrometer with tetramethylsilane (TMS) as an internal reference. HRESIMS spectra were measured on a Bruker micro TOF spectrometer. EIMS spectra were measured on a Thermo Finnigan Polaris Q mass spectrometer at 70 eV (probe). Analytical TLC was carried out on a TLC aluminum sheet (Silica gel PF_254_, 20 × 20 cm, layer thickness 0.2 mm, Merck), or preparative TLC was prepared on glass plates coated with silica gel (silica gel PF_254_, Merck). Column chromatography (CC) was performed on silica gel 60 (70–230 mesh ASTM, Merck) eluted with gradient solvent systems [system A (hexane–acetone, 100:0 to 0:100 v/v; acetone–MeOH, 100:0 to 0:100 v/v) and system B (hexane–CH_2_Cl_2_, 100:0 to 0:100 v/v; CH_2_Cl_2_–MeOH, 100:0 to 0:100 v/v)]. Gel filtration was performed using Sephadex LH-20. Solvents for extraction and chromatography were distilled at their boiling point ranges prior to use. Analytical grade solvents were used for crystallization.

### Plant Material

The stem bark of *Ventilago harmandiana* was collected in June 1996 from Pang-Nga Province, Thailand (lat. 7° 47′ 12.8″ N, long. 99° 30′ 55.0″ E; altitude 104 m above sea level). A voucher specimen (BKF no. 35203) was deposited at the Forest Herbarium, Department of National Parks, Wildlife and Plant Conservation, Bangkok, Thailand.

### Extraction and Isolation

The air-dried stem bark of *V. harmandiana* (9.7 kg) was ground and macerated with MeOH (33 L × 5 days × 3 times) at room temperature, followed by filtration. The MeOH was evaporated under reduced pressure and the residual water was removed by freeze-drying to afford the MeOH extract (770 g). A part of the MeOH extract (212 g) was subjected to silica gel column chromatography (Si gel CC) eluting with a hexane–acetone gradient, 100:0 to 0:100 v/v, and an acetone–MeOH gradient, 100:0 to 0:100 v/v. After removal of solvent, 10 fractions (F1–F10) were obtained. Compounds **1** (90.1 mg), **2** (391.0 mg), **3** (38.9 mg), **4** (147.1 mg), **5** (31.2 mg), **6** (25.6 mg), **7** (111.2 mg), **8** (1.48 g), **9** (30.5 mg), **10** (204.7 mg), **11** (909.6 mg), **12** (92.8 mg), **13** (265.9 mg), **14** (1.27 g), **15** (5.9 mg), **16** (359.9 mg), **17** (36.2 mg), **18** (6.2 mg), **19** (12.4 mg), **20** (54.1 mg), **21** (24.0 mg), and **22** (8.7 mg) were isolated from fractions F2–F9 by using column chromatography, gel filtration, and preparative thin layer chromatography along with crystallization techniques (for details, see Supporting Information).

#### (1*R*,3*S*,4a*R*,10a*S*)-7,9-Dihydroxy-1,3,6-trimethyl-3,4,4a,10a-tetrahydro-1*H*-naphtho[2,3-*c*]pyran-5,10-dione or Ventilanone L (**1**)

Orange rods; mp 254–255 °C (CH_2_Cl_2_–hexane); [α]_D_^26^ +55 (*c* 0.40, CHCl_3_); UV (EtOH) λ_max_ (log ε): 243 (4.49), 296 (4.08), 359 (4.14) nm; ECD (0.34 mM, MeOH) nm (Δε): 337 (−0.24), 292 (−3.20), 270 (−3.02), 249 (+1.44), 226 (+4.27): FTIR (Nujol) υ_max_: 3325, 2925, 1693, 1615, 1456, 1193, 1150, 1026, 985, 735 cm^–1^; ^1^H (400 MHz, CDCl_3_) and ^13^C NMR (100 MHz, CDCl_3_) data, Tables 1 and 2; EIMS *m*/*z* 290 [M^+^] (30), 275 (44), 257 (3), 246 (40), 231 (60), 204 (37); HRESIMS *m*/*z* 313.1056 [M + Na]^+^ (calcd for C_16_H_18_O_5_Na, 313.1052).

#### (1*R*,3*S*,4a*R*,10a*S*)-7,9-Dihydroxy-8-methoxy-1,3,6-trimethyl-3,4,4a,10a-tetrahydro-1*H*-naphtho[2,3-*c*]pyran-5,10-dione or Ventilanone M (**2**)

Yellow rods; mp 174–175 °C (CH_2_Cl_2_–hexane); [α]_D_^26^ +76 (*c* 0.28, CHCl_3_); UV (EtOH) λ_max_ (log ε): 206 (2.97), 256 (2.32), 312 (2.86), 355 (2.82) nm; ECD (0.31 mM, MeOH) nm (Δε): 345 (+0.64), 305 (−1.77), 280 (−0.75), 225 (+1.13), 215 (+0.86); FTIR (CHCl_3_) υ_max_: 3497, 3020, 2938, 2852, 1693, 1634, 1582, 1454, 1178, 1076, 1026, 947, 669 cm^–1^; ^1^H (400 MHz, CDCl_3_) and ^13^C NMR (100 MHz, CDCl_3_) data, Tables 1 and 2; EIMS *m*/*z* 320 [M^+^] (94), 305 (52), 276 (49), 261 (100), 233 (45); HRESIMS *m*/*z* 343.1157 [M + Na]^+^ (calcd for C_17_H_20_O_6_Na, 343.1158).

#### (1*R*,3*S*,4a*R*,10a*S*)-7,8-Dimethoxy-9-hydroxy-1,3,6-trimethyl-3,4,4a,10a-tetrahydro-1*H*-naphtho[2,3-*c*]pyran-5,10-dione or Ventilanone N (**3**)

Pale yellow rods; mp 116–117 °C (CH_2_Cl_2_–hexane); [α]_D_^26^ +26 (*c* 0.16, CHCl_3_); UV (EtOH) λ_max_ (log ε): 205 (3.99), 248 (4.29), 297 (3.70), 359 (3.71) nm; ECD (0.33 mM, MeOH) nm (Δε): 340 (+0.20), 293 (−1.97), 241 (+1.17), 220 (+2.03); FTIR (CHCl_3_) υ_max_: 3363, 2975, 2873, 1690, 1635, 1567, 1468, 1442, 1295, 1175, 1121, 1076, 928, 794 cm^–1^; ^1^H (400 MHz, CDCl_3_) and ^13^C NMR (100 MHz, CDCl_3_) data, Tables 1 and 2; EIMS *m*/*z* 334 [M^+^] (100), 319 (56), 290 (28), 275 (69), 260 (14), 233 (23), 215 (12); HRESIMS *m*/*z* 357.1309 [M + Na]^+^ (calcd for C_18_H_22_O_6_Na, 357.1314).

#### (1*R*,3*S*)-7,9-Dimethoxy-8-hydroxy-1,3,6-trimethyl-3,4-dihydro-1*H*-naphtho[2,3-*c*]pyran-5,10-dione or Ventilanone O (**4**)

Yellow needles; mp 136–137 °C (CH_2_Cl_2_–hexane); [α]_D_^26^ +310 (*c* 0.24, CHCl_3_); UV (EtOH) λ_max_ (log ε): 216 (4.61), 271 (4.53), 385 (3.75) nm; ECD (0.33 mM, MeOH) nm (Δε): 287 (+8.27), 243 (+2.22); FTIR (Nujol) υ_max_: 3378, 2923, 2855, 1660, 1651, 1556, 1455, 1200, 1137, 1109, 988, 722 cm^–1^; ^1^H (400 MHz, CDCl_3_) and ^13^C NMR (100 MHz, CDCl_3_) data, Tables 1 and 2; EIMS *m*/*z* 332 [M^+^] (100), 317 (79), 302 (26), 299 (29), 289 (18), 274 (25), 259 (18), 245 (13); HRESIMS *m*/*z* 355.1163 [M + Na]^+^ (calcd for C_18_H_20_O_6_Na, 355.1158).

#### (1*R*,3*S*)-7,9-Dimethoxy-8-hydroxymethyl-1,3,6-trimethyl-3,4-dihydro-1*H*-naphtho[2,3-*c*]pyran-5,10-dione or Ventilanone P (**5**)

Yellow needles; mp 126–127 °C (CH_2_Cl_2_–hexane); [α]_D_^26^ +320 (*c* 28, CHCl_3_); UV (EtOH) λ_max_ (log ε): 213 (4.66), 258 (4.40), 376 (3.78) nm; ECD (0.38 mM, MeOH) nm (Δε): 283 (+8.57), 257 (+6.44); FTIR (KBr) υ_max_: 3459, 3395, 2983, 2942, 2905, 1658, 1635, 1556, 1452, 1200, 1096, 961, 721 cm^–1^; ^1^H (400 MHz, CDCl_3_) and ^13^C NMR (100 MHz, CDCl_3_) data, Tables 1 and 2; EIMS *m*/*z* 346 [M^+^] (72), 331 (35), 313 (100), 299 (12), 287 (36), 271 (25), 255 (19), 241 (17); HRESIMS *m*/*z* 369.1313 [M + Na]^+^ (calcd for C_19_H_22_O_6_Na, 369.1314).

#### 7-Acetyl-2,3-dimethoxy-8-hydroxy-6-methylnaphthalene-1,4-dione or Ventilanone Q (**6**)

Orange rods; mp 146–147 °C (CH_2_Cl_2_–hexane); UV (EtOH) λ_max_ (log ε): 225 (4.65), 296 (4.37), 417 (4.04) nm; FTIR (KBr) υ_max_: 3426, 2953, 2850, 1692, 1617, 1598, 1475, 1438, 1295, 1012, 985, 784, 722 cm^–1^; ^1^H (400 MHz, CDCl_3_) and ^13^C NMR (100 MHz, CDCl_3_) data, Table 3; EIMS *m*/*z* 290 [M^+^] (59), 275 (100), 260 (8), 257 (42), 232 (29), 217 (16), 204 (6), 176 (13); HRESIMS *m*/*z* 313.0691 [M + Na]^+^ (calcd for C_15_H_14_O_6_Na, 313.0688).

#### 7-Acetyl-4,8-dihydroxy-2,3-dimethoxy-4,6-dimethylnaphthalen-1(1*H*)-one or Ventilanone R (**7**)

Pale yellow needles; mp 139–140 °C (CH_2_Cl_2_–hexane); UV (EtOH) λ_max_ (log ε): 206 (4.58), 250 (4.49), 350 (4.26) nm; FTIR (CHCl_3_) υ_max_: 3579, 3016, 2957, 2836, 1695, 1614, 1482, 1456, 1120, 975, 819, 669 cm^–1^; ^1^H (400 MHz, CDCl_3_) and ^13^C NMR (100 MHz, CDCl_3_) data, Table 3; EIMS *m*/*z* 306 [M^+^] (13), 291 (26), 274 (14), 273 (15), 259 (32), 245 (5), 231 (8), 43 (100); HRESIMS *m*/*z* 329.1002 [M + Na]^+^ (calcd for C_16_H_18_O_6_Na, 329.1001).

#### Single Crystal X-ray Diffraction Analysis of Compound **1**

X-ray crystallographic data for C_16_H_18_O_5_: MW = 290.32; 0.35 × 0.30 × 0.30 mm; monoclinic; space group *P*2_1_ (No. 4); *a* = 4.8054(11) Å, *b* = 7.5474(6) Å, *c* = 19.632(3) Å, β = 94.39(2)°, *V* = 709.9(2) Å^3^, *Z* = 2, *T* = 273(2) K, μ(Cu Kα) = 0.837 mm^–1^, *D*_*x*_ = 1.358 g/mm^3^; reflections collected/unique 6864/6030; number of observations [*I* > 2σ(*I*)] 2480; R1 = 0.0324, wR2 = 0.0809 (all data); Flack parameter = 0.12(8). X-ray crystallographic data were measured on a Bruker APEX2 diffractometer equipped with a graphite monochromated Cu Kα radiation source (λ = 1.54178 Å). The structure was solved by using Olex2^37^ and refined with the olex2.refine refinement package using Gauss–Newton minimization.^38,39^ The crystallographic data were deposited at the Cambridge Crystallographic Data Centre under the reference number CCDC 2006115. Copies of the data can be obtained, free of charge, upon application to the Director, CCDC, 12 Union Road, Cambridge, CB2 1EZ, U.K.

### Syncytium Assay

A cell-based assay using the ^ΔTat/Rev^MC99 virus and 1A2 cell line system was used.^40^ The experiment was carried out in triplicate, starting at the final concentration of 3.9 μg/mL to 125 μg/mL of compound. Virus control and cell control wells contained neither compound nor the virus; cytotoxicity control wells containing cells with the compound and a positive control, i.e., azidothymidine (AZT), were included. The results were expressed as the effective concentration (EC_50_) to reduce syncytium formation by 50%. A colorimetric cytotoxicity assay using XTT tetrazolium salt and phenazine methosulfate as described by Weislow et al. was also performed in parallel.^41^ The procedure was similar to the syncytium assay, but the virus was replaced by medium and tests were performed in duplicate. Control wells included medium, drug, and cell controls. After the soluble formazan developed, the optical density at *A*_450_ was measured with a reference at *A*_650_. The result was expressed as 50% inhibitory concentration (IC_50_).

### Cytotoxic Assay

The cytotoxicities of the isolated compounds were determined by using the standard *in vitro* sulforhodamine B (SRB) assay in 96-well microtiter plates.^42,43^ Ellipticine was used as a positive control. Seven cell lines were employed, including KKU-M213, human cholangiocarcinoma; FaDu, human pharyngeal carcinoma; HT-29, human colorectal adenocarcinoma; MDA-MB-231, human mammary gland adenocarcinoma; A-549, human lung carcinoma; SH-SY5Y, human neuroblastoma; and MMNK1, human cholangiocyte cell lines. The potency for cytotoxicity was expressed as the half-maximal inhibitory concentration (IC_50_) values.

## Supporting Information Available

1D and 2D NMR spectra of compounds **1**–**7**; ^1^H and ^13^C data for **16** and **21**; HPLC separation of (+)-**7** and (−)-**7**; overlaid ECD spectra of (+)-**7** and (−)-**7**; comparison of optical rotation of **7** with related compounds (PDF)
X-ray data file for **1** (CIF)

## Supplementary Material

np2c00980_si_001.pdf

np2c00980_si_002.cif

## Dedication

Dedicated to Dr. Mary J. Garson, The University of Queensland, for her pioneering work on bioactive natural products.
