# Supplementary material for: Pyranonaphthoquinones and Naphthoquinones from the Stem Bark of Ventilago harmandiana and Their Anti-HIV-1 Activity
Source: J Nat Prod. 2023 Feb 14;86(3):498–507. doi: 10.1021/acs.jnatprod.2c00980 (PMC10043937; doi:10.1021/acs.jnatprod.2c00980)
Supplement: Supplementary file 1 — np2c00980_si_001.pdf [file np2c00980_si_001.pdf]

Supporting Information

Pyranonaphthoquinones and Naphthoquinones from the Stem Bark of *Ventilago harmandiana* and Their Anti-HIV-1 Activity

Suwannee Saisin[†], Kanda Panthong[‡], Sakchai Hongthong[§], Chutima Kuhakarn[†], Sariyarach Thanasansurapong[†], Arthit Chairoungdua[⊥], Kanoknetr Suksen[⊥], Radeekorn Akkarawongsapat[∥], Chanita Napaswad[∥], Samran Prabpai[†], Narong Nuntasaen[▽], and Vichai Reutrakul[*,†]

[†]Department of Chemistry and Center of Excellence for Innovation in Chemistry (PERCH-CIC), Faculty of Science, Mahidol University, Rama VI Road, Bangkok 10400, Thailand

[‡]Division of Physical Sciences and Center of Excellence for Innovation in Chemistry (PERCH-CIC), Faculty of Science, Prince of Songkla University, Songkhla 90112, Thailand

[§]Division of Chemistry, Faculty of Science and Technology, Rajabhat Rajanagarindra University, Chachoengsao 24000, Thailand

[⊥]Department of Physiology, Faculty of Science, Mahidol University, Rama VI Road, Bangkok 10400, Thailand

[∥]Department of Microbiology, Faculty of Science, Mahidol University, Rama VI Road, Bangkok 10400, Thailand

[▽]The Forest Herbarium National Park, Wildlife and Plant Conservation Department, Ministry of Natural Resources and Environment, Bangkok 10900, Thailand

* Corresponding author.

E-mail address: vichai.reu@mahidol.ac.th (V. Reutrakul)

Content		Page
**Experimental for Extraction and Isolation**		S4–S6
**Table S1**	Cytotoxic activity of isolated compounds from the stem bark of *V. harmandiana*	S7
**Figure S1**	Compounds **1**−**22** isolated from the stem bark of *V. harmandiana*	S8
**Figure S2**	^{1}H NMR (400 MHz) spectrum of compound **1** in $CDCl_3$	S9
**Figure S3**	^{13}C NMR (100 MHz) spectrum of compound **1** in $CDCl_3$	S10
**Figure S4**	COSY spectrum of compound **1**	S11
**Figure S5**	HMQC spectrum of compound **1**	S12
**Figure S6**	HMBC spectrum of compound **1**	S13
**Figure S7**	NOESY spectrum of compound **1**	S14
**Figure S8**	^{1}H NMR (400 MHz) spectrum of compound **2** in $CDCl_3$	S15
**Figure S9**	^{13}C NMR (100 MHz) spectrum of compound **2** in $CDCl_3$	S16
**Figure S10**	COSY spectrum of compound **2**	S17
**Figure S11**	HMQC spectrum of compound **2**	S18
**Figure S12**	HMBC spectrum of compound **2**	S19
**Figure S13**	NOESY spectrum of compound **2**	S20
**Figure S14**	^{1}H NMR (400 MHz) spectrum of compound **3** in $CDCl_3$	S21
**Figure S15**	^{13}C NMR (100 MHz) spectrum of compound **3** in $CDCl_3$	S22
**Figure S16**	COSY spectrum of compound **3**	S23
**Figure S17**	HMQC spectrum of compound **3**	S24
**Figure S18**	HMBC spectrum of compound **3**	S25
**Figure S19**	NOESY spectrum of compound **3**	S26
**Figure S20**	^{1}H NMR (400 MHz) spectrum of compound **4** in $CDCl_3$	S27
**Figure S21**	^{13}C NMR (100 MHz) spectrum of compound **4** in $CDCl_3$	S28
**Figure S22**	COSY spectrum of compound **4**	S29
**Figure S23**	HMQC spectrum of compound **4**	S30
**Figure S24**	HMBC spectrum of compound **4**	S31
**Figure S25**	NOESY spectrum of compound **4**	S32
**Figure S26**	^{1}H NMR (400 MHz) spectrum of compound **5** in $CDCl_3$	S33
**Figure S27**	^{13}C NMR (100 MHz) spectrum of compound **5** in $CDCl_3$	S34
**Figure S27**	COSY spectrum of compound **5**	S35
**Figure S28**	HMQC spectrum of compound **5**	S36
**Figure S30**	HMBC spectrum of compound **5**	S37
**Figure S31**	NOESY spectrum of compound **5**	S38

Content		Page
**Figure S32**	^{1}H NMR (400 MHz) spectrum of compound **6** in $CDCl_3$	S39
**Figure S33**	^{13}C NMR (100 MHz) spectrum of compound **6** in $CDCl_3$	S40
**Figure S34**	COSY spectrum of compound **6**	S41
**Figure S35**	HMQC spectrum of compound **6**	S42
**Figure S36**	HMBC spectrum of compound **6**	S43
**Figure S37**	NOESY spectrum of compound **6**	S44
**Figure S38**	^{1}H NMR (400 MHz) spectrum of compound **7** in $CDCl_3$	S45
**Figure S39**	^{13}C NMR (100 MHz) spectrum of compound **7** in $CDCl_3$	S46
**Figure S40**	COSY spectrum of compound **7**	S47
**Figure S41**	HMQC spectrum of compound **7**	S48
**Figure S42**	HMBC spectrum of compound **7**	S49
**Figure S43**	NOESY spectrum of compound **7**	S50
**Figure S44**	^{1}H NMR (400 MHz) spectrum of compound **16** in $CDCl_3$	S51
**Figure S45**	^{13}C NMR (100 MHz) spectrum of compound **16** in $CDCl_3$	S52
**Figure S46**	^{1}H NMR (400 MHz) spectrum of compound **21** in acetone-d_6	S53
**Figure S47**	^{13}C NMR (100 MHz) spectrum of compound **21** in acetone-d_6	S54
**Figure S48**	HPLC separation of (+)-**7** and (–)-**7**	S55
**Figure S49**	Overlayed ECD spectra of (+)-**7** and (–)-**7**	S56
**Figure S50**	The comparison of optical rotation of **7** with related compounds	S57
**References**		S58

Experimental for Extraction and Isolation

The stem bark (9.7 kg) of *V. harmandiana* was air-dried, grounded and macerated with MeOH (33 L × 5 days × 3 times) at room temperature, followed by filtration. The MeOH extract (770 g) was obtained after evaporation of the filtrate and removal of the trace solvent by freeze-drying. The portion of MeOH extract (212 g) was subjected on siliga gel column chromatography (Si gel CC) eluted with gradient solvent system A (hexane-acetone; 100:0 to 0:100 v/v and acetone-MeOH; 100:0 to 0:100 v/v) to afford ten fractions (F1–F10). Fraction F2 [1.39 g, eluted by acetone-hexane (6:94, v/v)] was separated on Si gel CC (gradient solvent system A) leading to eight subfractions (F2.1-F2.8). After purification of the combination of subfractions F2.2, F2.3, and F2.4 (175.1 mg) using preparative TLC (acetone-hexane (1:99, v/v) as eluent), followed by recrystallization from CH_2Cl_2-hexane to give chrysophanol (**17**) (36.2 mg). Fraction F3 [1.98 g, eluted by acetone-hexane (8:92, v/v)] was isolated on Si gel CC eluted with gradient solvent system B (hexane-CH_2Cl_2; 100:0 to 0:100 v/v and CH_2Cl_2-MeOH; 100:0 to 0:100 v/v) leading to three subfractions (F3.1-F3.3). After recrystallization using CH_2Cl_2-hexane of F3.2 (1.77 g), the crystals were indentified to be 8,8′-methylenebis(7,10-dihydroxy-1,3,6,6-tetramethyl-3,4-dihydro-1*H*-benzo[*g*]isochromen-9-one) (**16**) (202.6 mg). After removal of solvent, the mother liquor was purified by preparative TLC, eluted with acetone-hexane (15:85, v/v) to give four isolated bands (F3.2-ML.1-F3.2-ML.4). Ventilanone N (**3**) (38.9 mg) and ventilanone B (**9**) (30.5 mg) were obtained from subfractions F3.2-ML.2 and F3.2-ML.3, respectively. Fraction F4 [2.35 g, eluted by acetone-hexane (8:92, v/v)] was separated by Si gel CC (gradient solvent system A) leading to seven subfractions (F4.1-F4.7). Due to the similarity of their TLC characteristics, Subfractions F4.2 and F4.3 were combined (818.1 g) and purified by preparative TLC, eluted with acetone-hexane (15:85, v/v), followed by recrystallization from CH_2Cl_2-hexane to afford additional amount of **16** (157.3 mg). The precipitation was obtained after dissolution of subfraction F4.4 (1.53 g) with the mixture of acetone in hexane. After purification the precipitate by preparative TLC, eluted with acetone-hexane (15:85, v/v) and crystallization from CH_2Cl_2-hexane, ventilanone C (**10**) (204.7 mg) and ventilanone M (**2**) (51.3 mg) were obtained. The solid obtained after the removal of the solvent from the mother liquor was separated using preparative TLC, eluted with acetone-hexane (15:85, v/v) and crystallization with CH_2Cl_2-

hexane to give ventilanone E (**12**) (92.8 mg), and 4-hydroxy-5,6,7-trimethoxy-2-methylanthraquinone (**18**) (6.2 mg). Fraction F5 [1.15 g, eluted with acetone-hexane (1:9, v/v)] was separated by Si gel CC (gradient solvent system A) to give four subfractions (F5.1–F5.4). After recrystallization from CH_2Cl_2-hexane, additional amount of **2** (153.6 mg) was obtained from subfraction F5.2 (250.9 mg). Subfraction F5.3 (572.8 mg) was separated by preparative TLC, eluted with acetone-hexane (1:15, v/v), gave four subfractions (F5.3.1-F5.3.4). After recrystallization using CH_2Cl_2-hexane, compound **2** (186.1 mg) and ventilanone A (**8**) (118.5 mg) were obtained from subfractions F5.3.2 and F5.3.3, respectiviely. Fraction F6 [1.72 g, eluted by acetone-hexane (1:9, v/v)] was separated Si gel CC (gradient solvent system A) leading to four subfractions (F6.1-F6.4). After purification by using preparative TLC, eluted with acetone-haxane (1:9, v/v), followed by recrystallization from CH_2Cl_2-hexane, compound **8** (348.0 mg) and ventilanone H (**14**) (418.3 mg) were obtained form subfractions F6.2 (731.0 mg) and F6.3 (711.2 mg). Fraction F7 [6.20 g, eluted by acetone-hexane (14:86, v/v)] was separated by Si gel CC (gradient solvent system A) gave seven subfractions (F7.1-F7.7). After recrystallization from subfraction F7.2 (3.53 g) using CH_2Cl_2-hexane, compound **14** (814.5 mg) was obtained. After removal the solvent, the mother liquor of subfraction F7.2 (2.53 g) was purified by Si gel CC (gradient system A) gave three subfractions (F7.2-ML.1-F7.2-ML-3). Subfraction F7.2-ML.2 (871.6 mg) was purificatrion using preparative TLC, eluted with acetone-hexane (15:85, v/v), followed by recrystallized from CH_2Cl_2-hexane gave **8** (730.6 mg). Subfraction F7.3 (749.5 mg) was separated on Sephadex LH-20 CC, eluted with CH_2Cl_2-hexane (2:1, v/v), CH_2Cl_2 and MeOH yielded three subfractions (F7.3.1-F7.3.3). After separation of subfraction F7.3.2 (93.8 mg) using preparative TLC, eluted with acetone-hexane (1:9, v/v), followed by recrystallized from CH_2Cl_2-hexane, ventilanone Q (**6**) (25.6 mg) and 5-hydroxy-1,3-dimethoxy-7-methylanthraquinone (**19**) (12.4 mg) were obtained. Due to the similarity of their TLC characteristics, subfractions F7.4 and F7.5 were combined (1.71 g) and separated by Si gel CC (gradient solvent system A) leading to five subfractions (F7.4.1-F7.4.5). After separation of subfraction F7.4.1 (722.7 mg) using preparative TLC, eluted with acetone-haxane (1:19, v/v), followed by recrystallization from CH_2Cl_2-hexane, ventilanone P (**5**) (31.2 mg) was obtained. Ventilanone O (**4**) (147.1 mg) and ventilanone D (**11**) (361.4 mg)

were obtained from subfractions F7.4.2 (517.87 mg) after purification using preparative TLC, eluted with acetone-haxanes (1:19, v/v), followed by recrystallization from CH_2Cl_2-hexane. Subfraction F7.4.3 (78.9 mg) was purified by using preparative TLC, eluting with acetone-haxanes (1:19, v/v), followed by recrystallization from CH_2Cl_2-hexane leding to demethylmacrosporine I (**20**) (15.0 mg). Fraction F8 [8.34 g, eluted with acetone-hexane (7:43 to 1:3, v/v)] was separated on Si gel CC (gradient solvent system A) leading to eight subfractions (F8.1-F8.8). After crystallization of subfraction F8.2 (244.1 mg) from CH_2Cl_2-hexane, compound **8** (288.4 mg) was obtained. Subfraction F8.3 (500.9 mg) was purified by preparative TLC, eluted with acetone-hexane (1:4, v/v), leading to four subfractions (F8.3.1-F8.3.4). Obtusifolin (**22**) (8.7 mg), **14** (38.7 mg), and **1** (90.1 mg) were obtained from subfractions F8.3.1, F8.3.2, and F8.3.3, respectively. Subfraction F8.4 (2.44 g) was separated by Si gel CC (gradient solvent system A) leading to three subfractions (F8.34.1-F8.4.3). Subfraction F8.4.2 (1.27 g) was purified by Si gel CC (gradient solvent system B) leading to four subfractions (F8.4.2.1-F8.4.2.4). Ventilanone D (**11**) (909.6 mg) was obtained from subfractions F8.4.2.2 and **20** (39.1 mg) and ventilanone I (**15**) (5.9 mg) were obtained from subfraction F8.4.2.3 after purification using preparative TLC, eluted with acetone-hexane (1:4, v/v), followed by crystallization from CH_2Cl_2-hexane. Ventilanone R (**7**) (131.5 mg) and ventilanone G (**13**) (100.0 mg) were obtained from subfractions F8.6 (2.87 g) and F8.7 (454.6 mg) after crystallization from CH_2Cl_2-hexane. Fraction F9 [9.92 g, eluted with acetone-hexane (3:7 to 3:2, v/v)] was separated by Si gel CC (gradient solvent system A) to give six subfractions (F9.1-F9.6). After purification of subfraction F9.4 (654.8 mg) using preparative TLC, eluted with acetone-hexane (1:4, v/v), followed by recrystallization from CH_2Cl_2-hexane to give **7** (111.2 mg). Ventilanone G (**13**) (165.9 mg) and 2,6-dihydroxy-1,7,8-trimethoxy-3-methylanthraquinone (**21**) (24.0 mg) were obtained from subfraction F9.5 (1.0 g) eluted with acetone-hexane (1:4, v/v), followed by recrystallization from CH_2Cl_2-hexane. In addition, enantiomer, (+)-7 (t_R = 17 min) and (–)-7 (t_R = 20 min) was obtained after HPLC separation (Chiralpak OD-H column, IPA-hexane (5:95) as mobile phase, flow rate 1 mL/min).

Table S1. Cytotoxic activity of isolated compounds from the stem bark of *V. harmandiana*

Compounds	IC_{50} (μM)						
	KKU-M213	FaDu	HT-29	MDA-MB-231	A549	SH-SY5Y	MNNK-1
**1**	–	–	–	–	–	–	–
**2**	–	–	–	–	–	–	–
**4**	–	–	–	–	–	–	–
**5**	–	–	–	–	–	–	–
**7**	25	26	42	40	42	23	20
**8**	–	–	–	–	–	–	–
**11**	–	–	–	–	–	–	–
**13**	–	–	–	–	–	37	–
**14**	–	–	–	–	–	–	–
**16**	–	–	–	–	–	–	–
**Ellipticine**	2.2	1.7	2.1	2.2	1.2	1.9	1.8

Results are expressed as IC_{50} <50 μM is considered active. (–) = IC_{50} > 50 μM.
KKU-M213 = human cholangiocarcinoma cells; FuDa = human pharyngeal carcinoma cells; HT-29 = human colorectal adenocarcinoma cells; MDA-MB-231 = human mammary gland adenocarcinoma cells; A-549 = human lung carcinoma cells; SH-SY5Y = human neuroblastoma cells; MMNK1= human cholangiocyte cells.
Ellipticine was used as a positive control

Figure S1 Compounds **1**−**22** isolated from the stem bark of *V. harmandiana*.

(A) New compounds

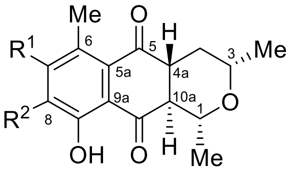

1; R^1 = OH, R^2 = H
2; R^1 = OH, R^2 = OMe
3; R^1 = OMe, R^2 = OMe

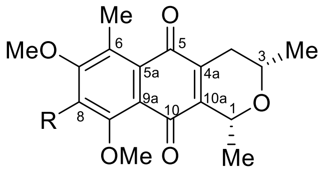

4; R = OH
5; R = CH_2OH

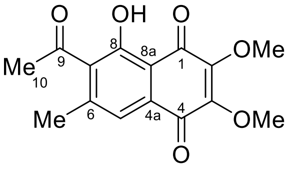

6

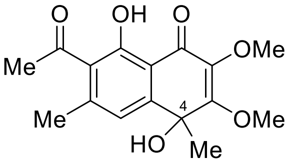

7

(B) Previously reported compounds

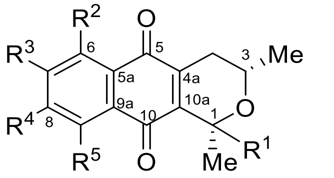

	R^1	R^2	R^3	R^4	R^5
**8**	H	Me	OH	OMe	OMe
**9**	H	Me	OMe	OMe	OMe
**10**	H	Me	OH	OMe	OH
**11**	H	H	OH	OMe	OMe
**12**	H	H	OMe	OMe	OMe
**13**	H	Me	OH	H	OH
**14**	H	Me	OH	H	OMe
**15**	OH	H	OH	OMe	OMe

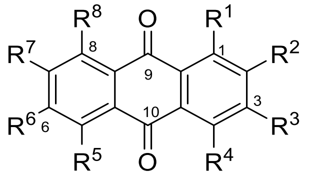

	R^1	R^2	R^3	R^4	R^5	R^6	R^7	R^8
**17**	OH	H	Me	H	H	H	H	OH
**18**	H	Me	H	OH	OMe	OMe	OMe	H
**19**	OMe	H	OMe	H	OH	H	Me	H
**20**	OH	H	OH	H	H	OH	Me	H
**21**	OMe	OH	Me	H	H	OH	OMe	OMe
**22**	OMe	OH	Me	H	H	H	H	OH

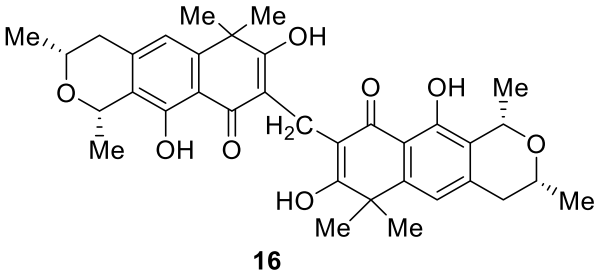

16

Figure S2 ^{1}H NMR (400 MHz) spectrum of compound **1** in $CDCl_3$

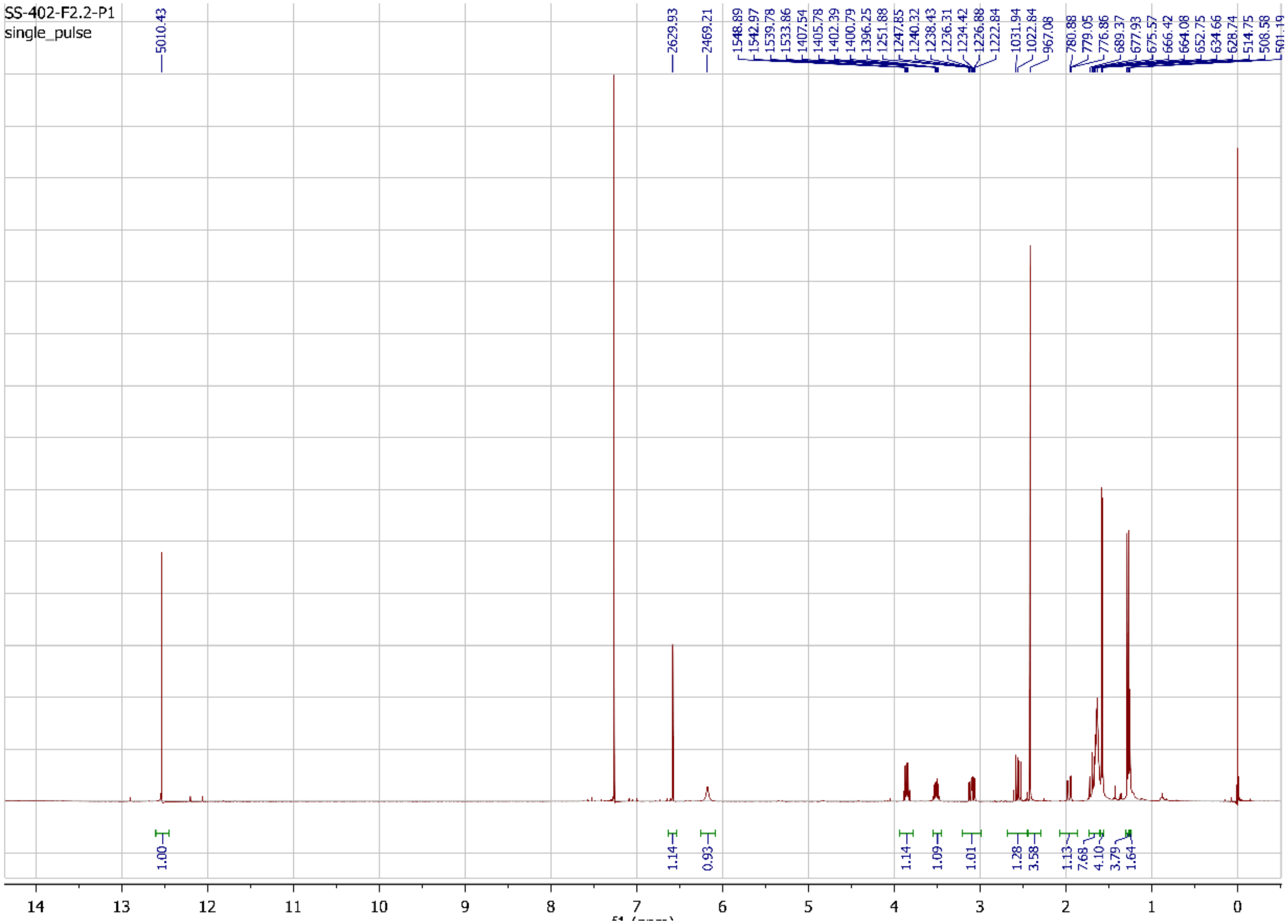

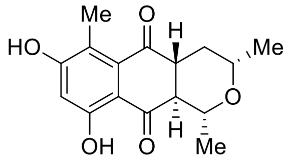

1

Figure S3 ^{13}C NMR (100 MHz) spectrum of compound **1** in $CDCl_3$

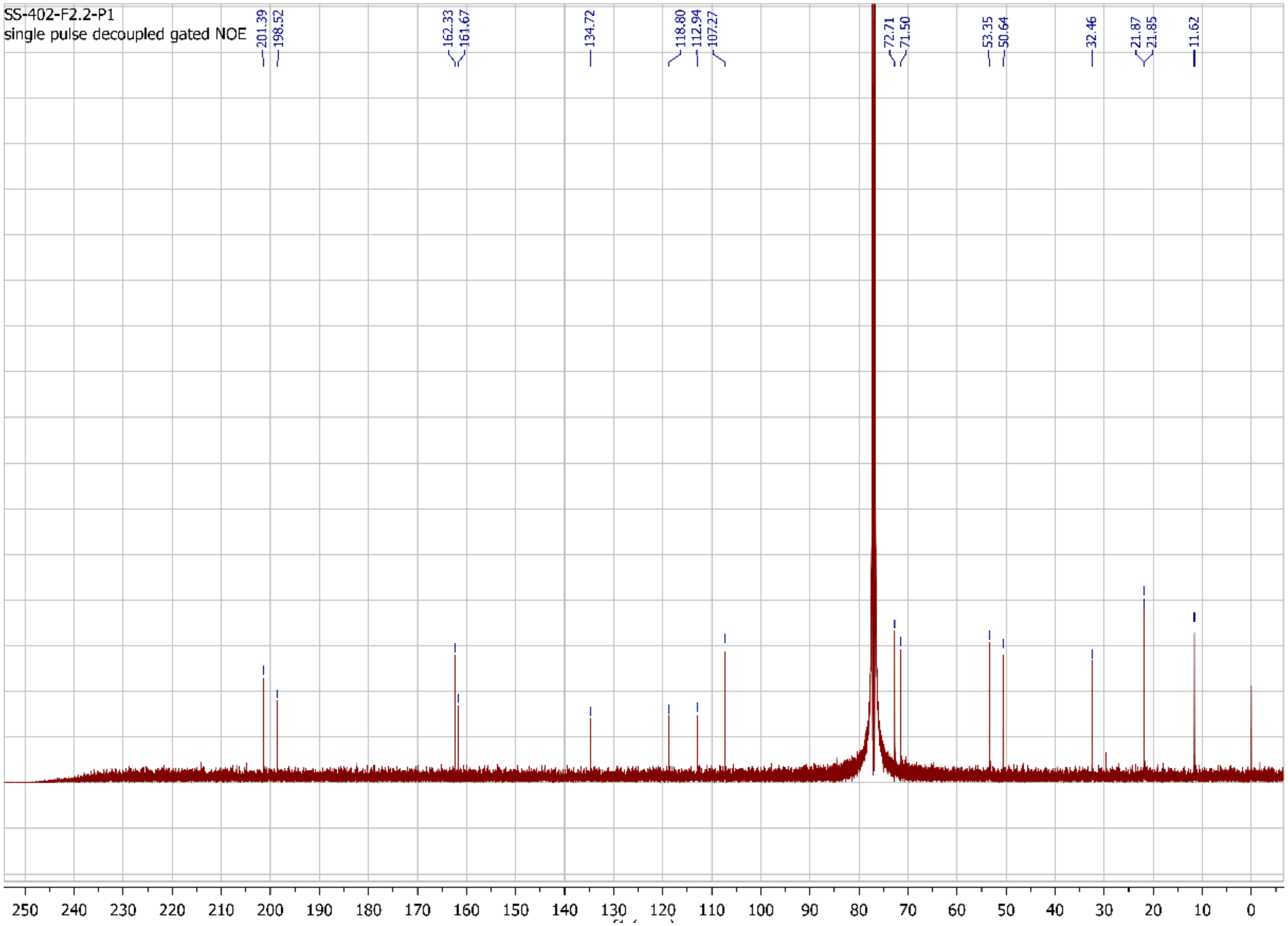

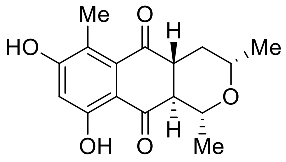

1

Figure S4 COSY spectrum of compound **1**

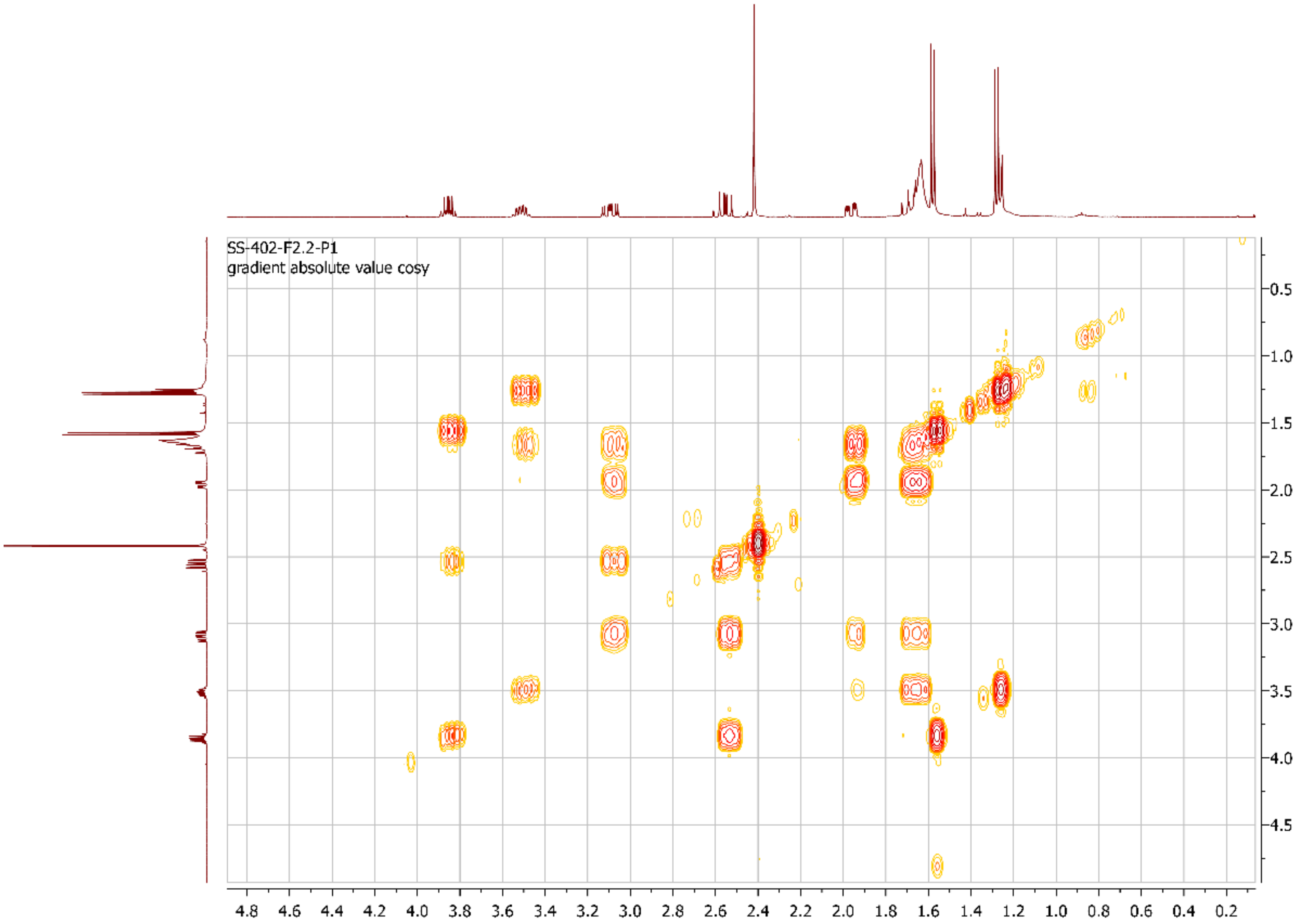

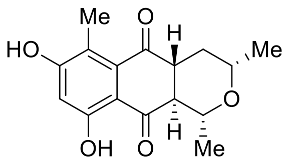

1

Figure S5 HMQC spectrum of compound **1**

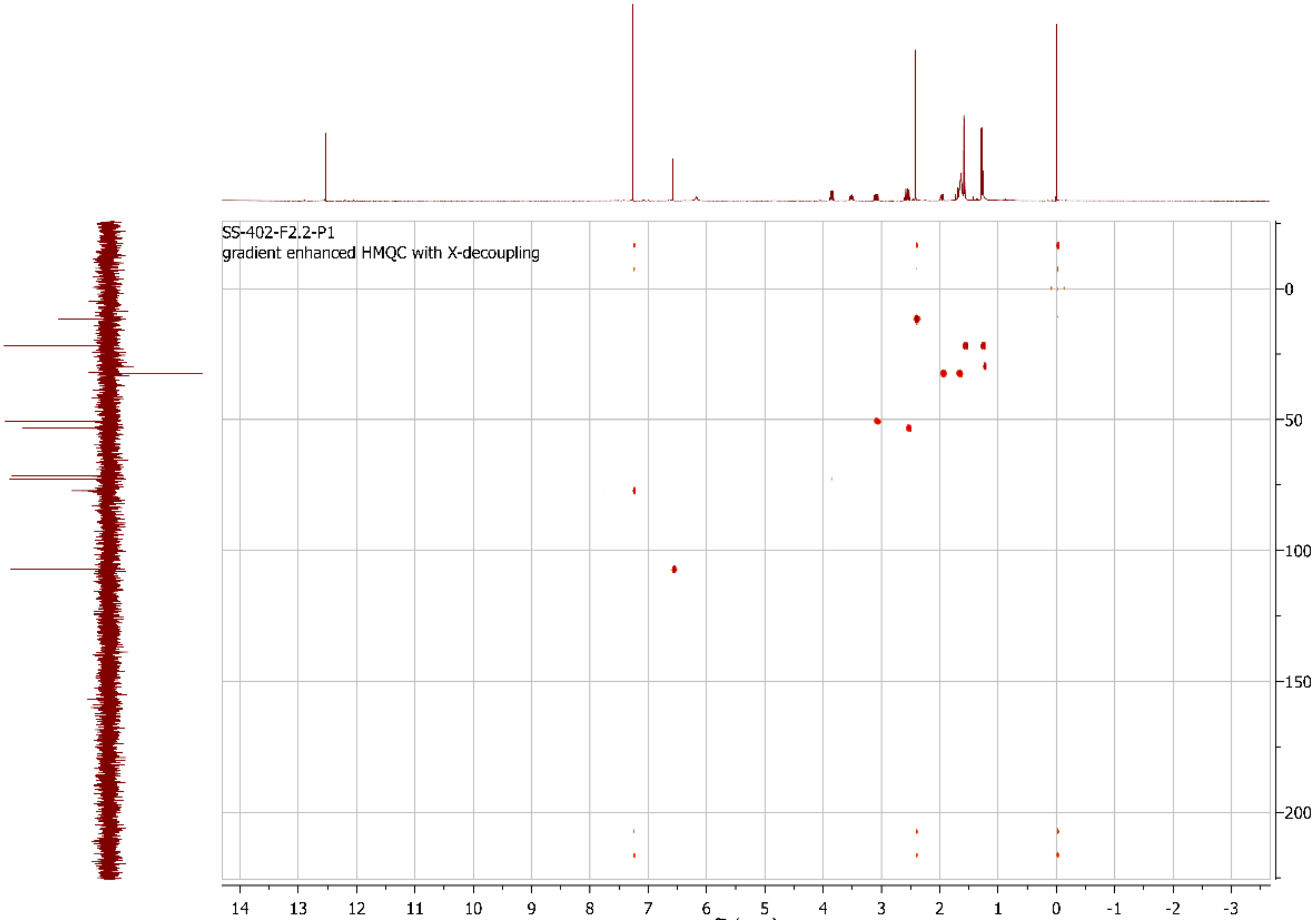

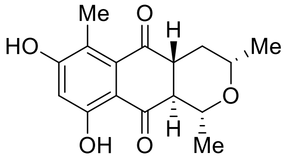

1

Figure S6 HMBC spectrum of compound **1**

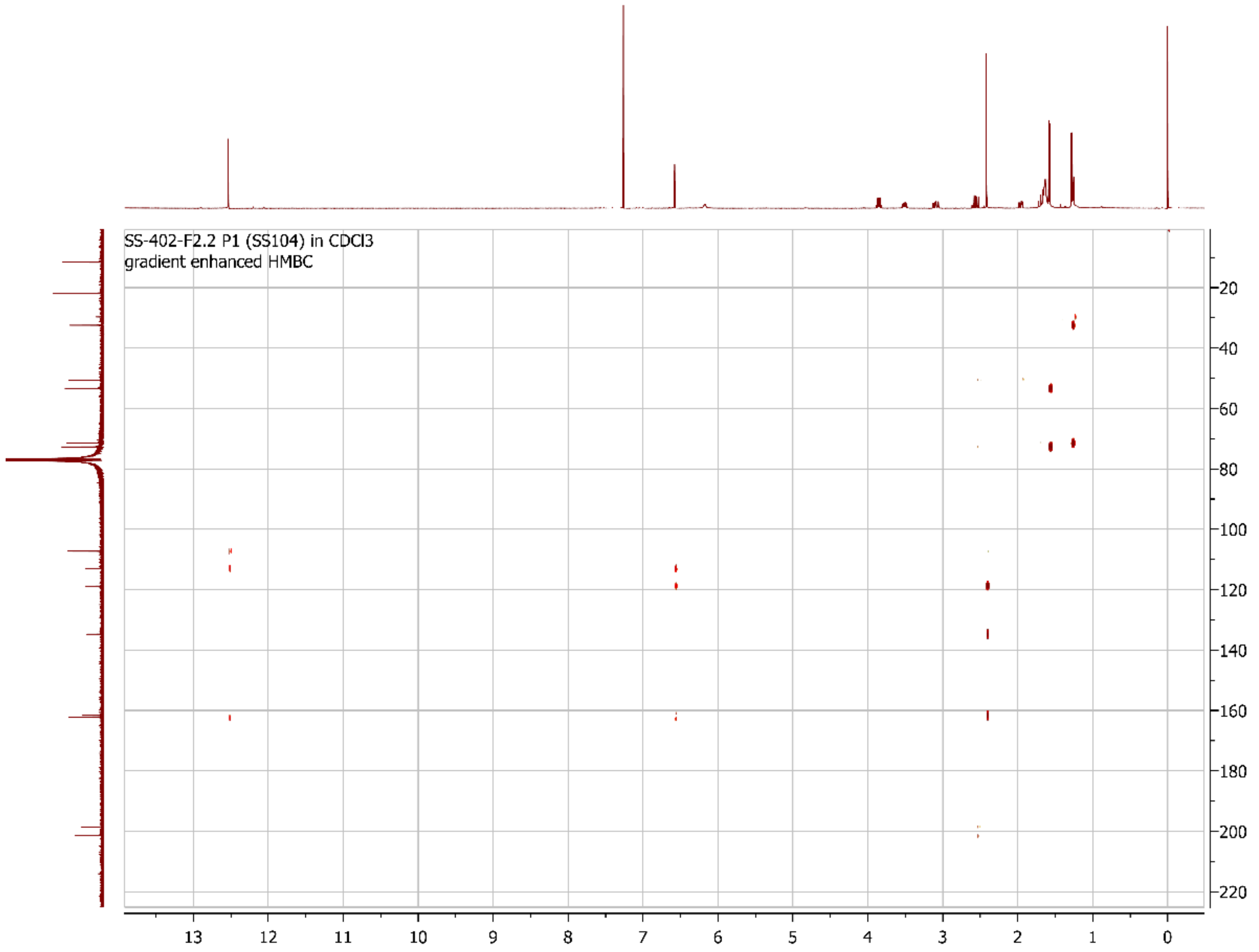

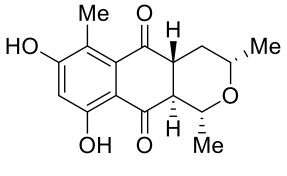

1

Figure S7 NOESY spectrum of compound **1**

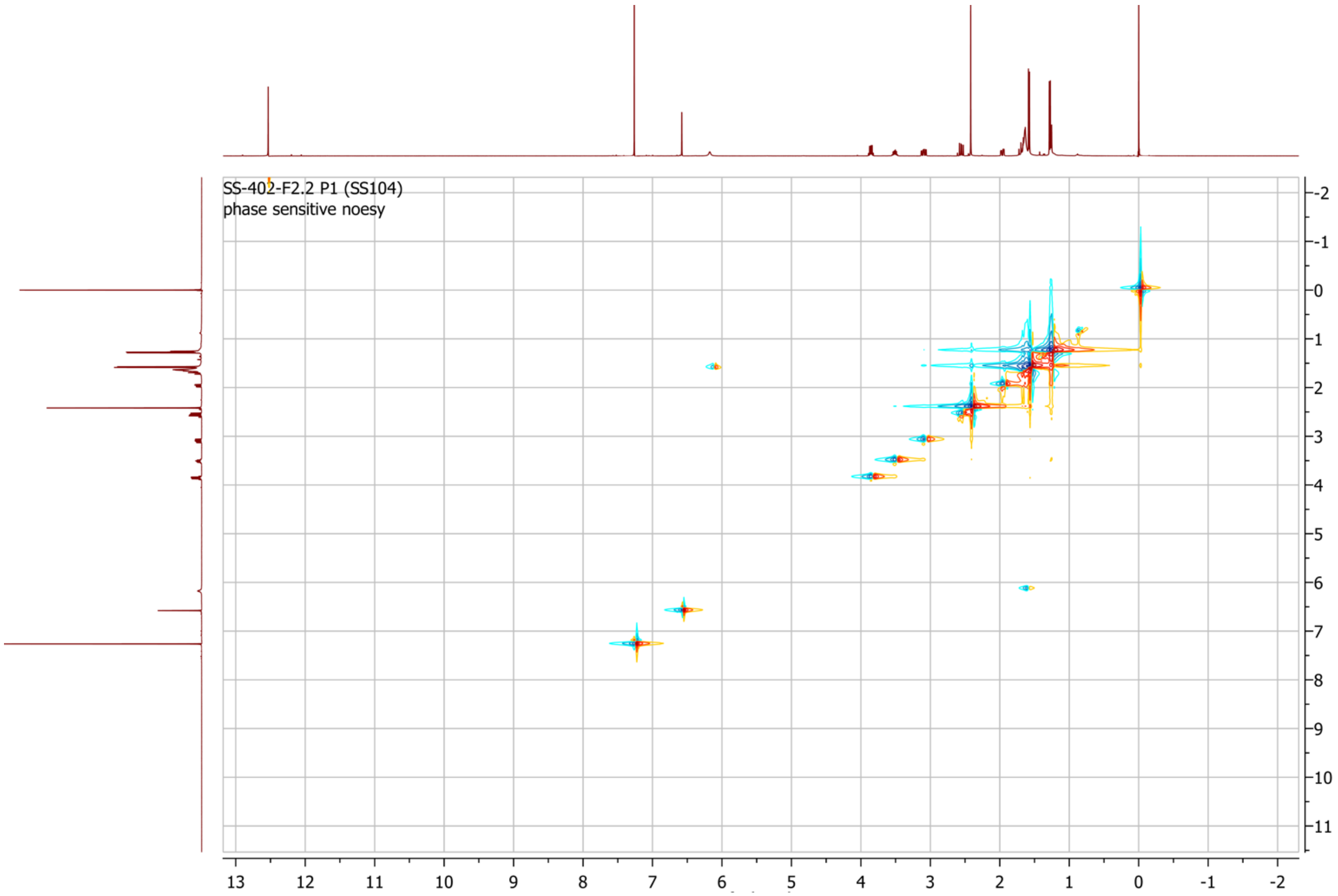

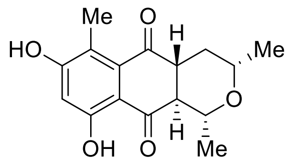

1

Figure S8 ^{1}H NMR (400 MHz) spectrum of compound **2** in $CDCl_3$

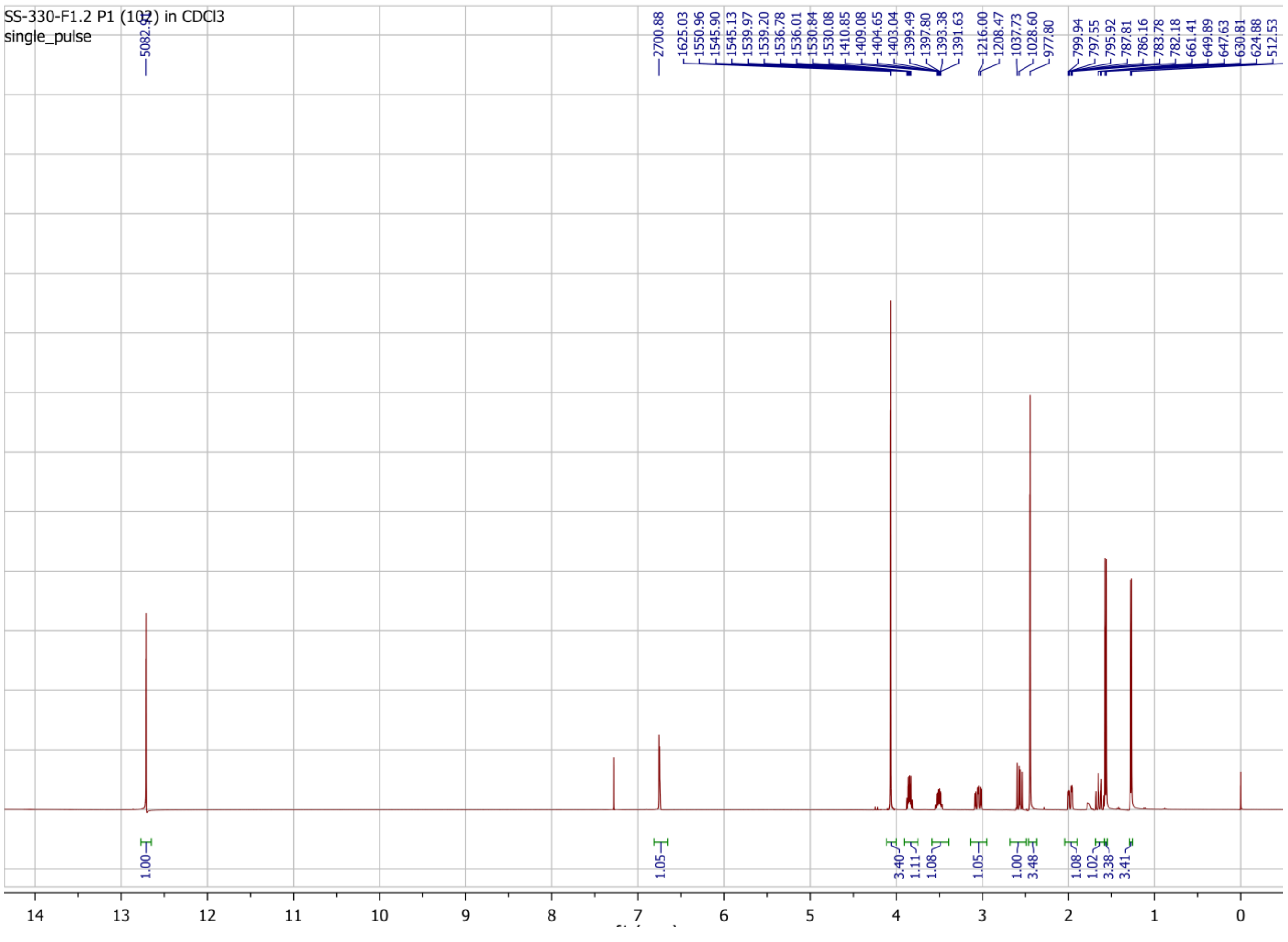

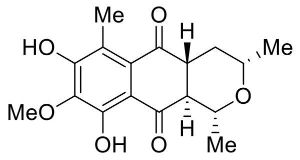

2

Figure S9 ^{13}C NMR (100 MHz) spectrum of compound **2** in $CDCl_3$

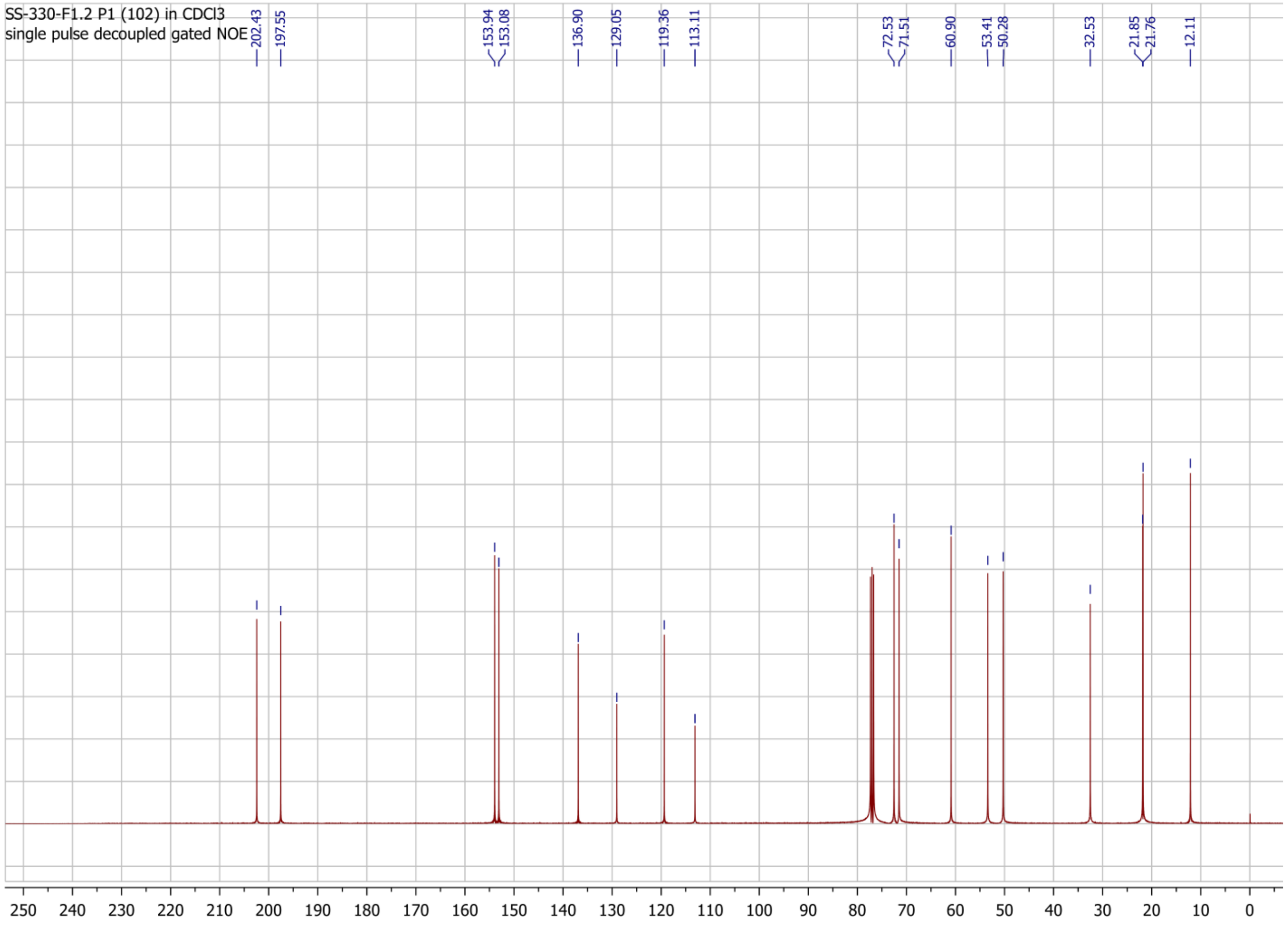

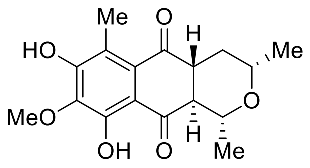

2

Figure S10 COSY spectrum of compound **2**

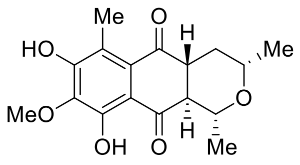

2

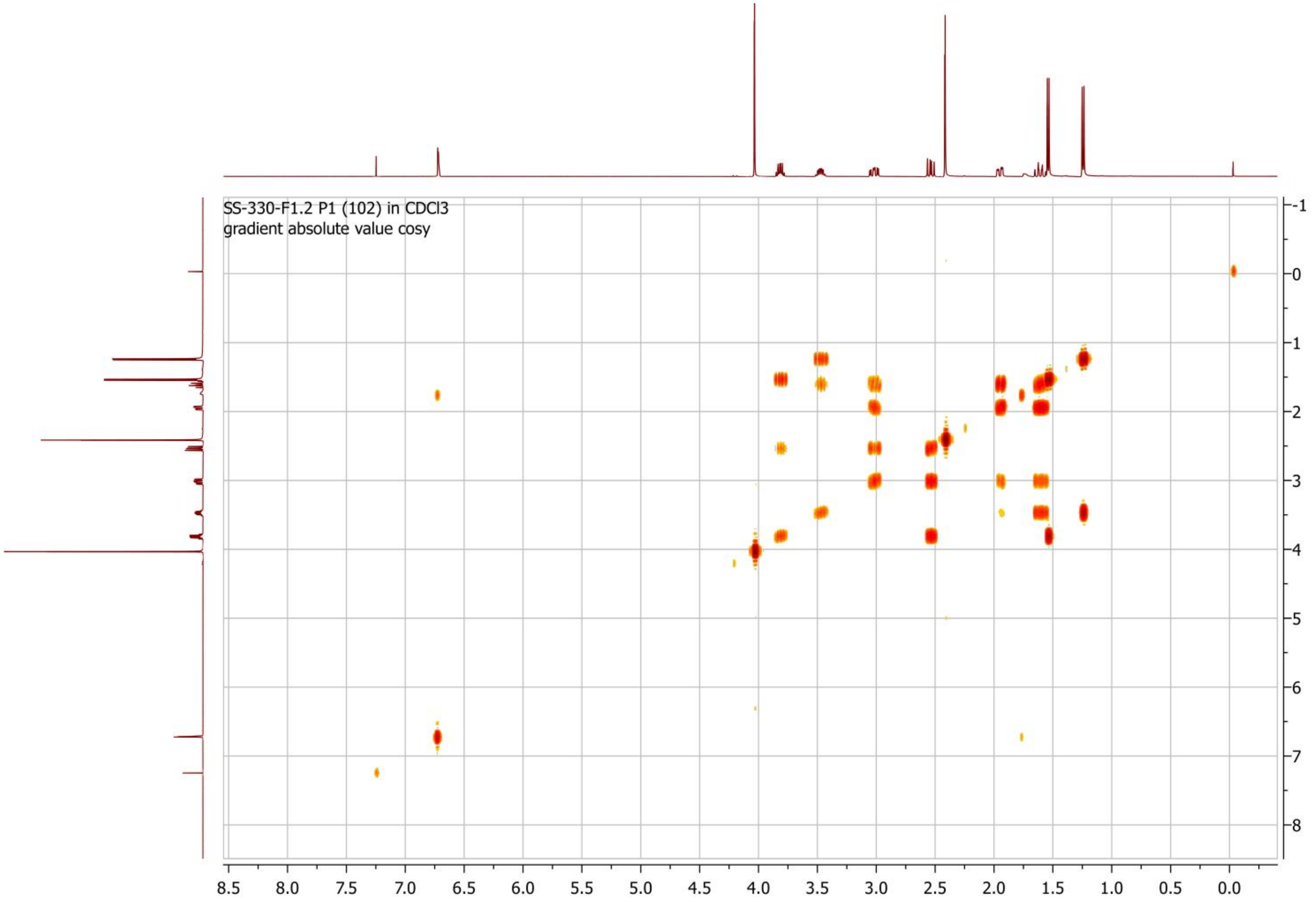

Figure S111 HMQC spectrum of compound **2**

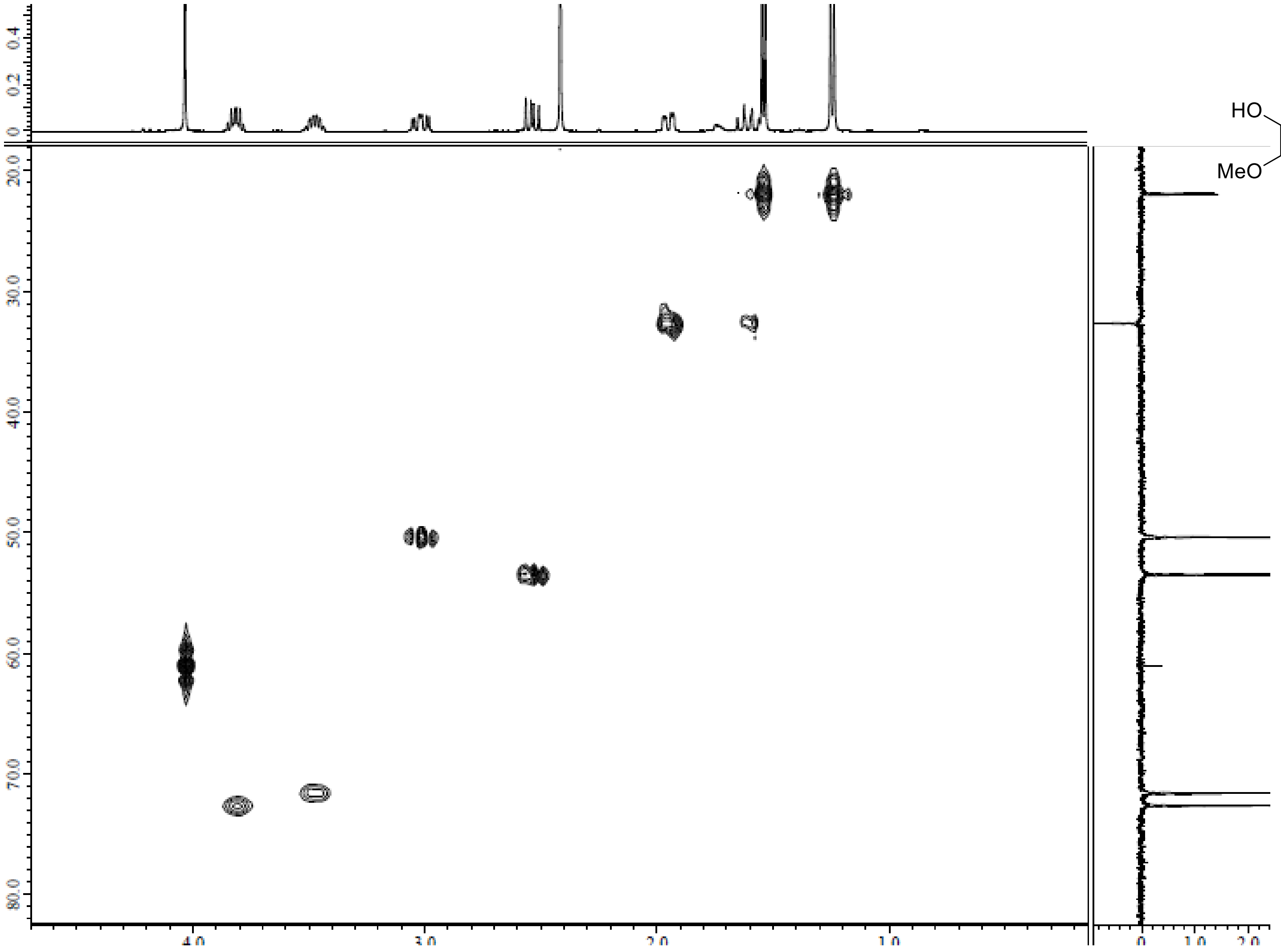

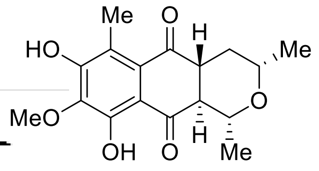

2

Figure S12 HMBC spectrum of compound **2**

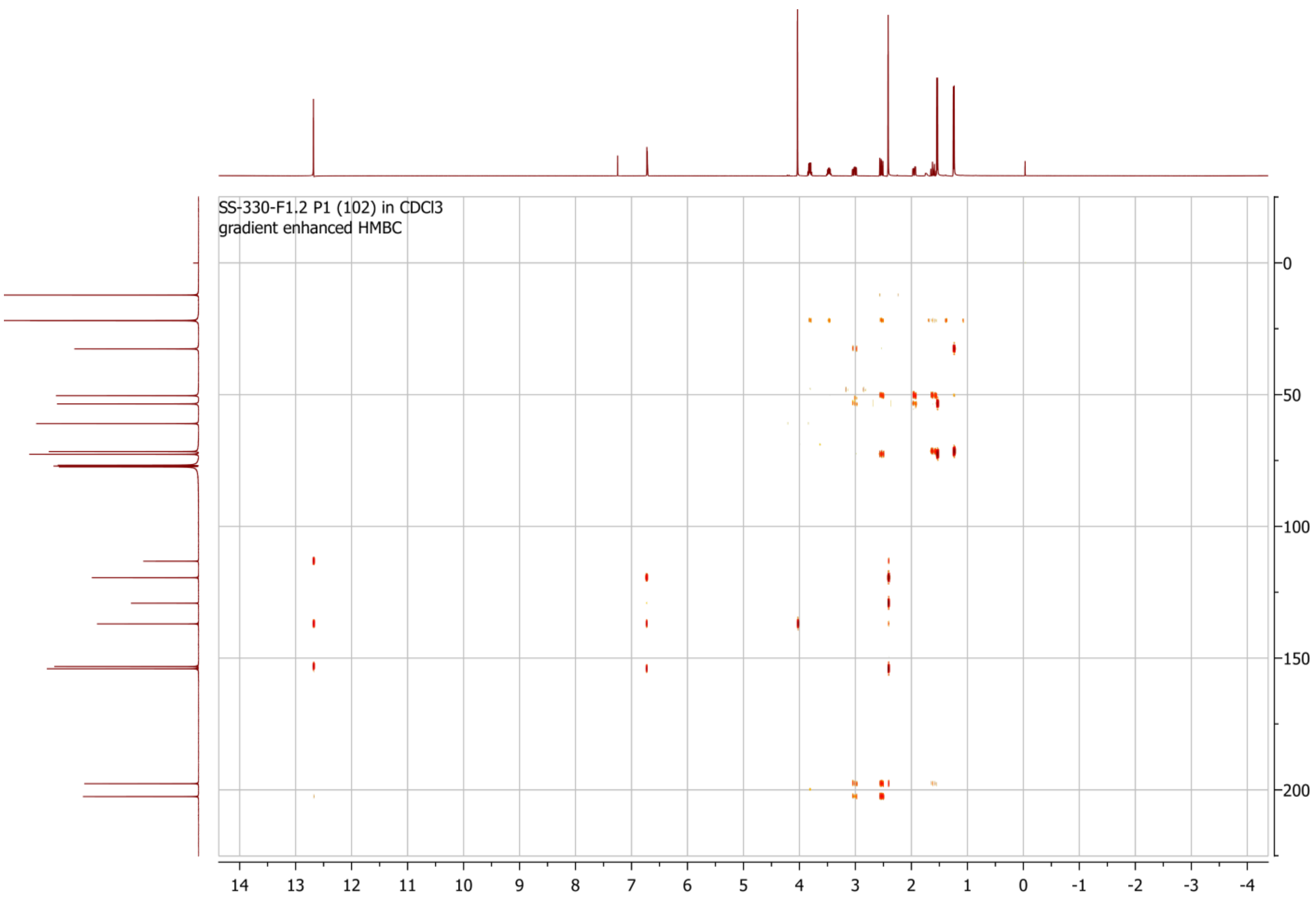

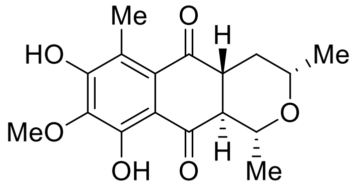

2

Figure S13 NOESY spectrum of compound **2**

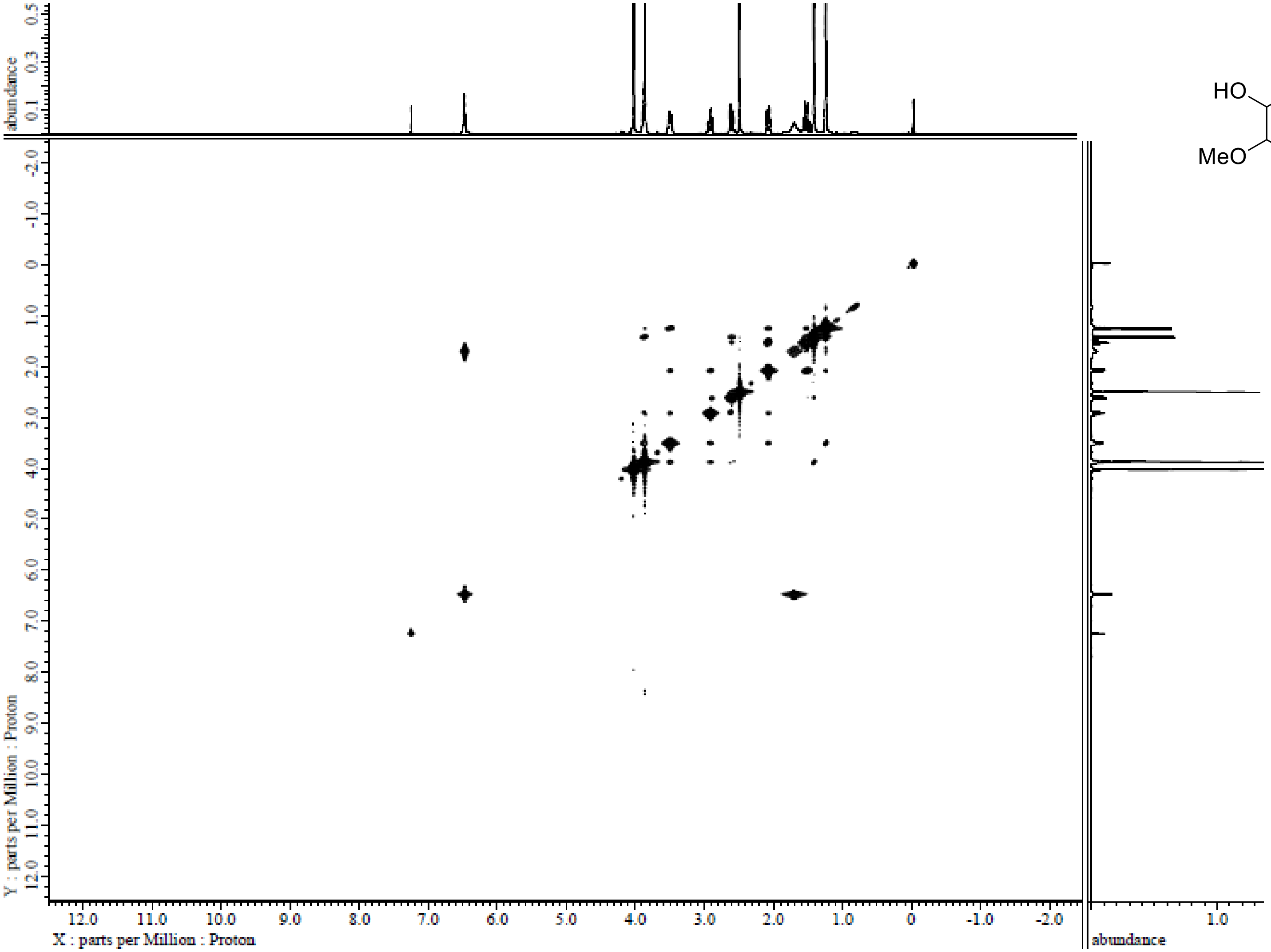

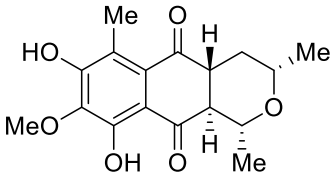

2

Figure S14 ^{1}H NMR (400 MHz) spectrum of compound **3** in $CDCl_3$

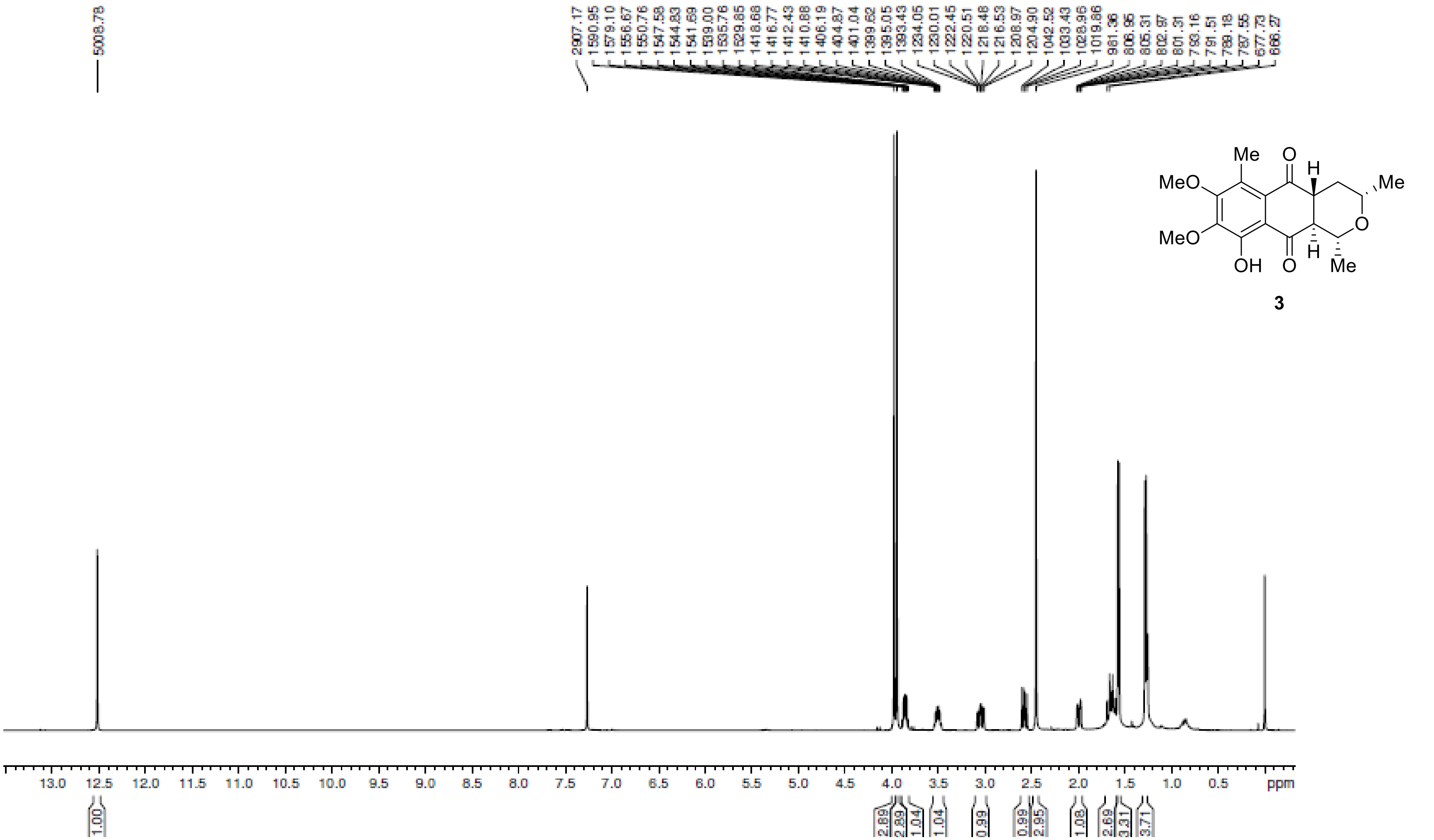

Figure S15 ^{13}C NMR (400 MHz) spectrum of compound **3** in $CDCl_3$

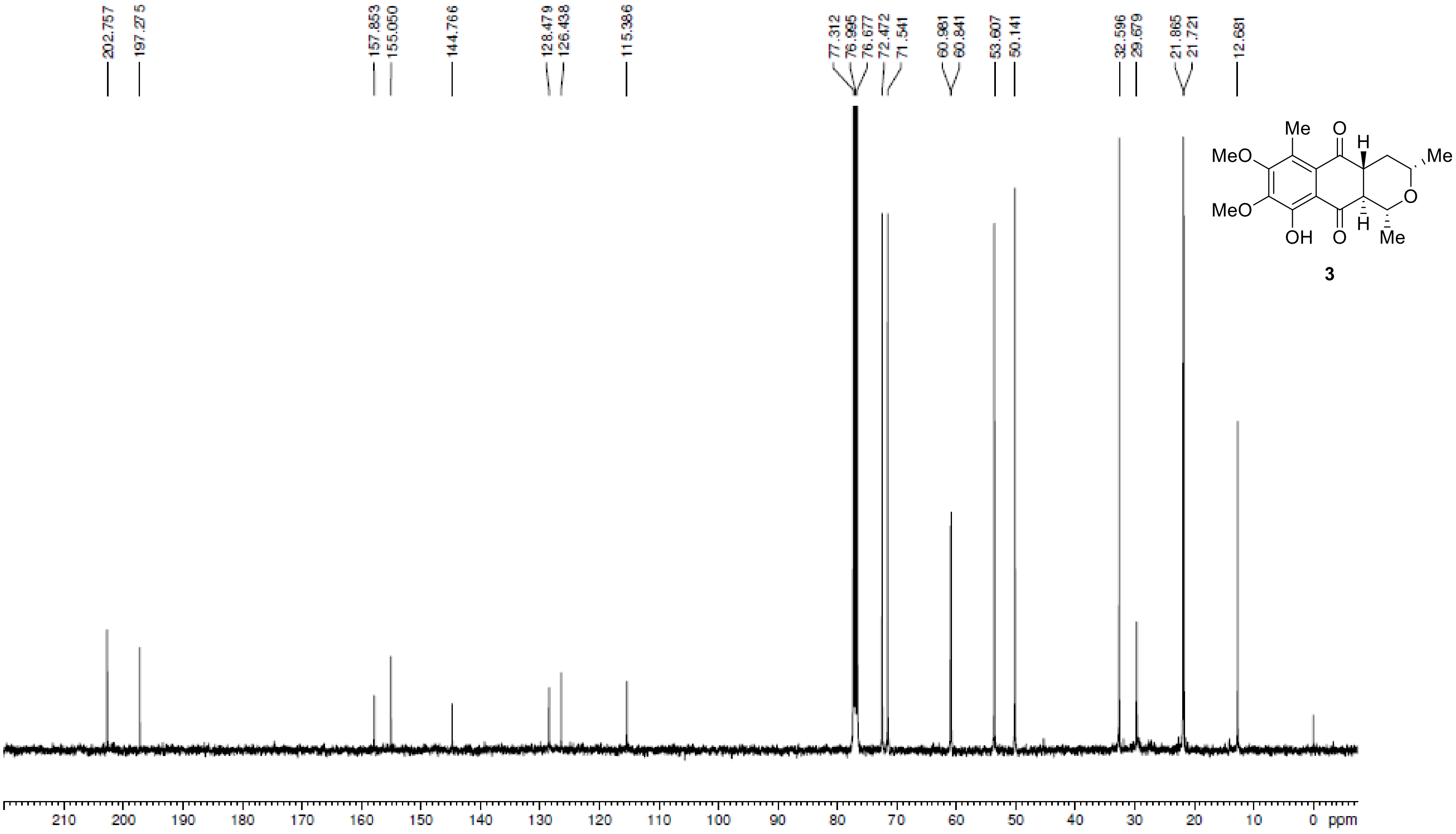

Figure S16 COSY spectrum of compound **3**

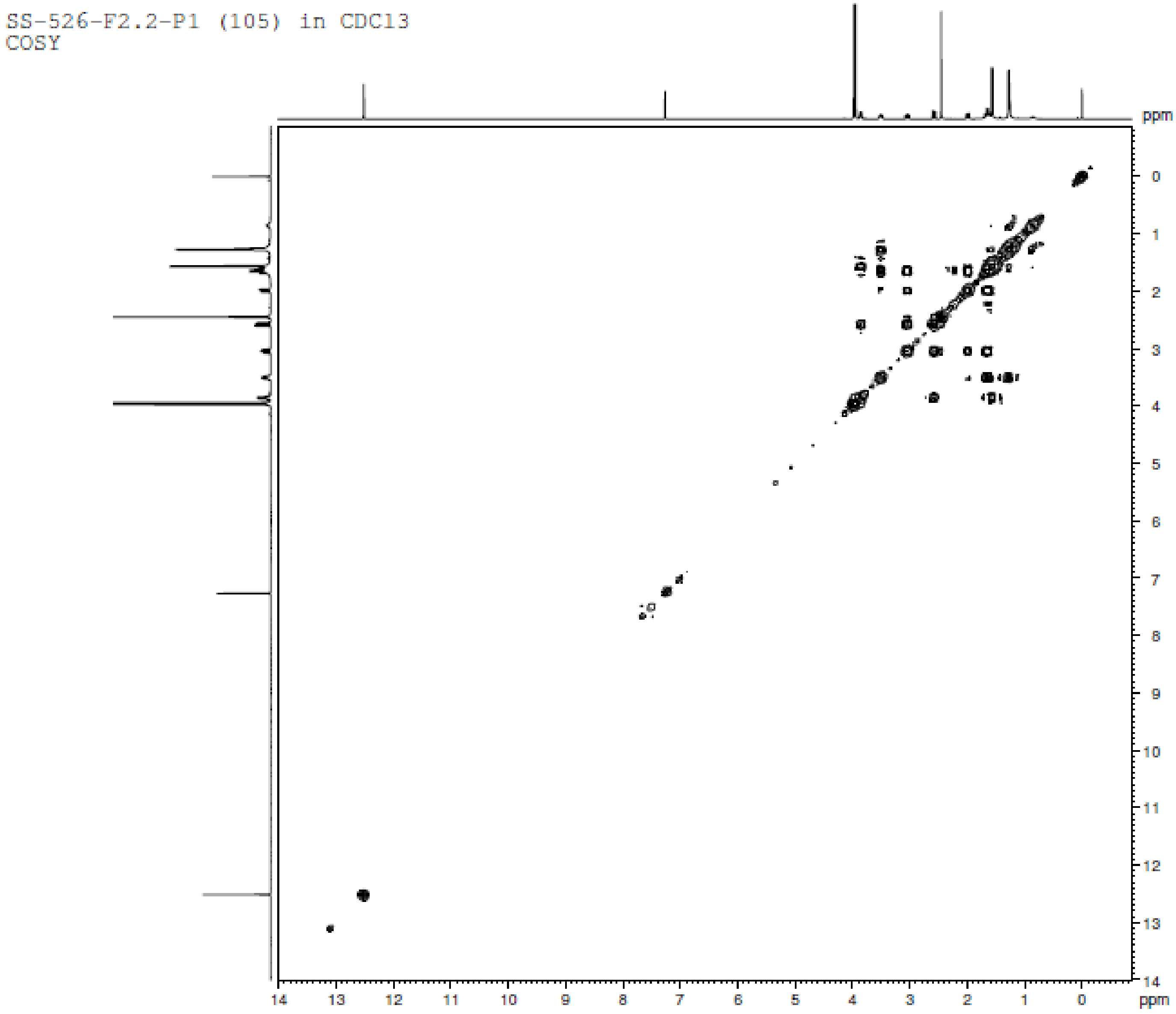

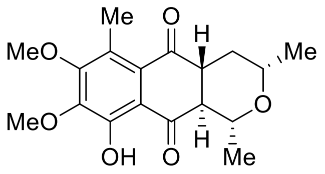

3

Figure S17 HMQC spectrum of compound **3**

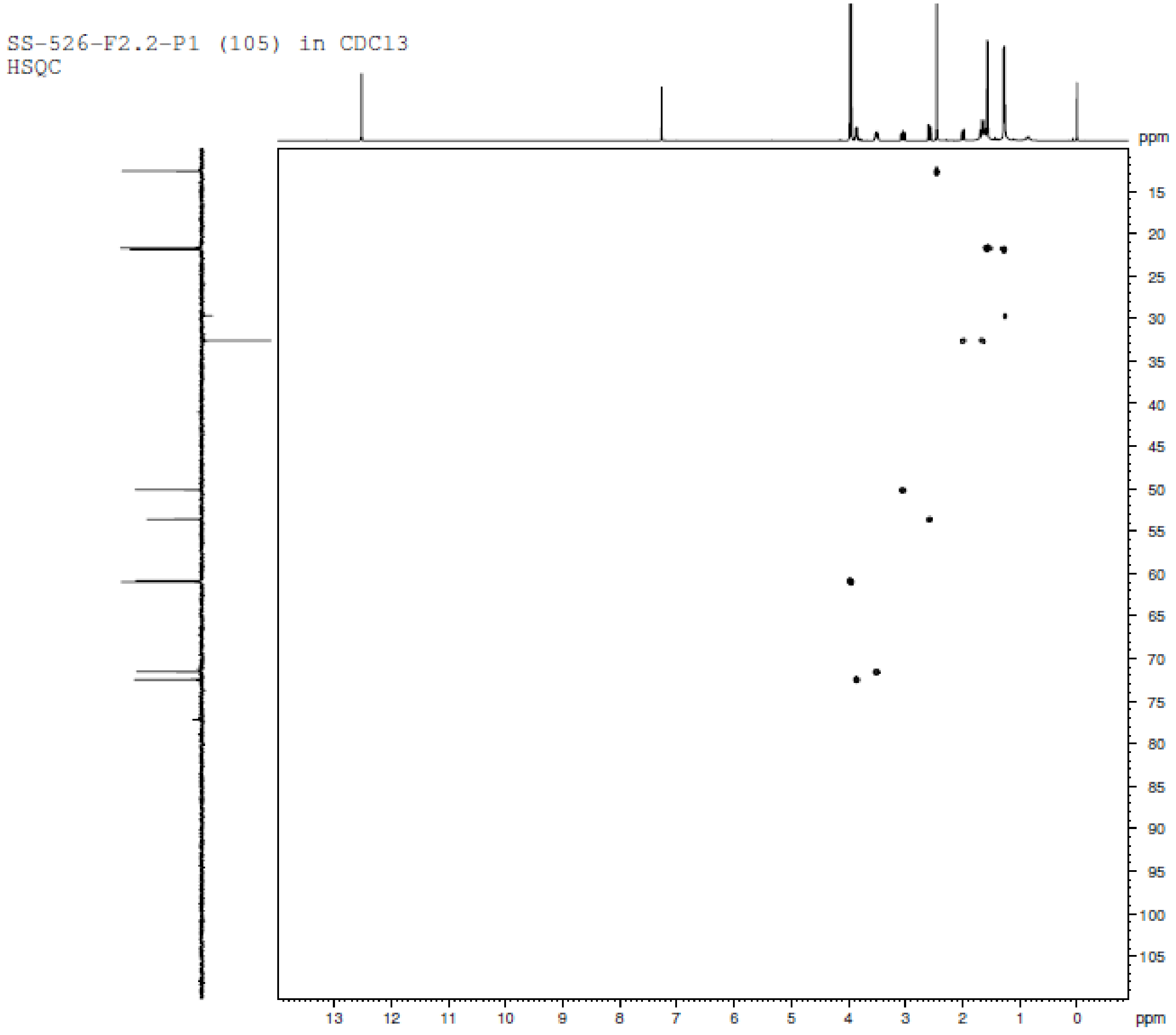

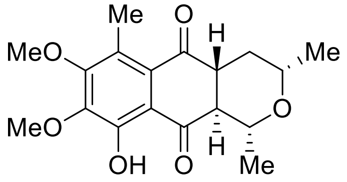

3

Figure S18 HMBC spectrum of compound **3**

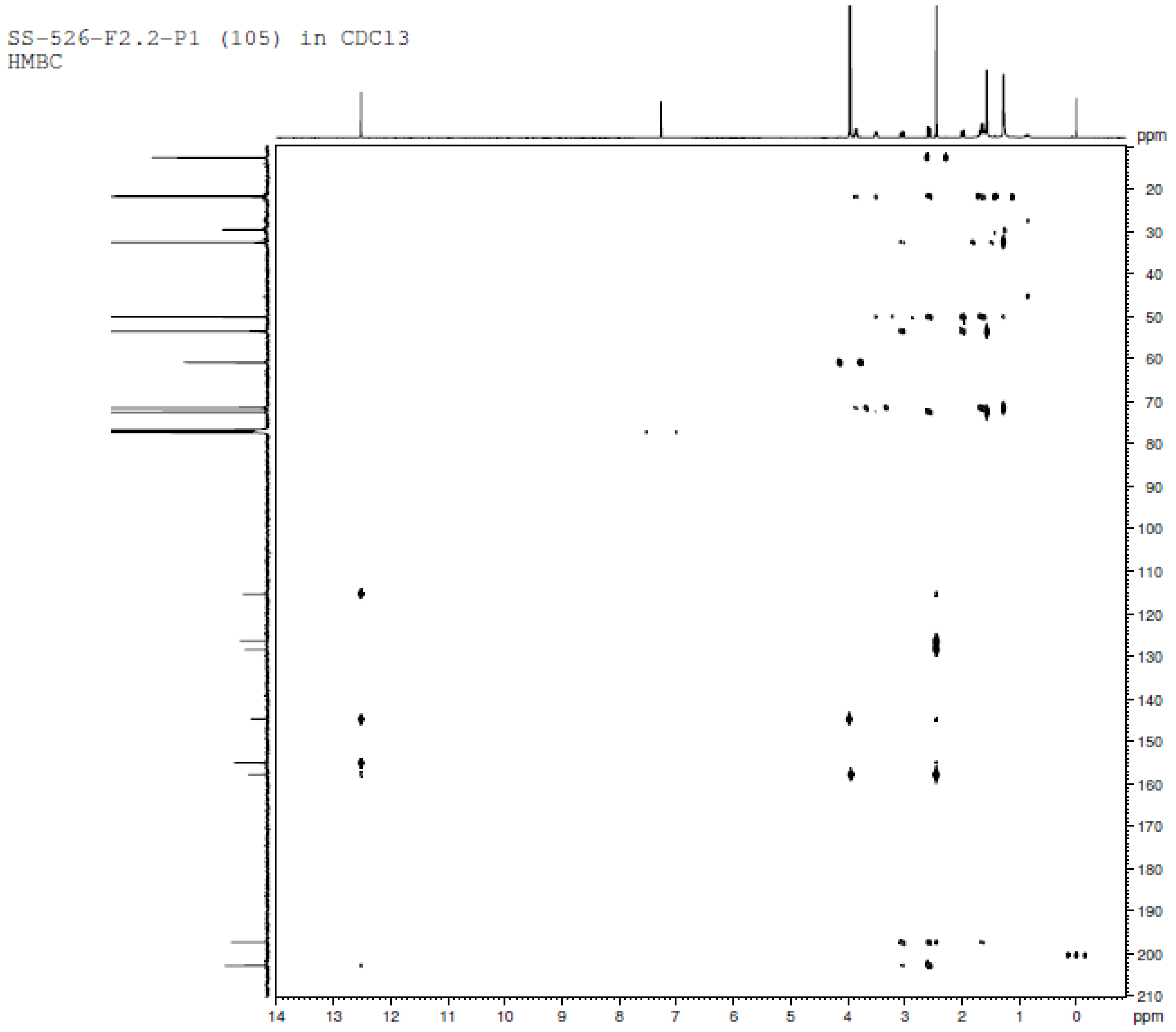

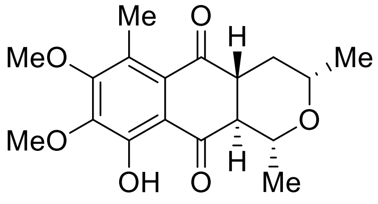

3

Figure S19 NOESY spectrum of compound **3**

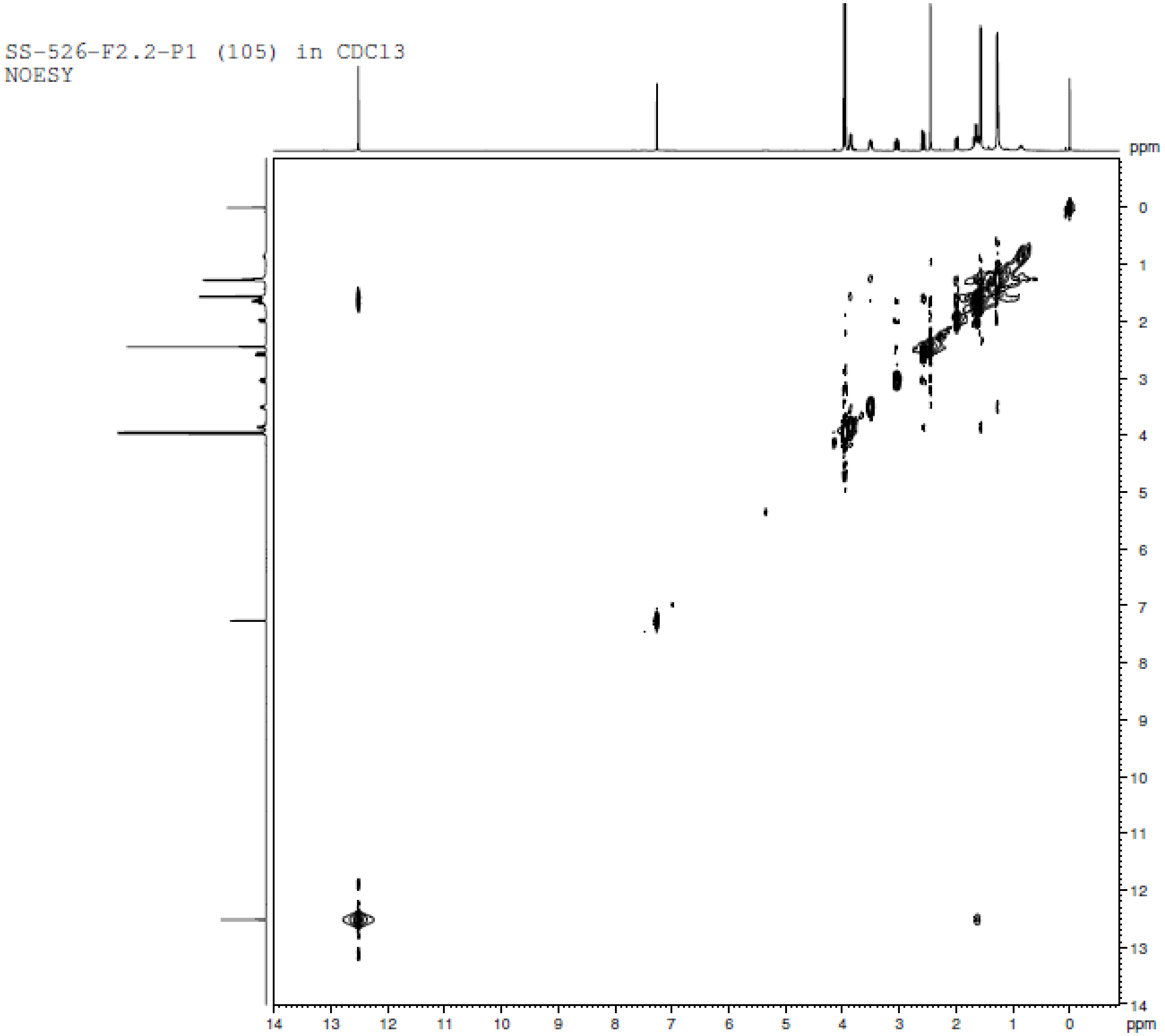

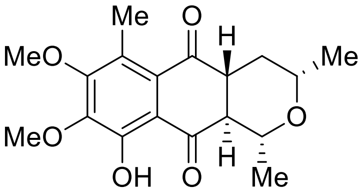

3

Figure S20 ^{1}H NMR (400 MHz) spectrum of compound **4** in $CDCl_3$

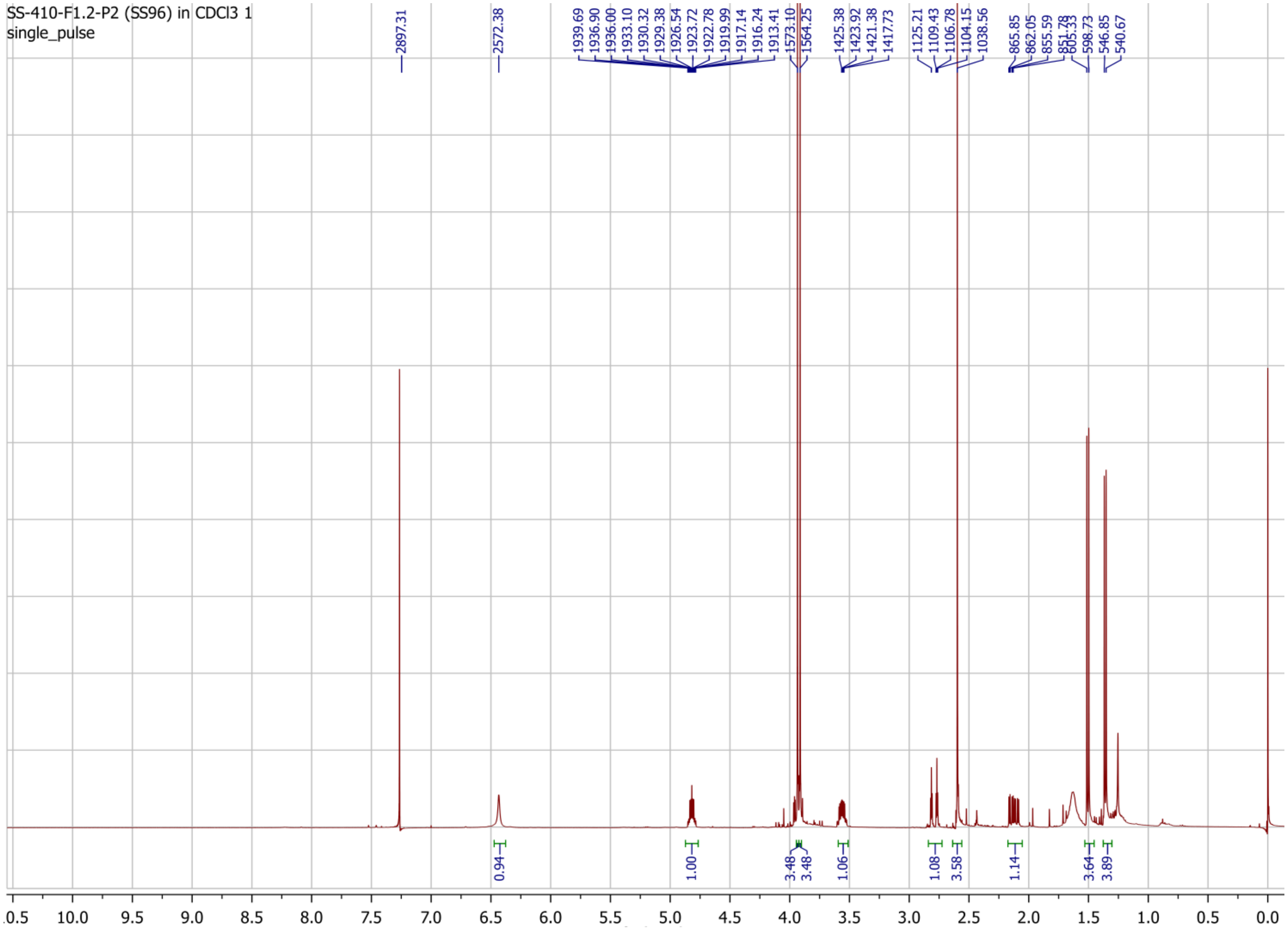

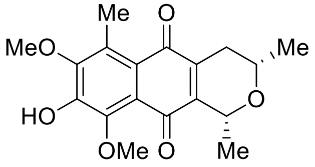

4

Figure S21 ^{13}C NMR (400 MHz) spectrum of compound **4** in $CDCl_3$

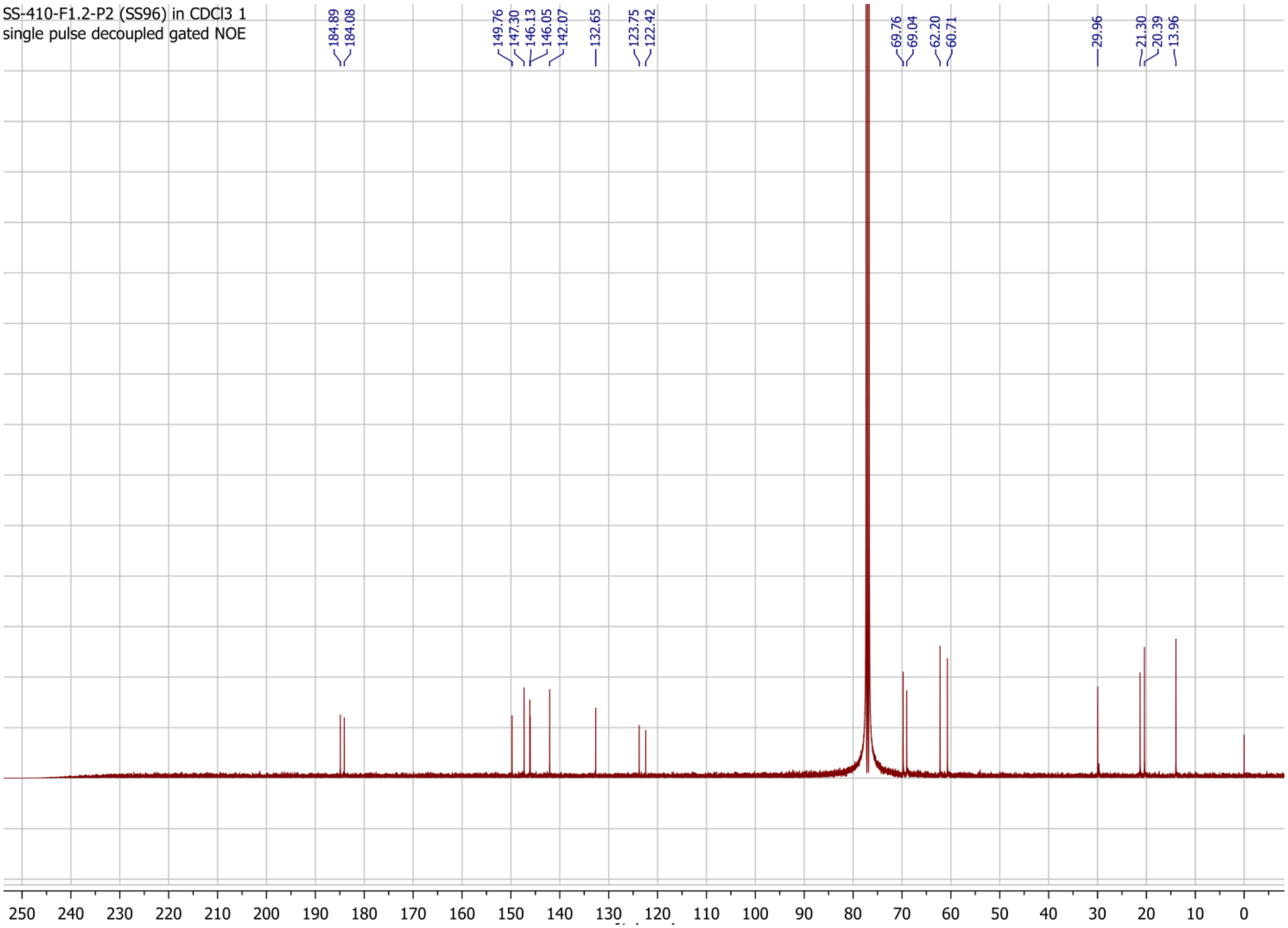

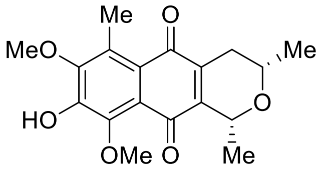

4

Figure S22 COSY spectrum of compound **4**

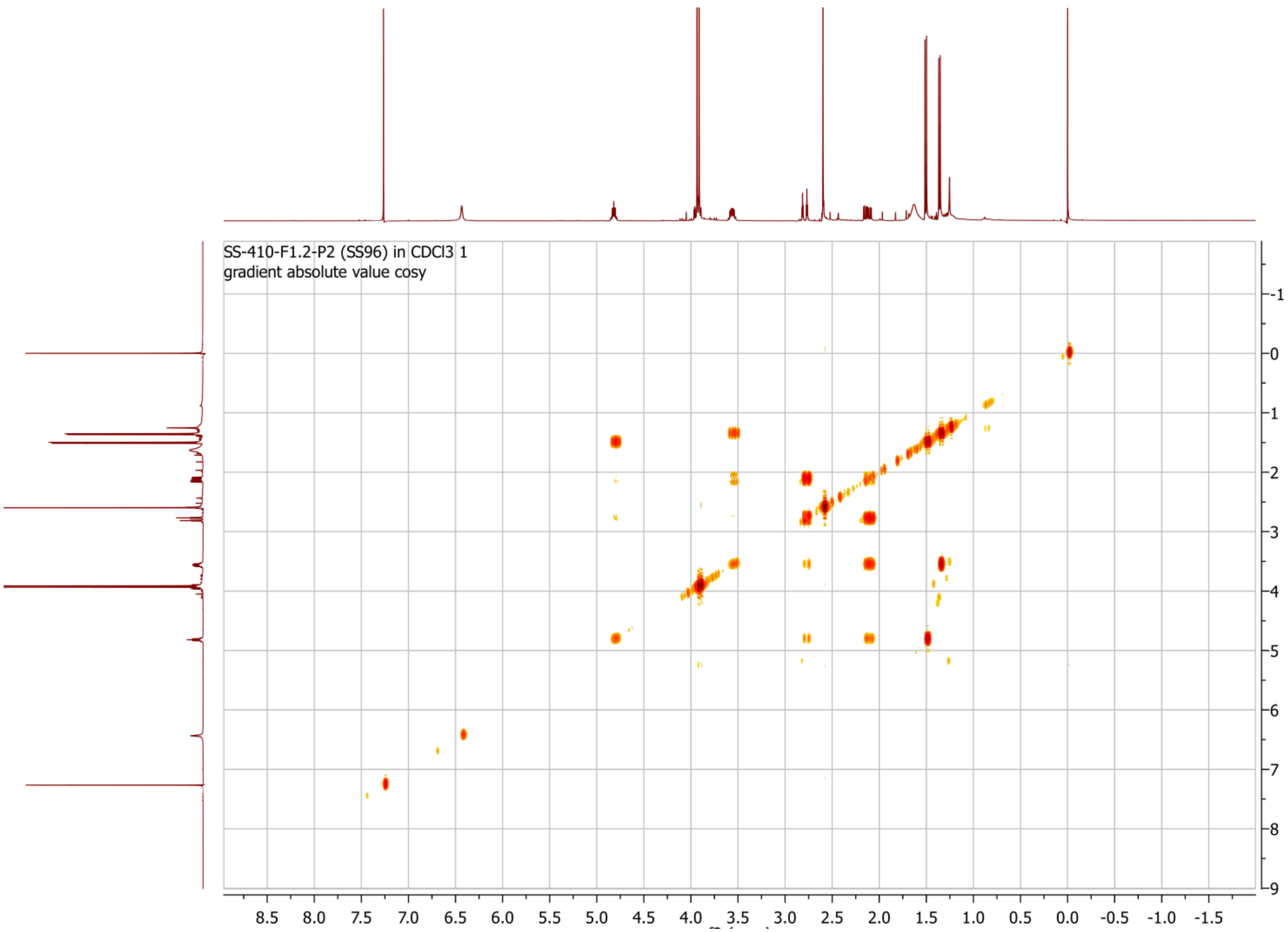

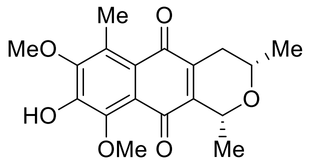

4

Figure S23 HMQC spectrum of compound **4**

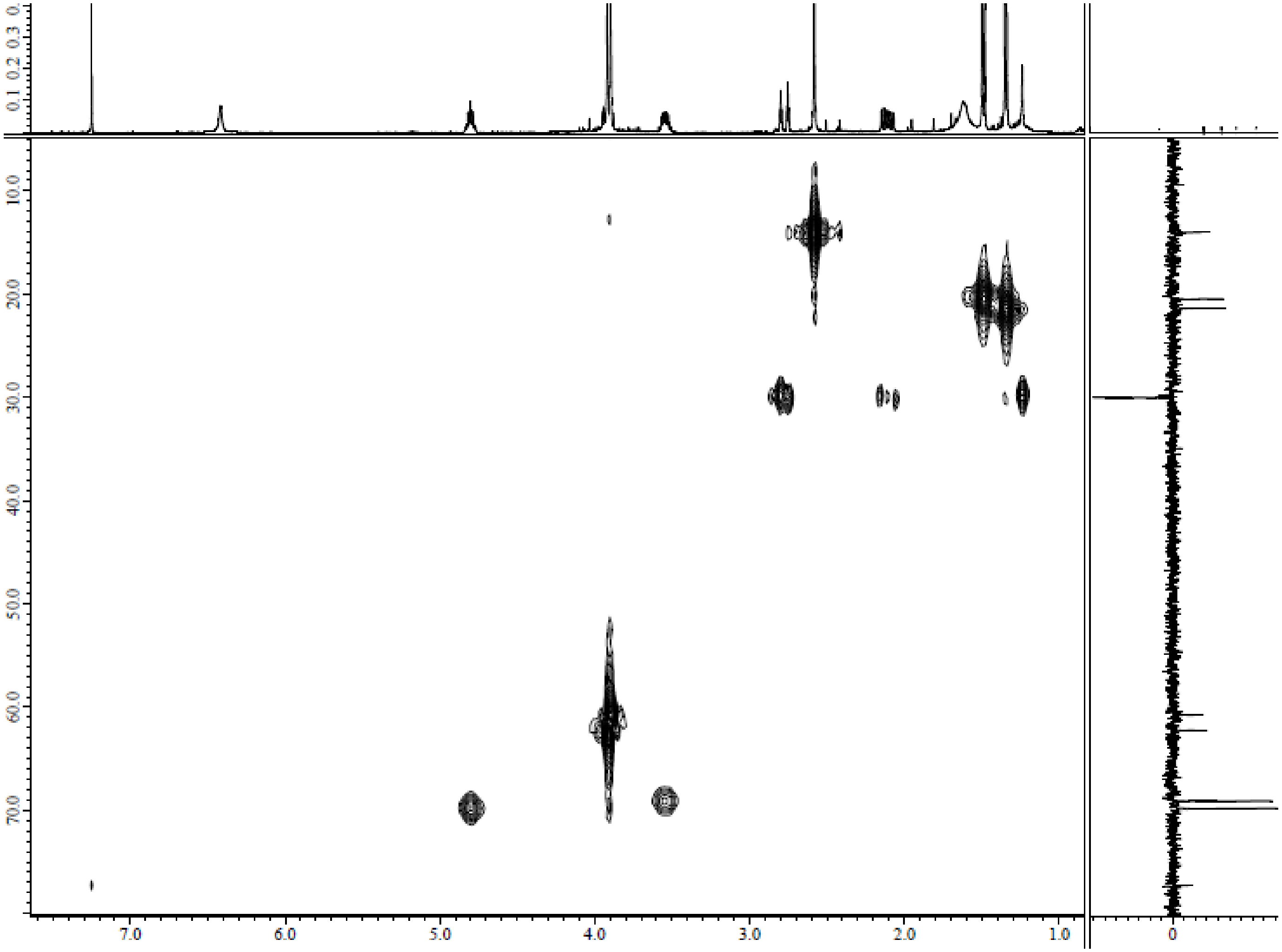

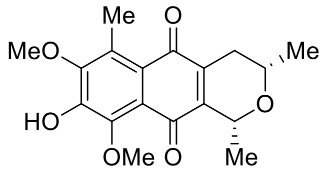

4

Figure S24 HMBC spectrum of compound **4**

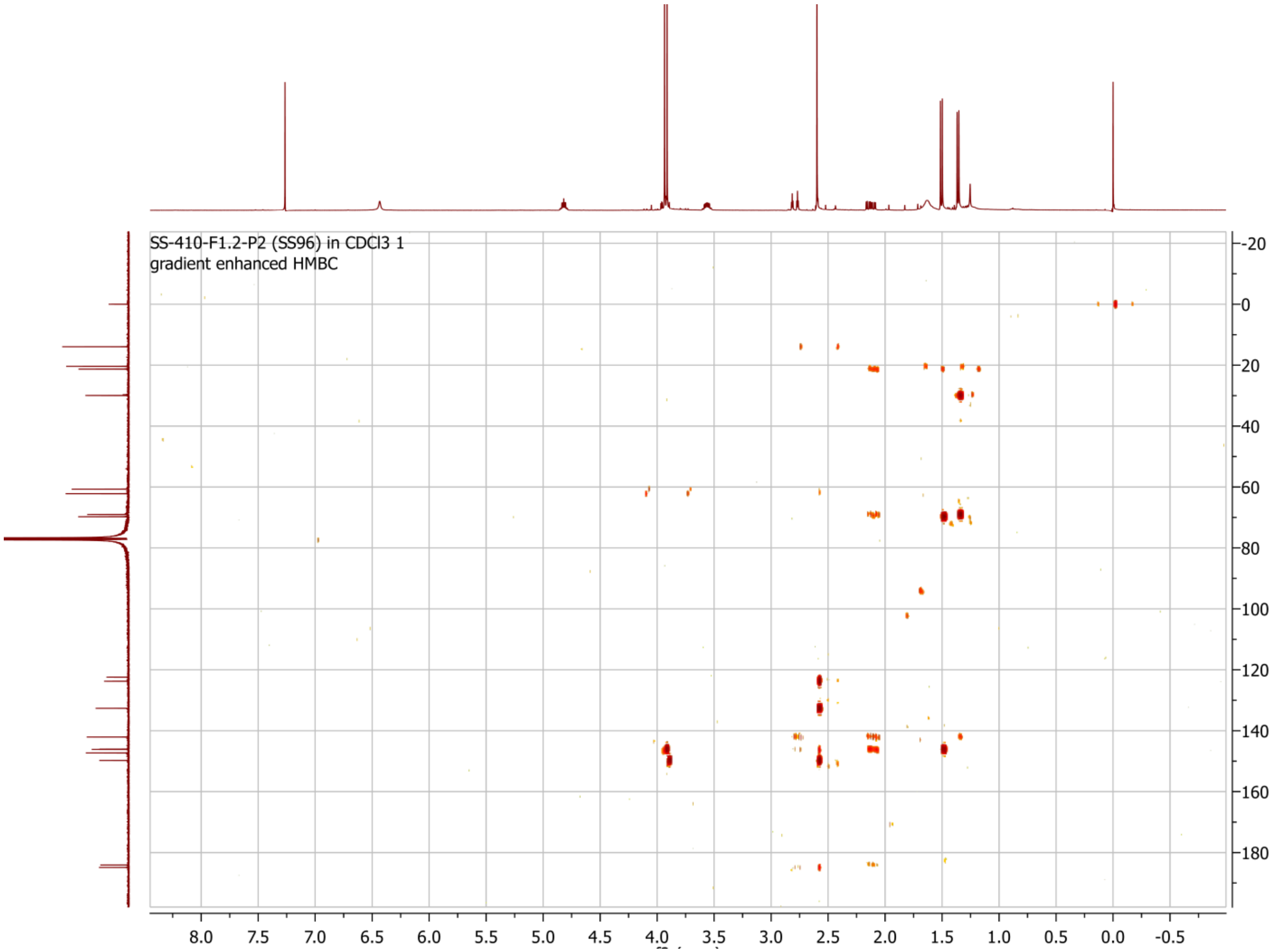

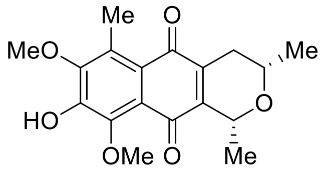

4

Figure S25 NOESY spectrum of compound **4**

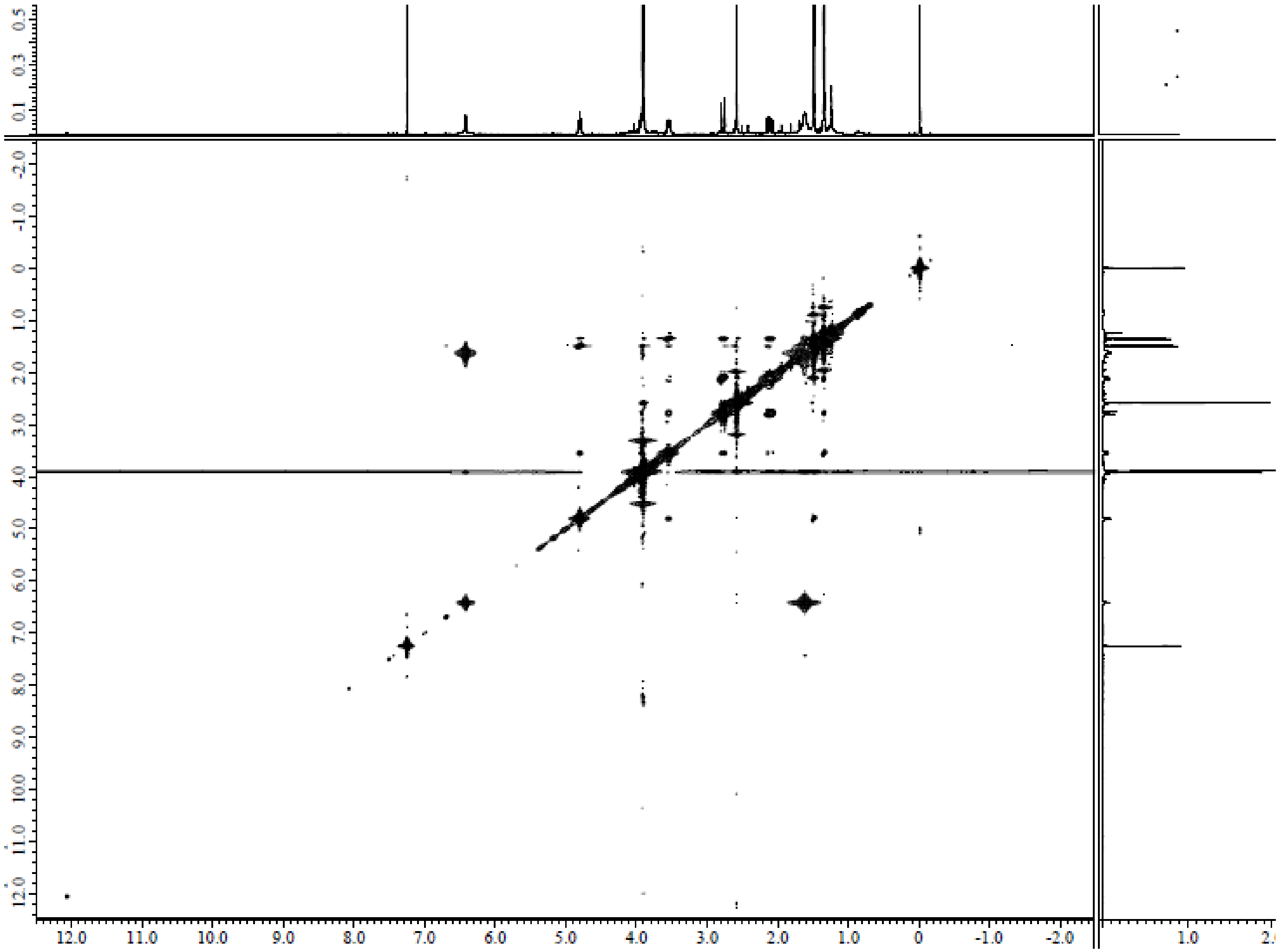

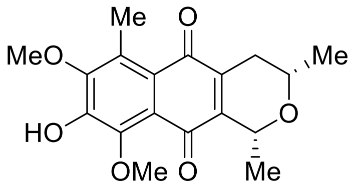

4

Figure S26 ^{1}H NMR (400 MHz) spectrum of compound **5** in $CDCl_3$

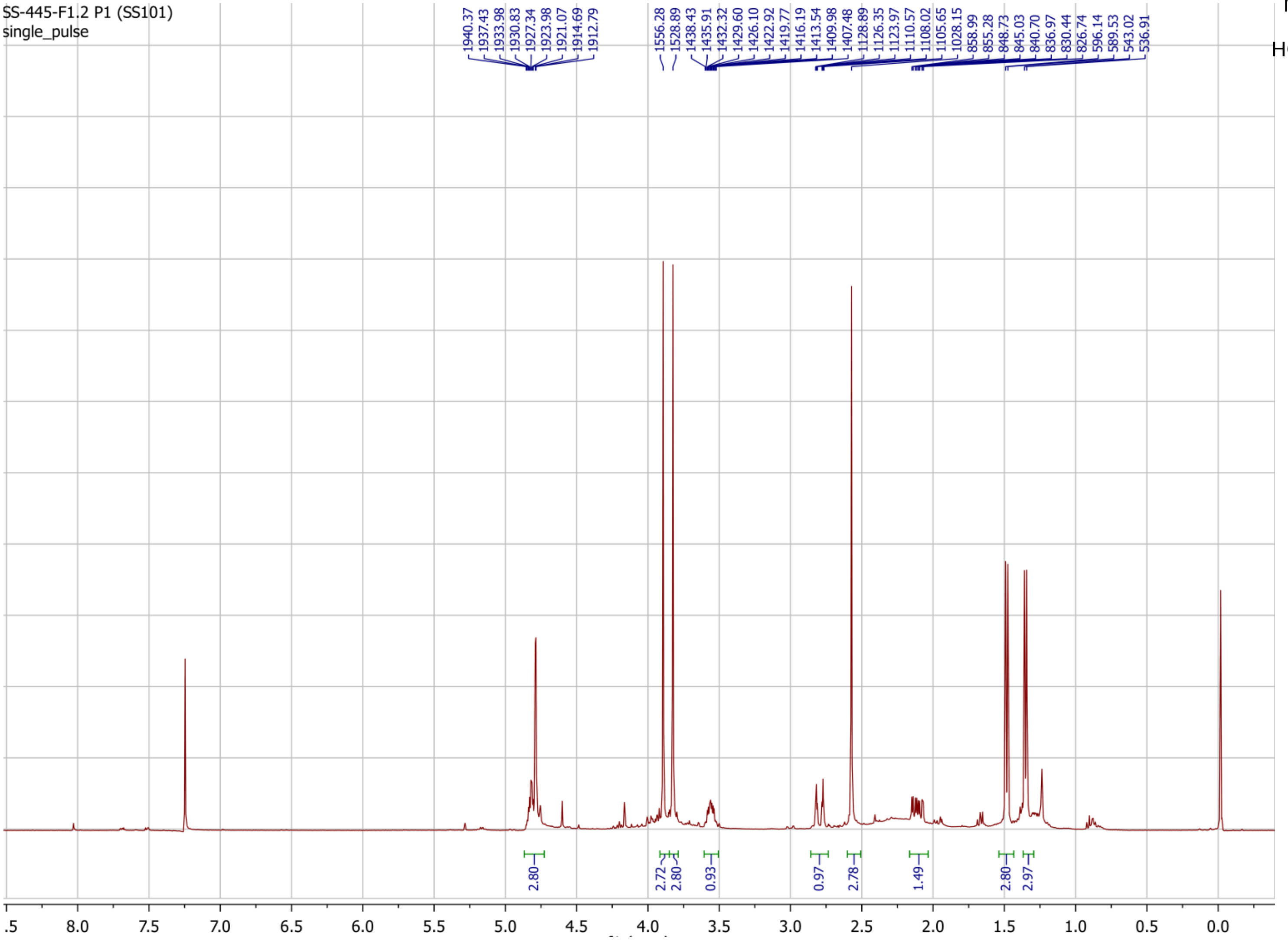

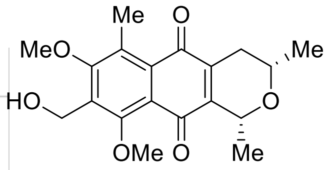

5

Figure S27 ^{13}C NMR (400 MHz) spectrum of compound **5** in $CDCl_3$

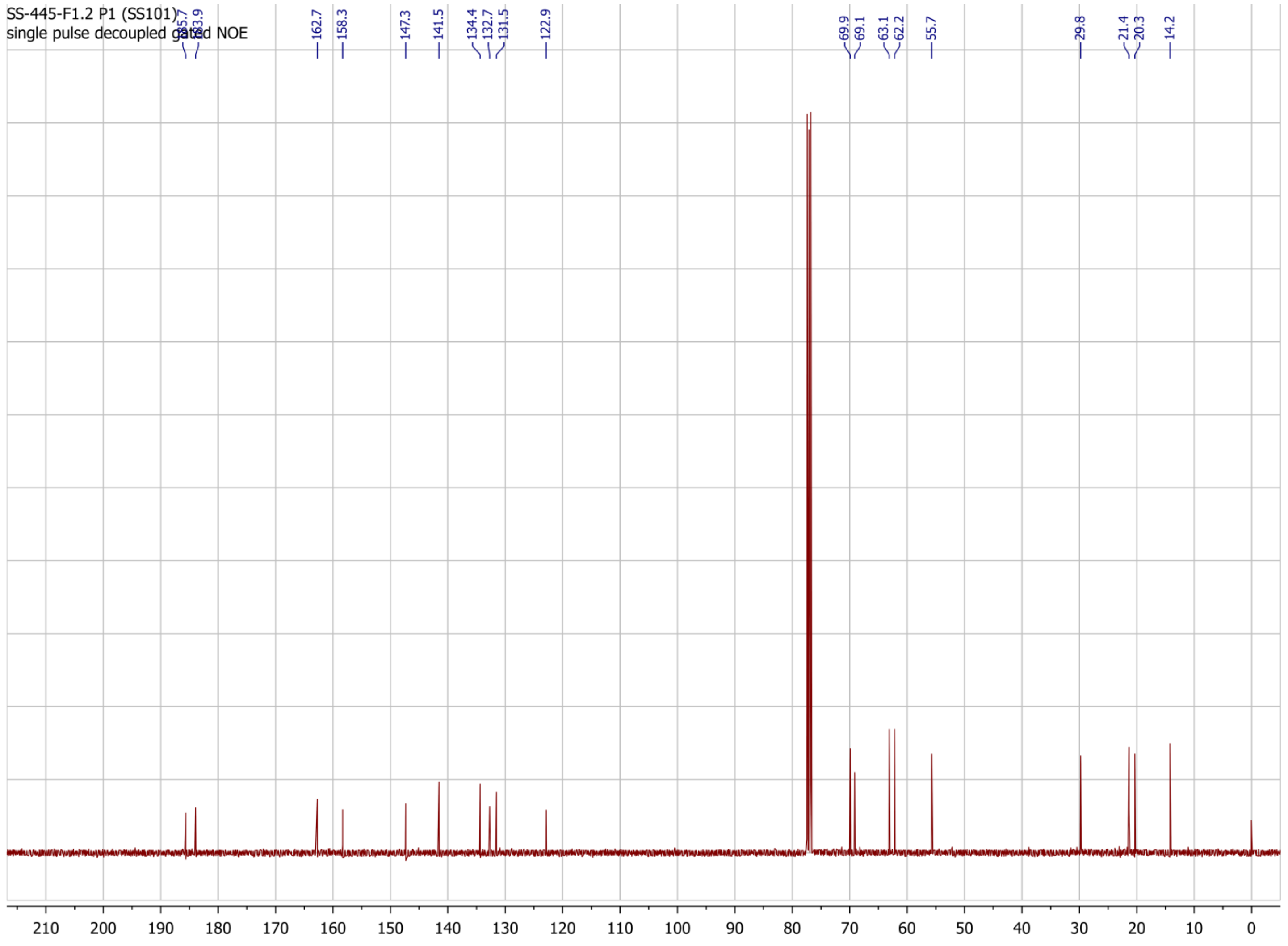

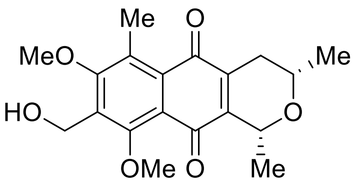

5

Figure S28 COSY spectrum of compound **5**

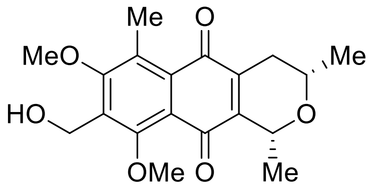

5

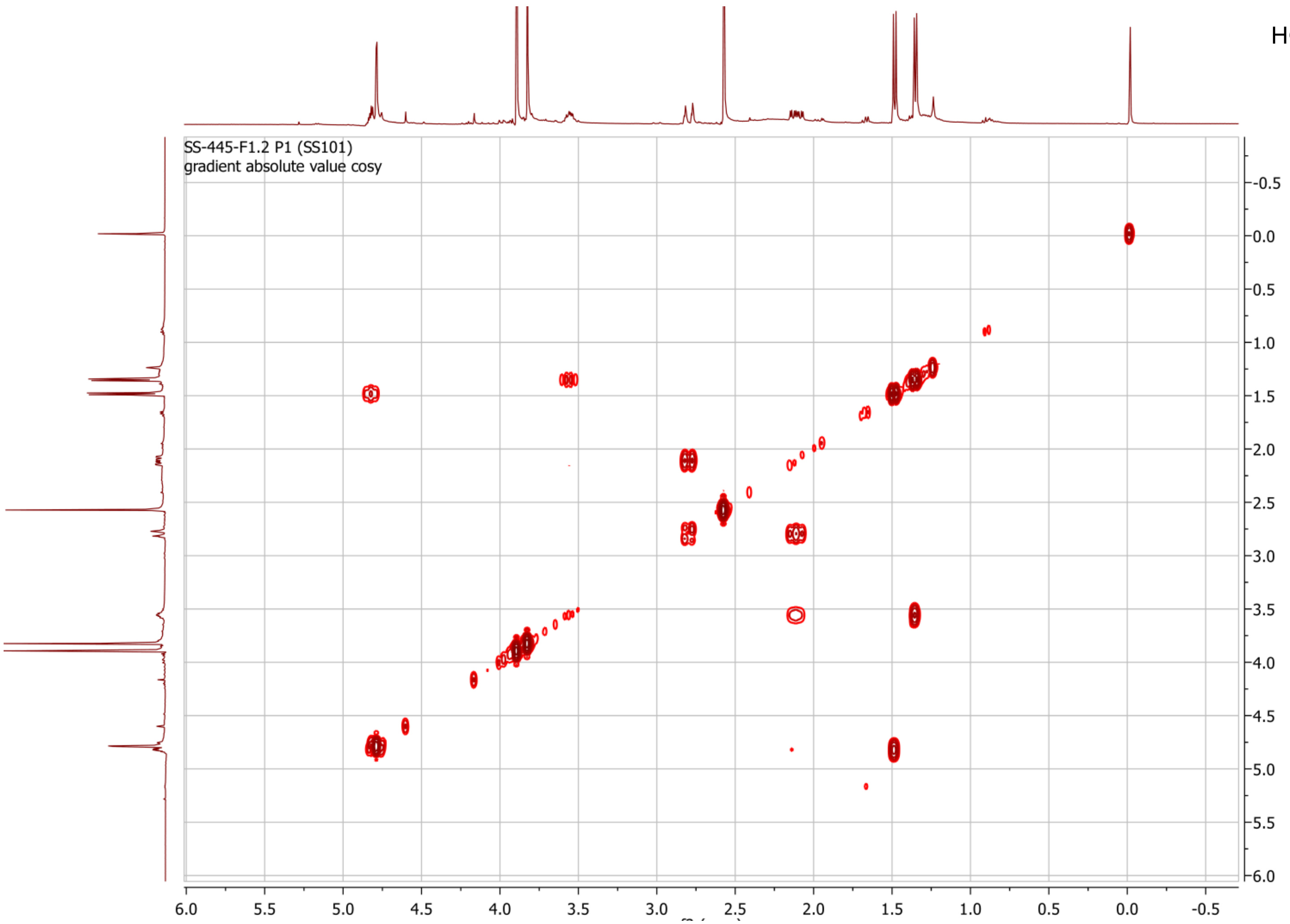

Figure S29 HMQC spectrum of compound **5**

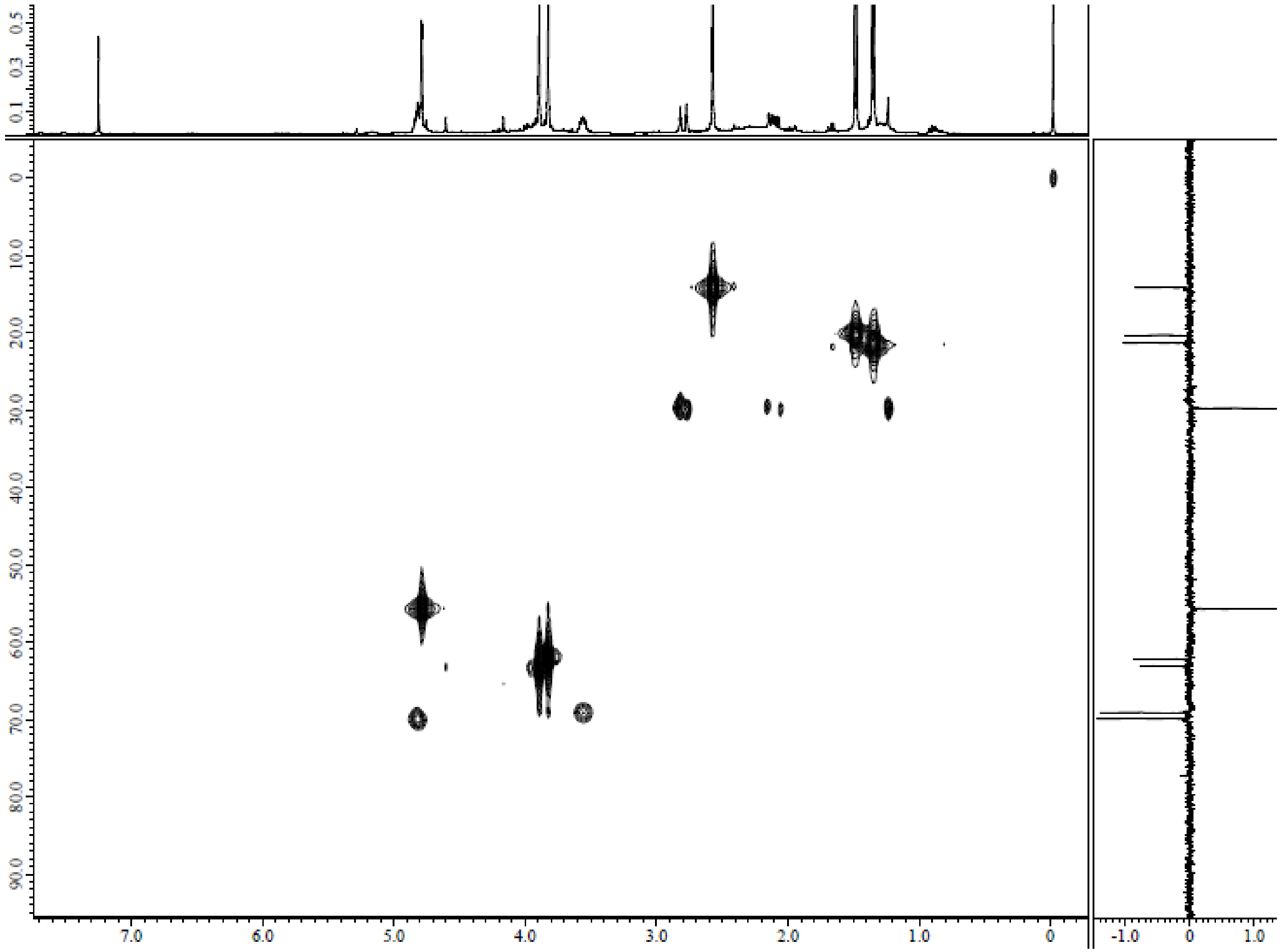

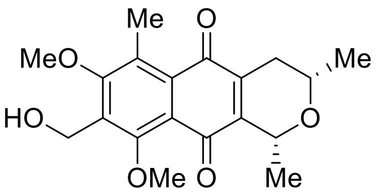

5

Figure S30 HMBC spectrum of compound **5**

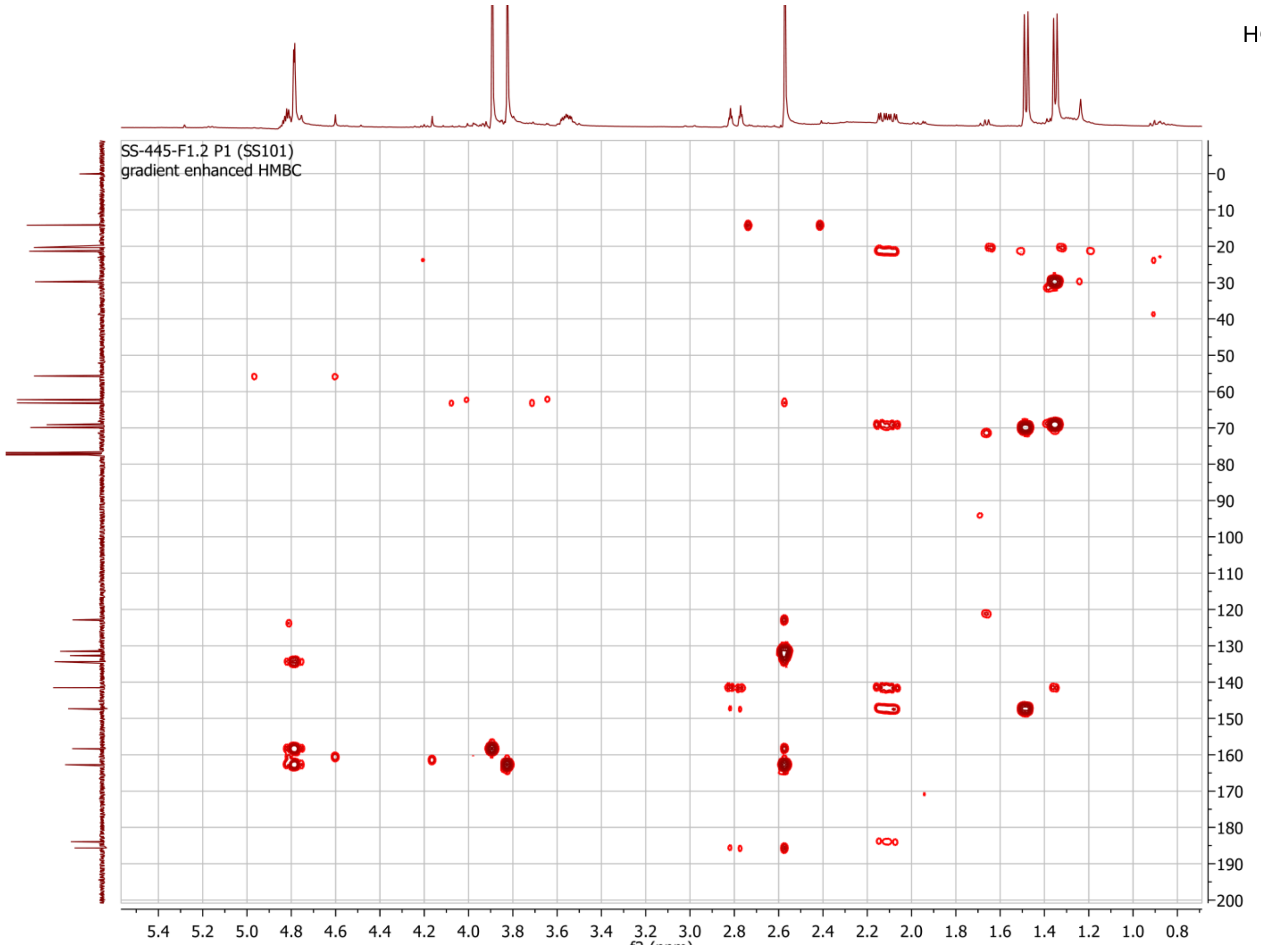

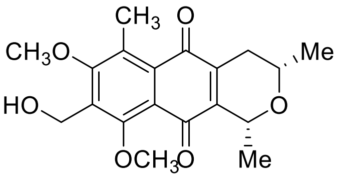

5

Figure S31 NOESY spectrum of compound **5**

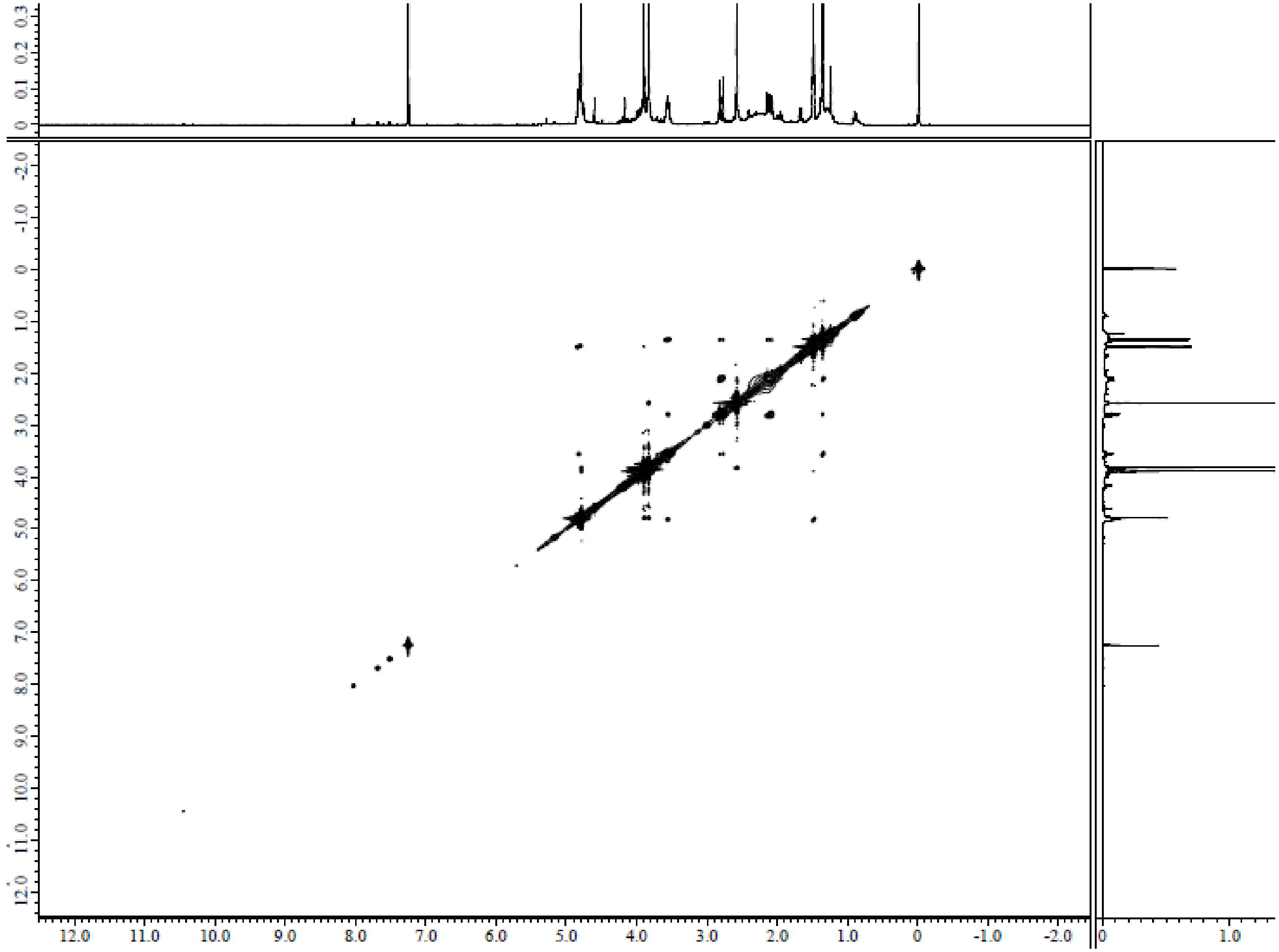

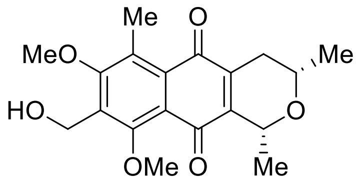

5

Figure S32 ^{1}H NMR (400 MHz) spectrum of compound **6** in $CDCl_3$

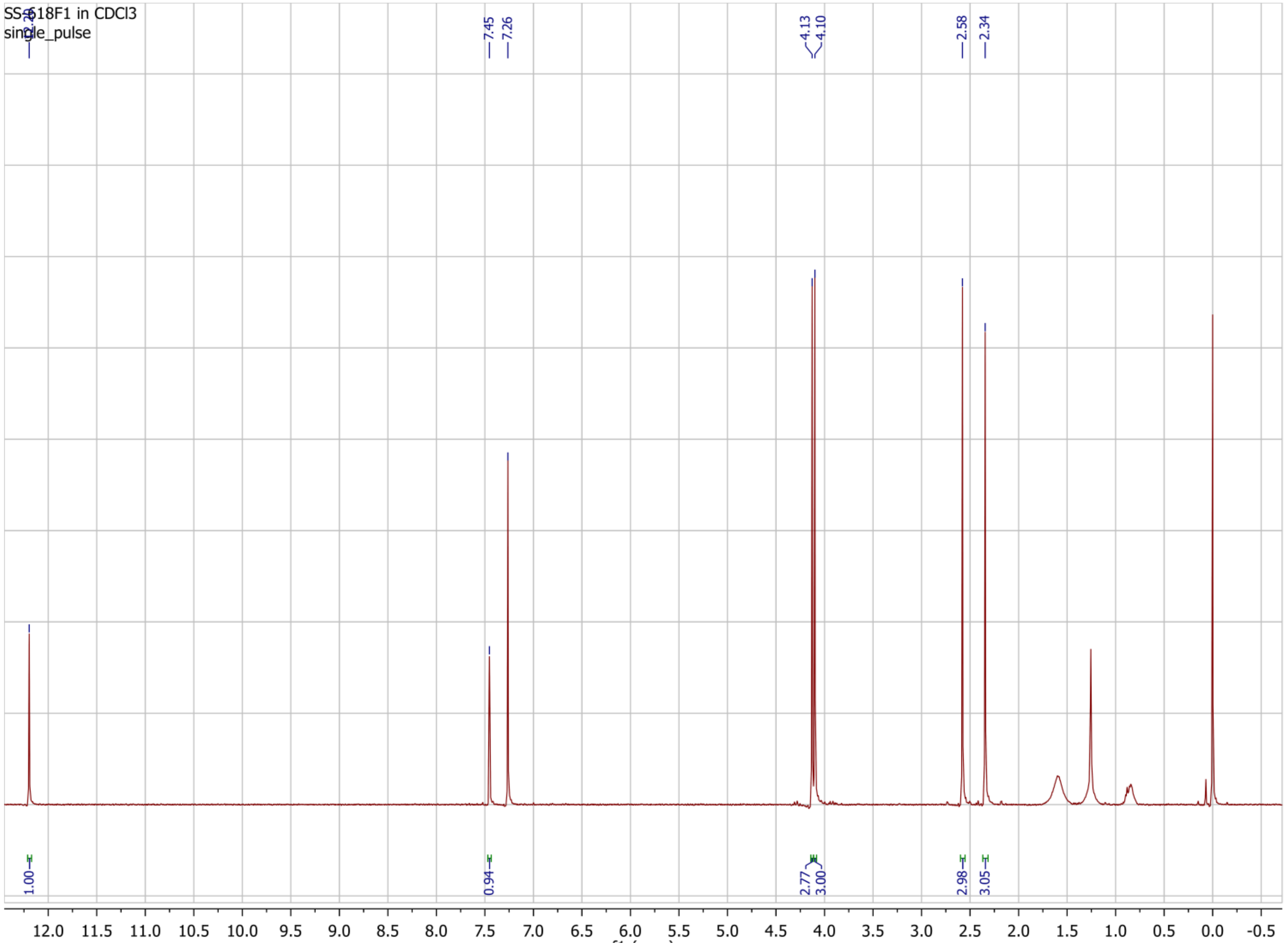

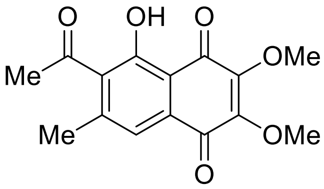

6

Figure S33 ^{13}C NMR (400 MHz) spectrum of compound **6** in $CDCl_3$

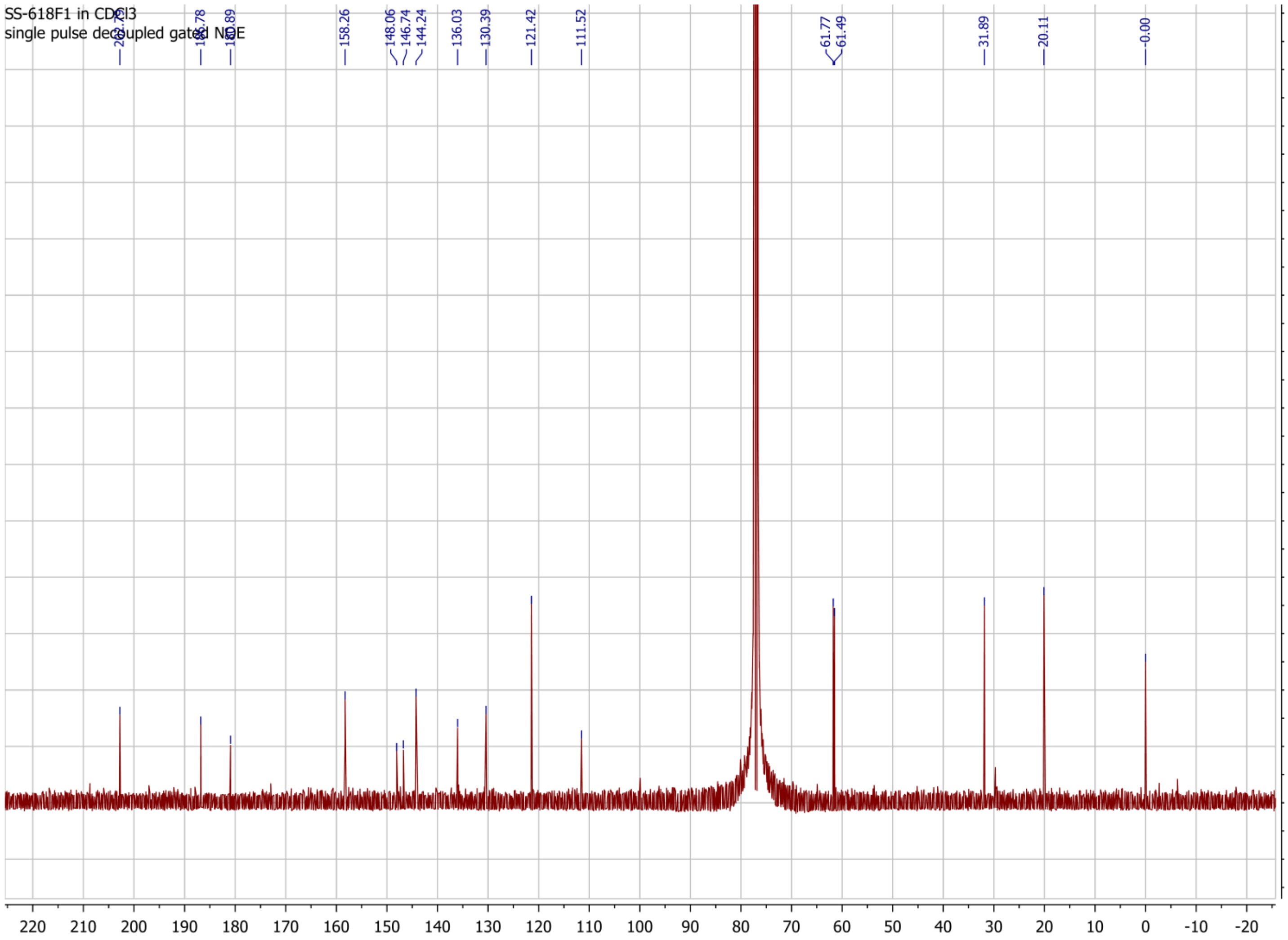

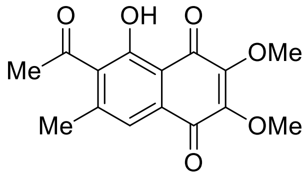

6

Figure S34 COSY spectrum of compound **6**

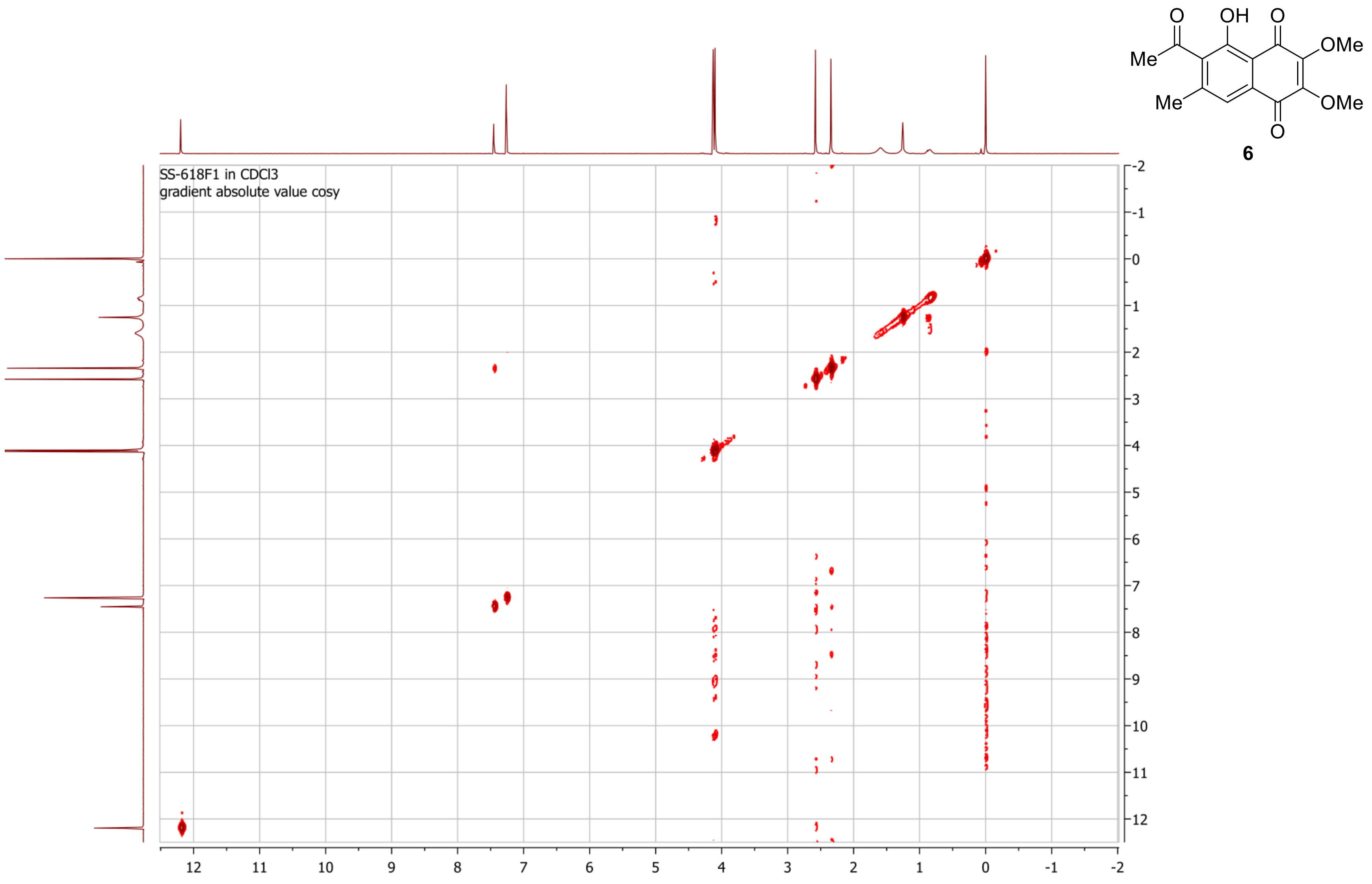

Figure S35 HMQC spectrum of compound **6**

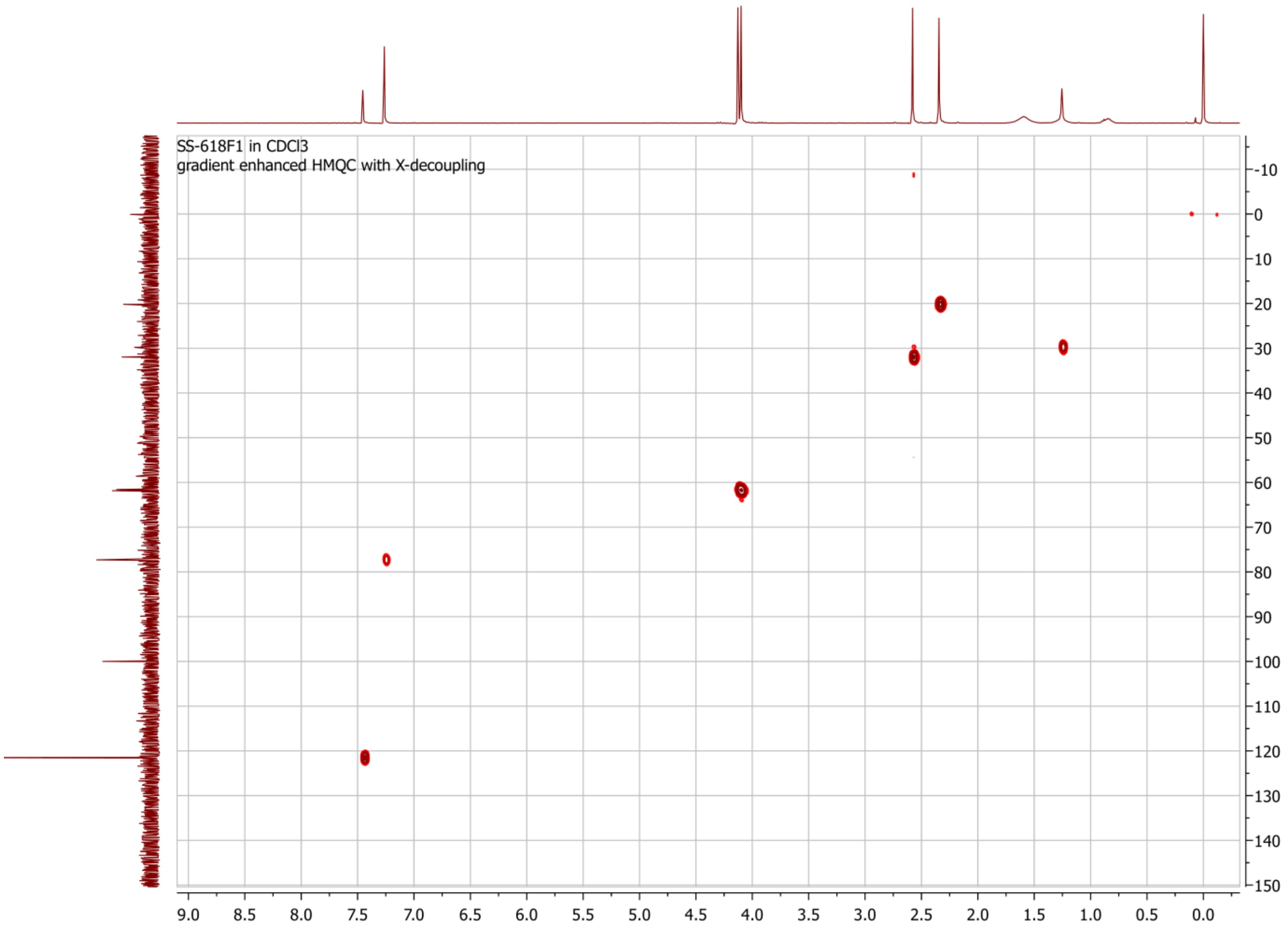

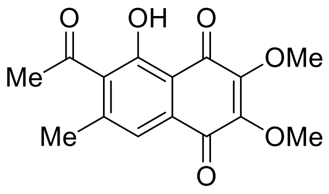

6

Figure S36 HMBC spectrum of compound **6**

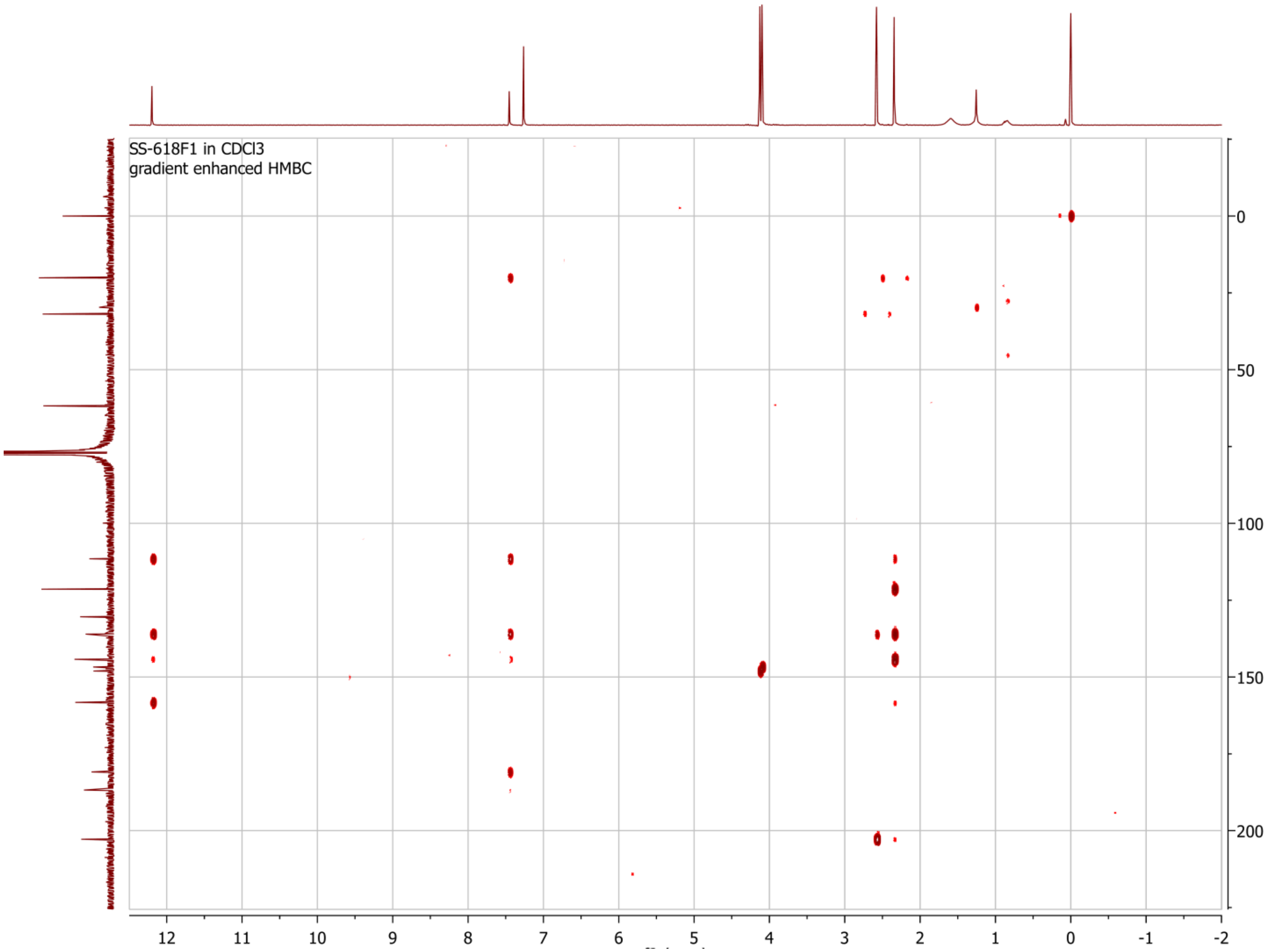

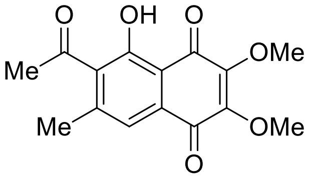

6

Figure S37 NOESY spectrum of compound **6**

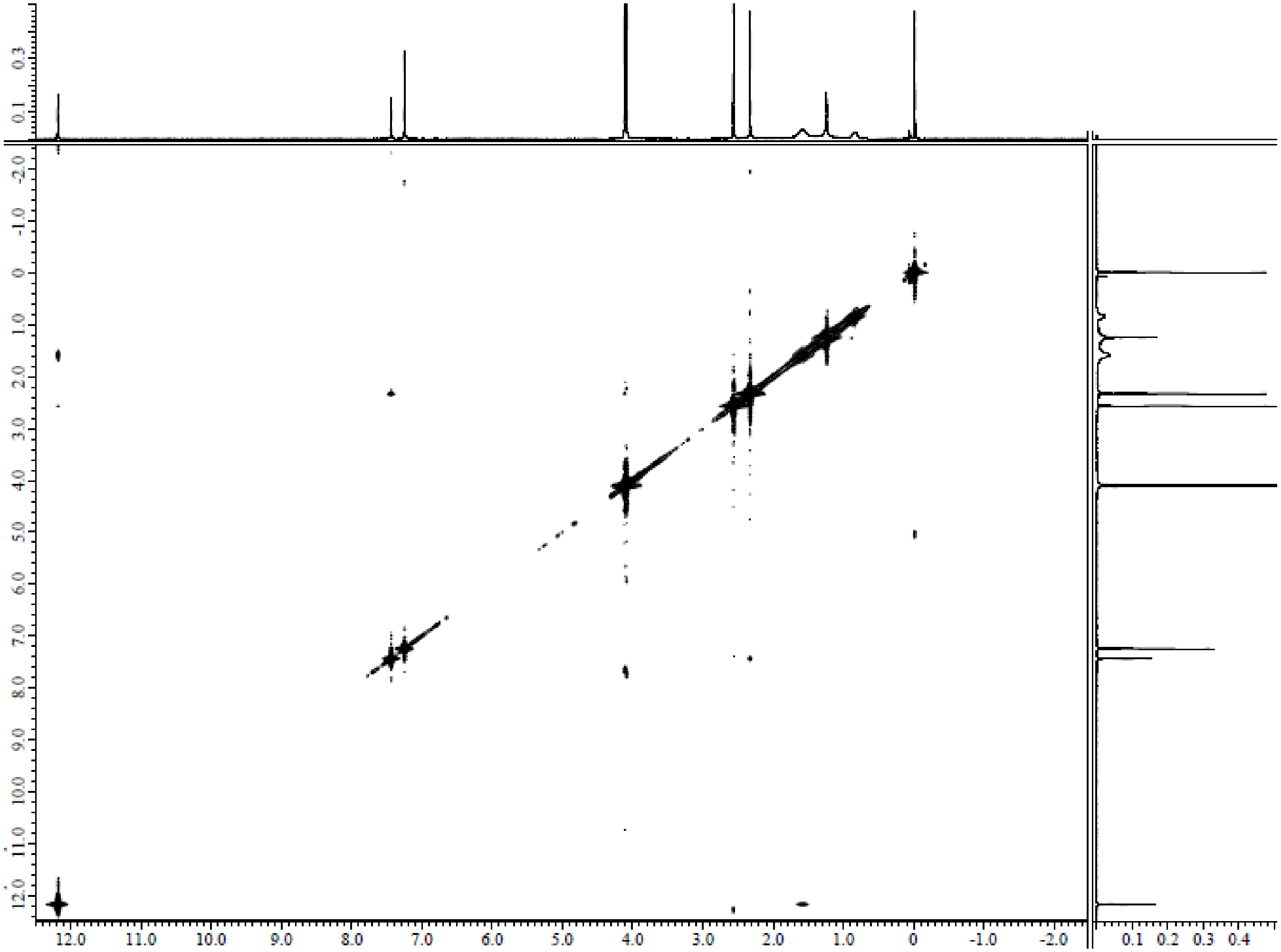

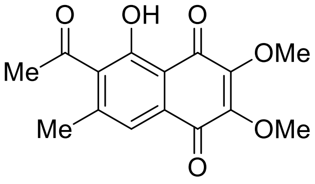

6

Figure S38 ^{1}H NMR (400 MHz) spectrum of compound **7** in $CDCl_3$

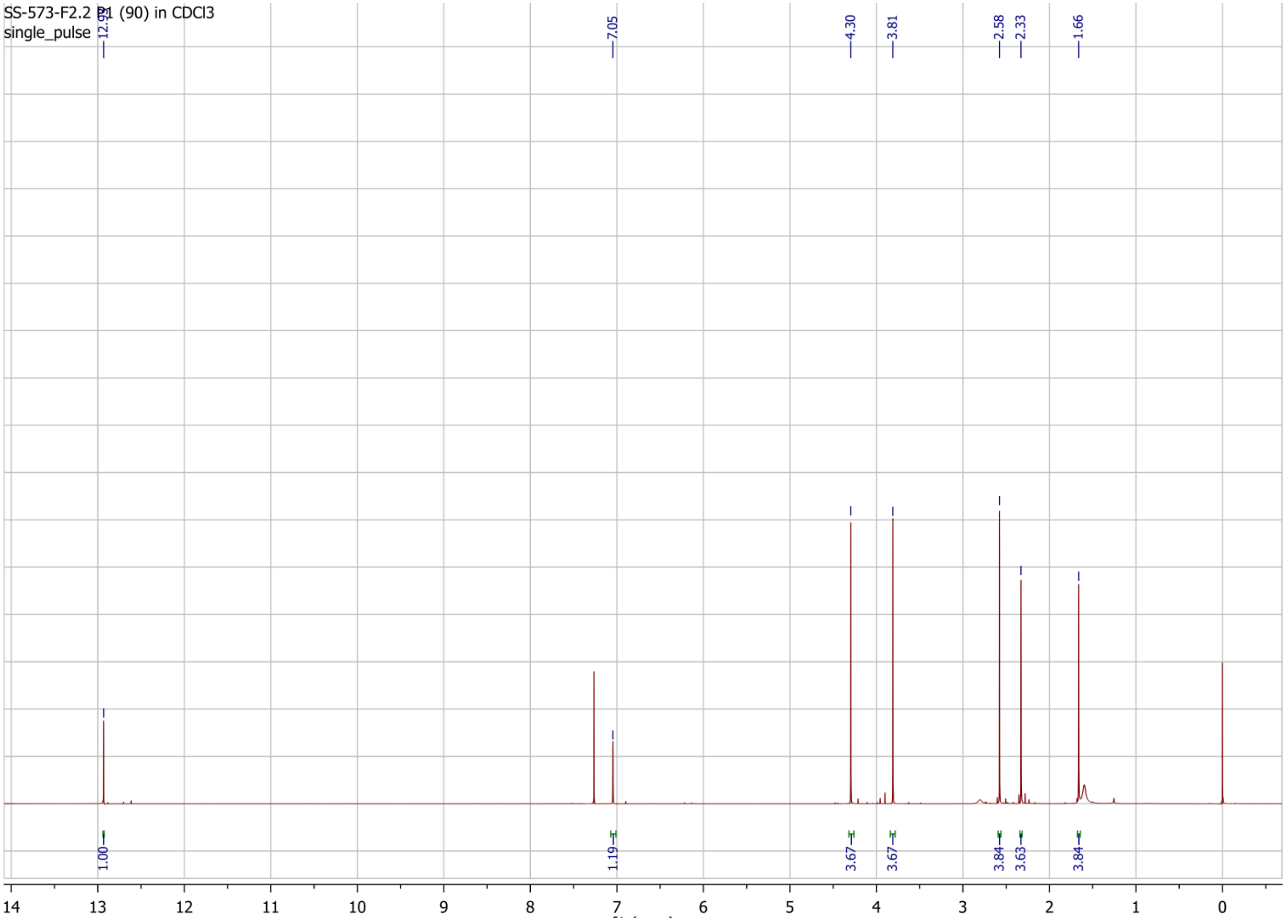

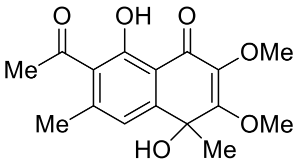

7

Figure S39 ^{13}C NMR (400 MHz) spectrum of compound **7** in $CDCl_3$

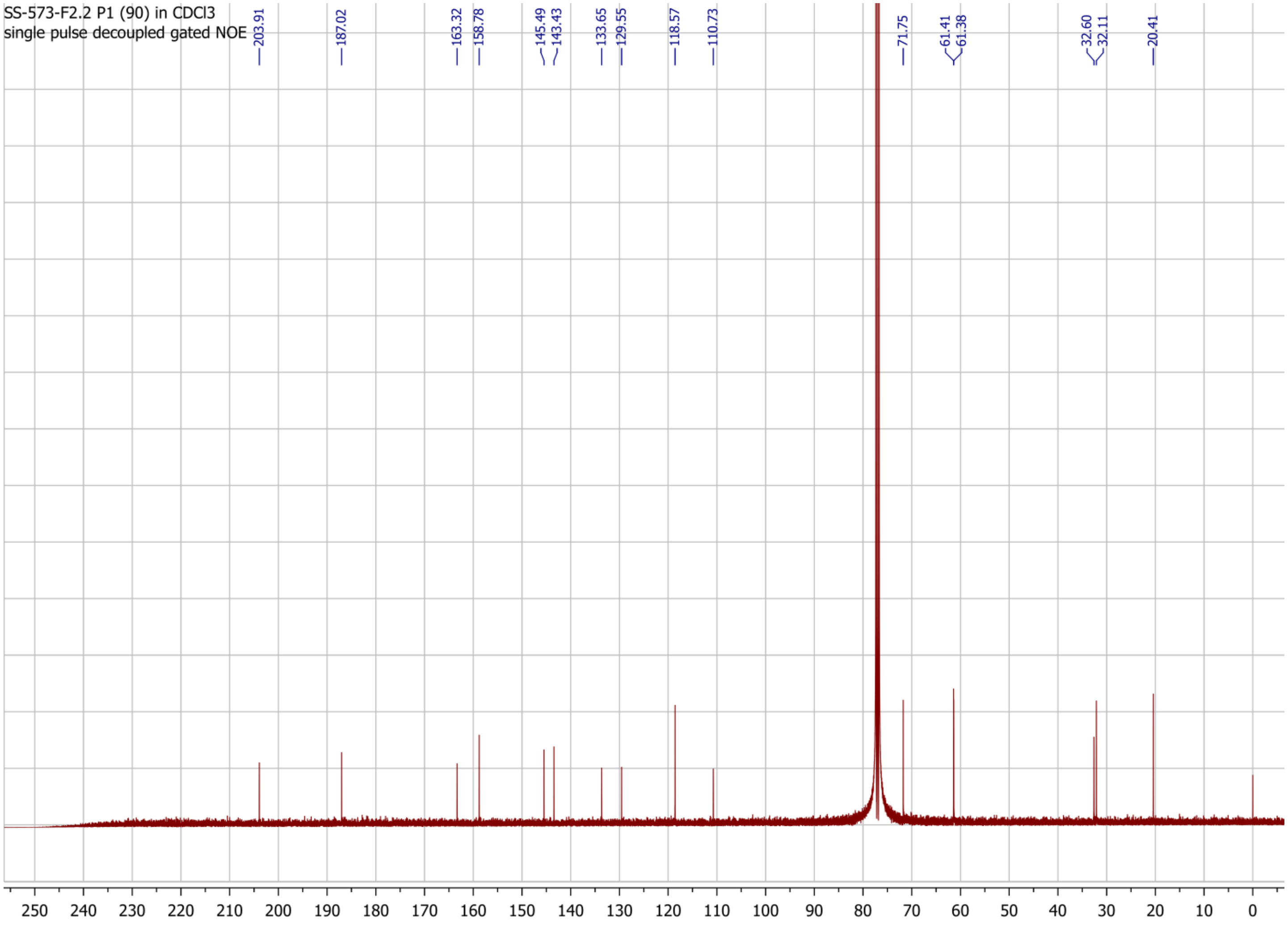

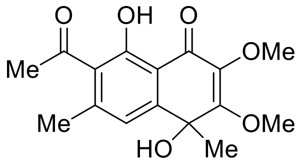

7

Figure S40 COSY spectrum of compound **7**

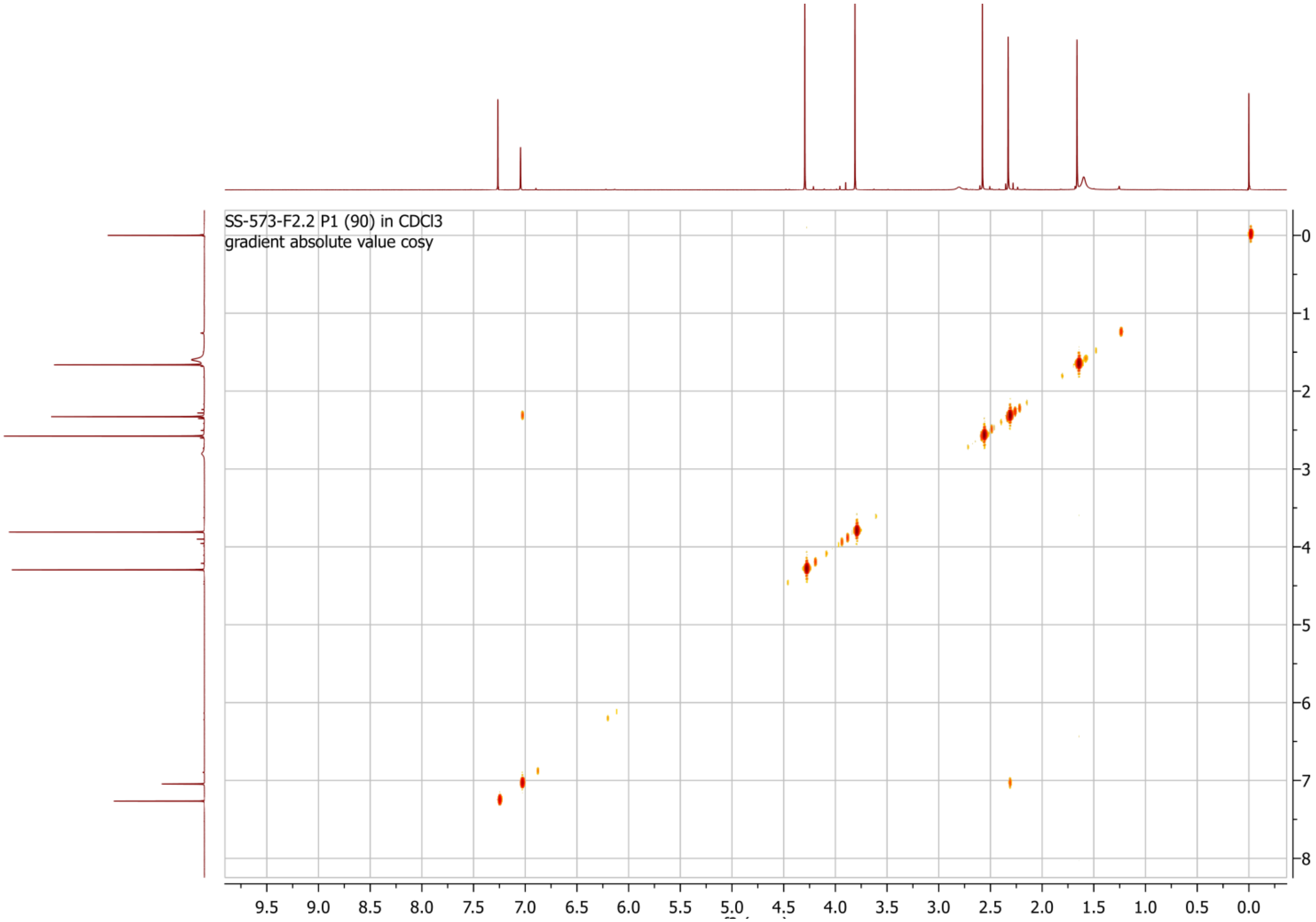

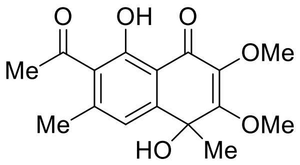

7

Figure S41 HMQC spectrum of compound **7**

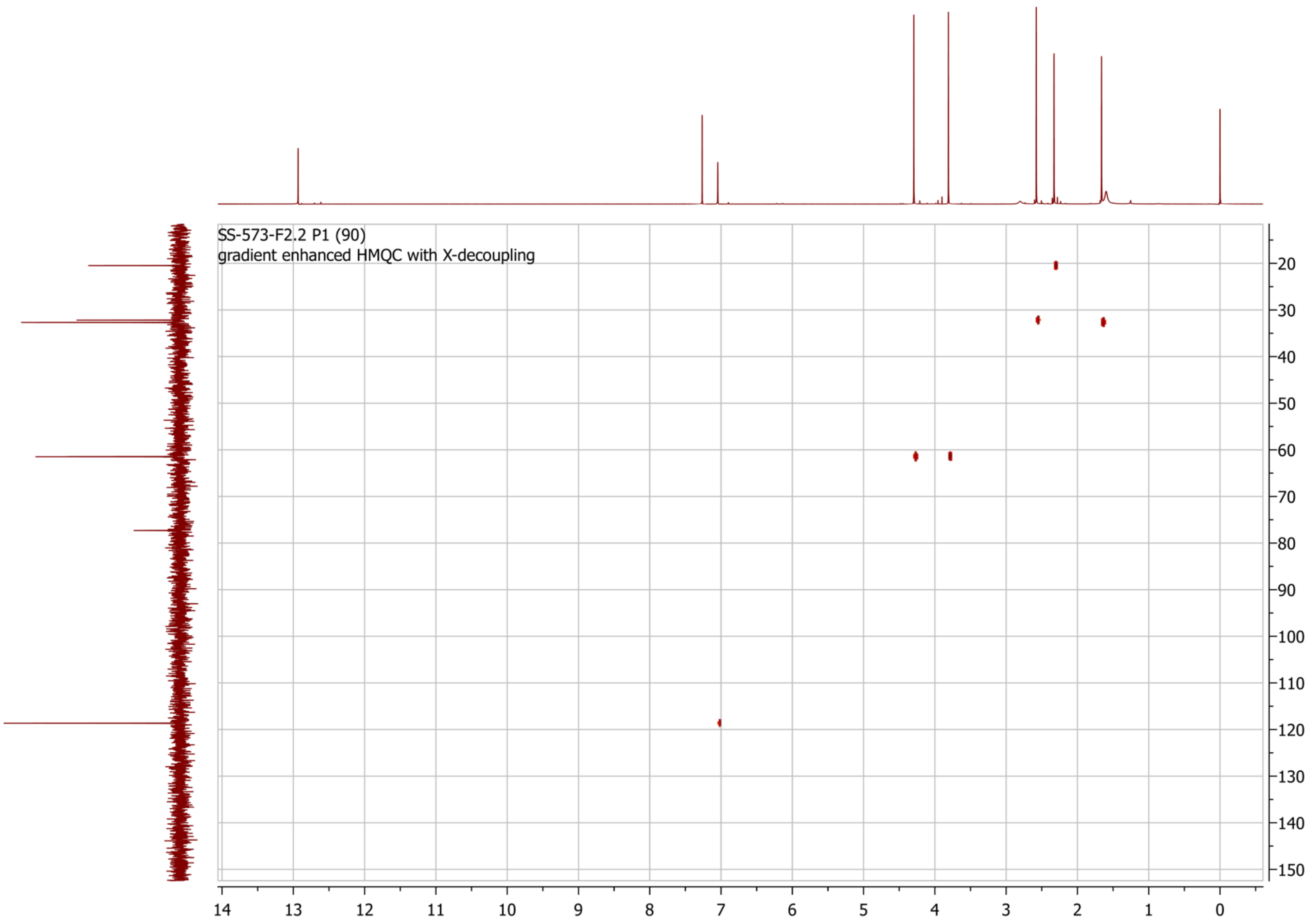

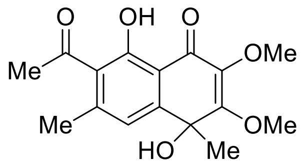

7

Figure S42 HMBC spectrum of compound **7**

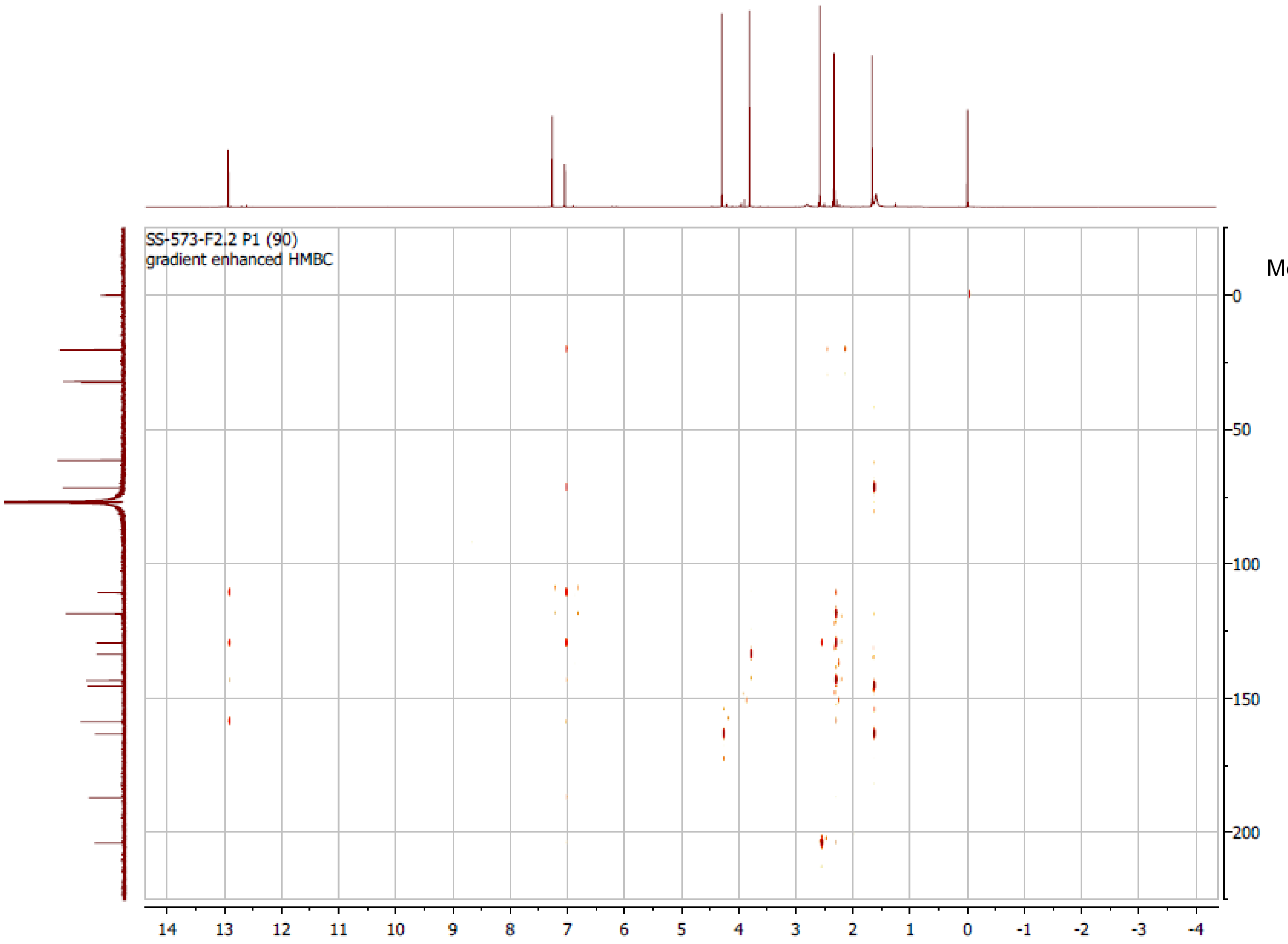

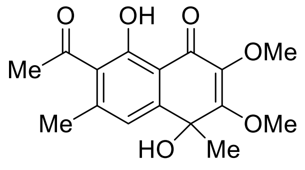

7

Figure S43 NOESY spectrum of compound **7**

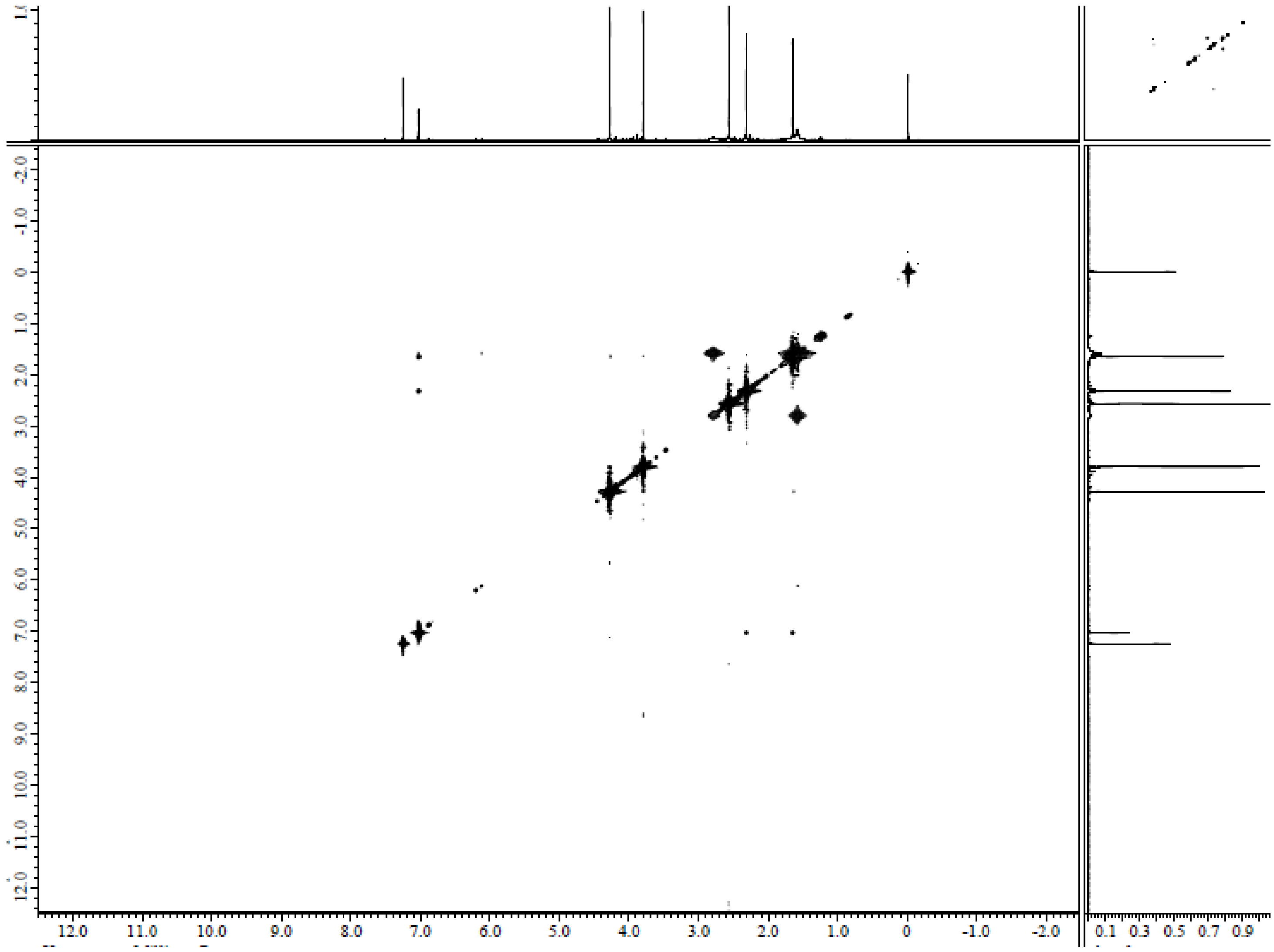

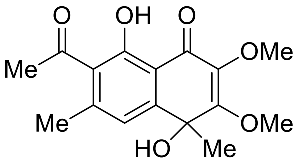

7

Figure S44 ^{1}H NMR (400 MHz) spectrum of compound **16** in $CDCl_3$

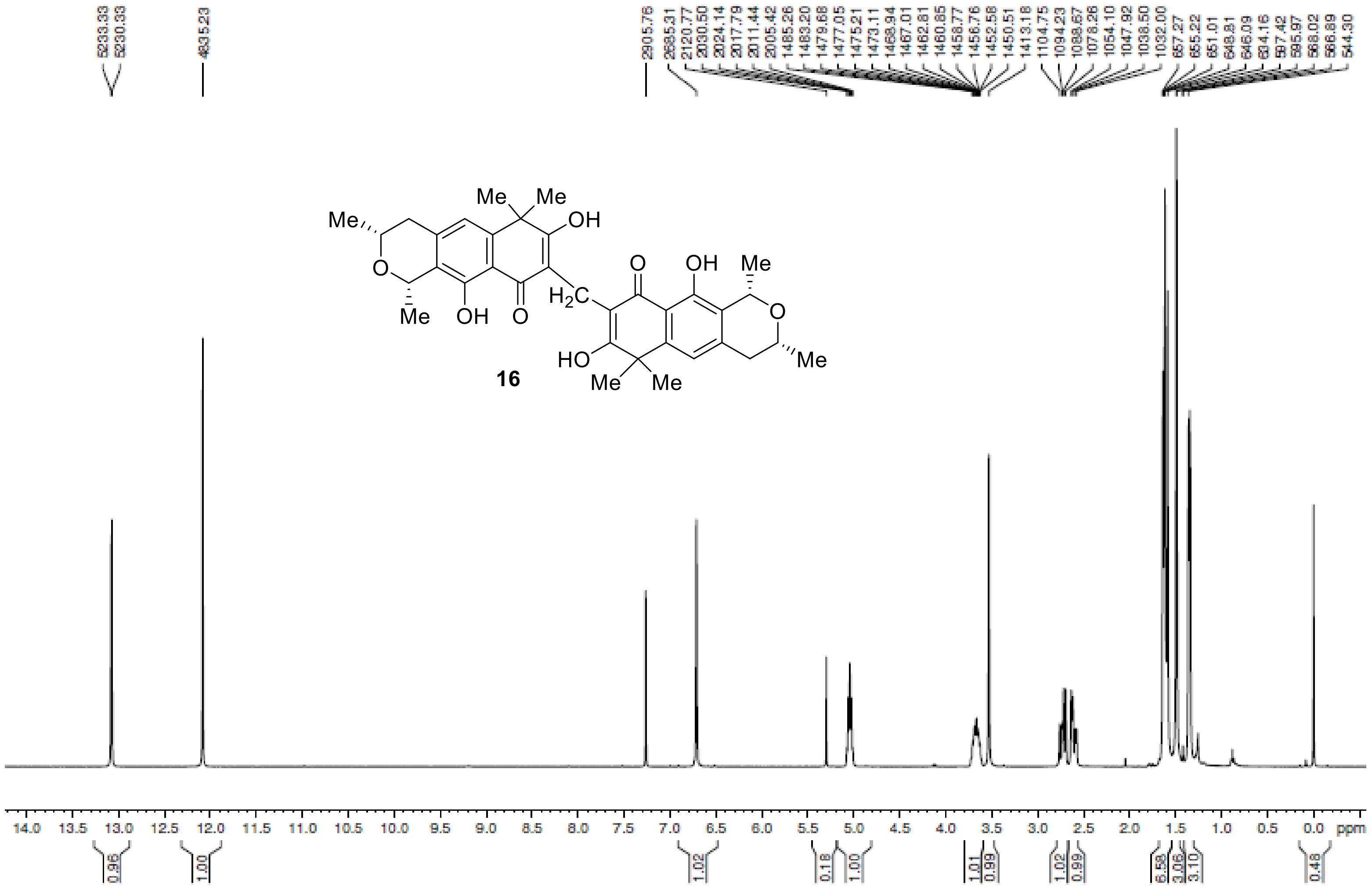

Figure S45 ^{13}C NMR (400 MHz) spectrum of compound **16** in $CDCl_3$

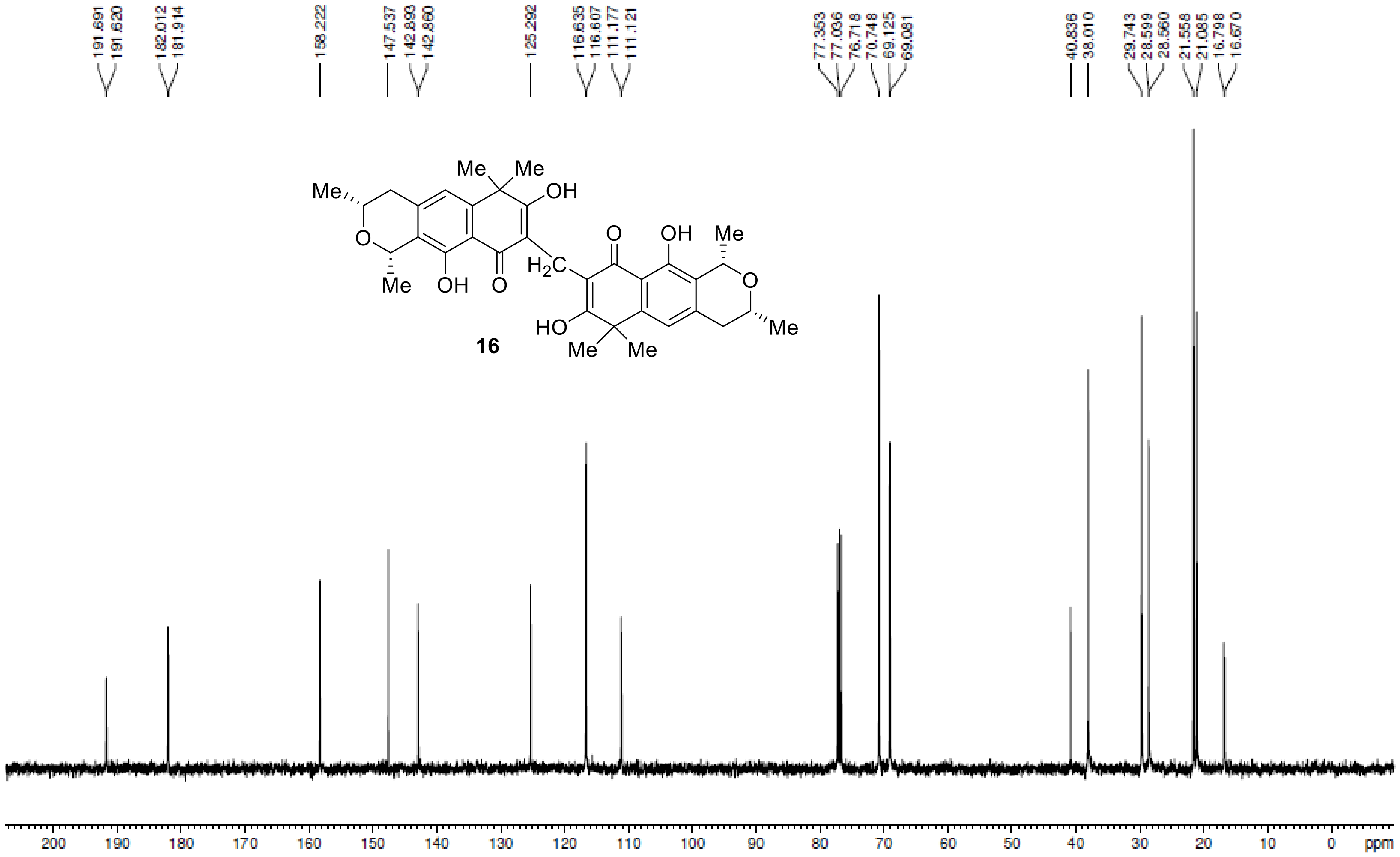

Figure S46 ^{1}H NMR (400 MHz) spectrum of compound **21** in acetone-d_6

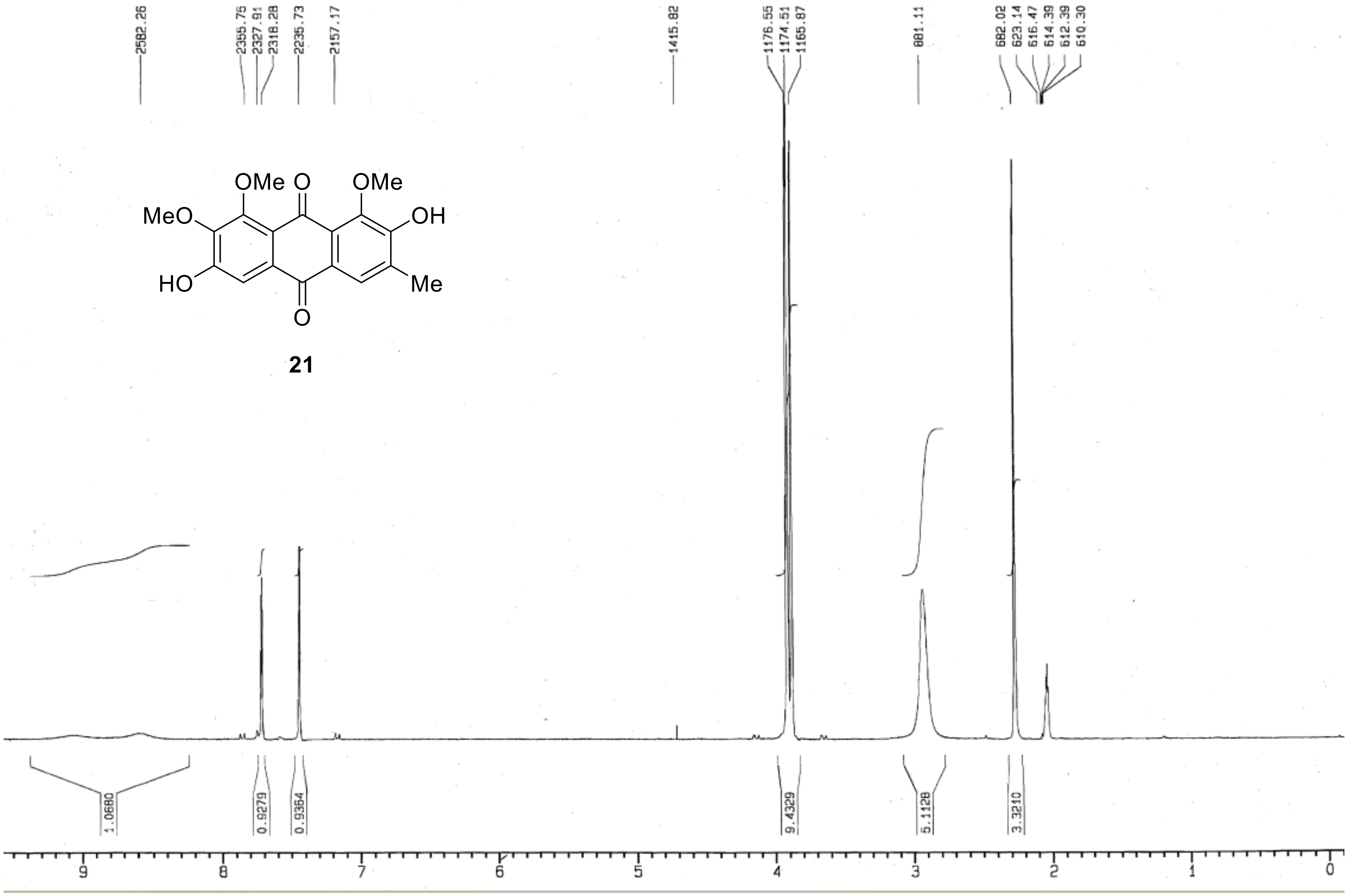

Figure S47 ^{13}C NMR (400 MHz) spectrum of compound **21** in acetone-d_6

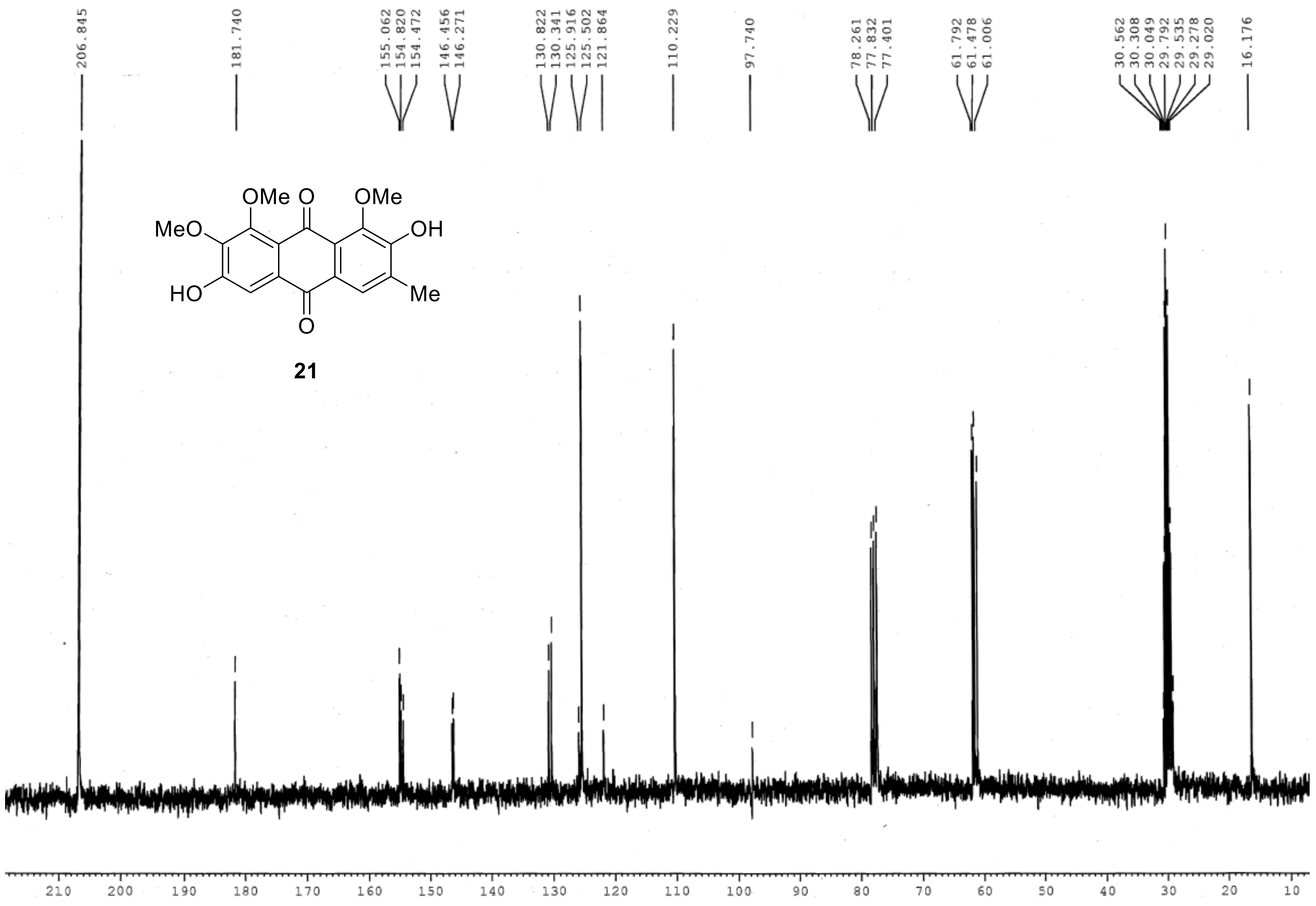

Figure S48 HPLC separation of (+)-**7** and (–)-**7**

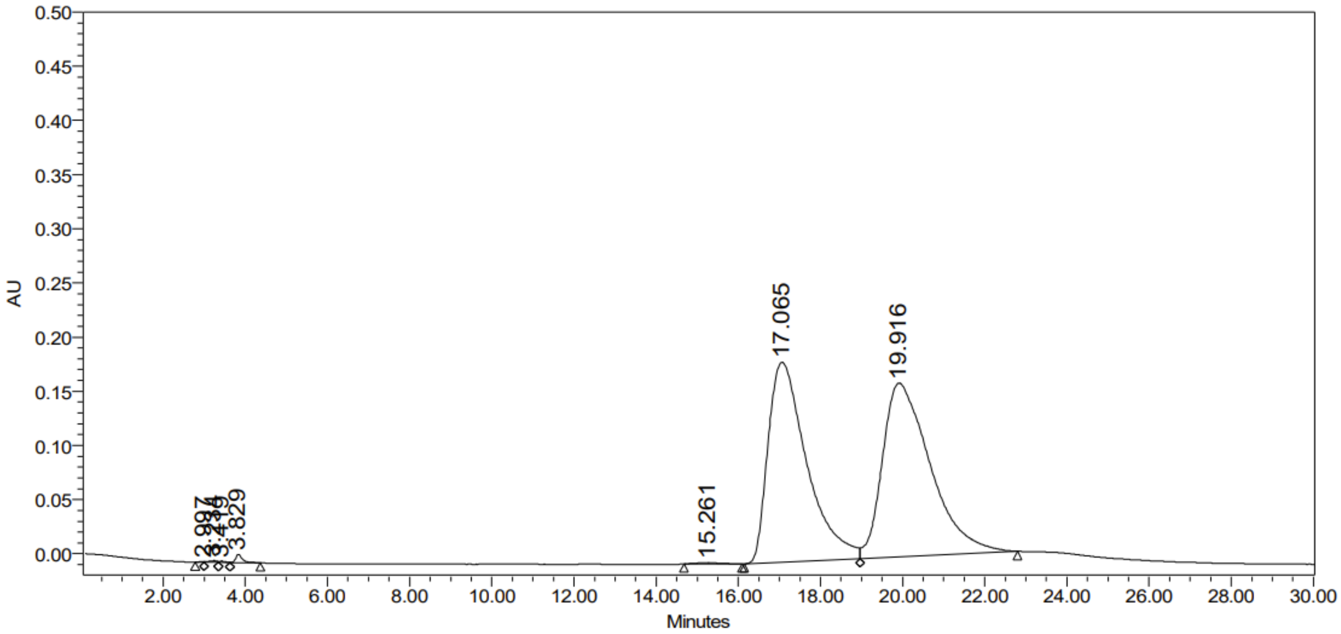

	RT	Area	% Area	Height
1	2.997	3548	0.01	445
2	3.234	20749	0.08	1544
3	3.419	13033	0.05	1138
4	3.829	85981	0.34	7659
5	15.261	51377	0.20	1135
6	17.065	12067024	47.85	184519
7	19.916	12976705	51.46	160385

HPLC information; Chiralpak OD-H column, IPA-hexane (5:95) as mobile phase, flow rate 1 mL/min

t_R = 17 min; (+)-**7** and t_R = 20 min; (–)-**7**

Figure S49 Overlayed ECD spectra of (+)-**7** and (–)-**7**

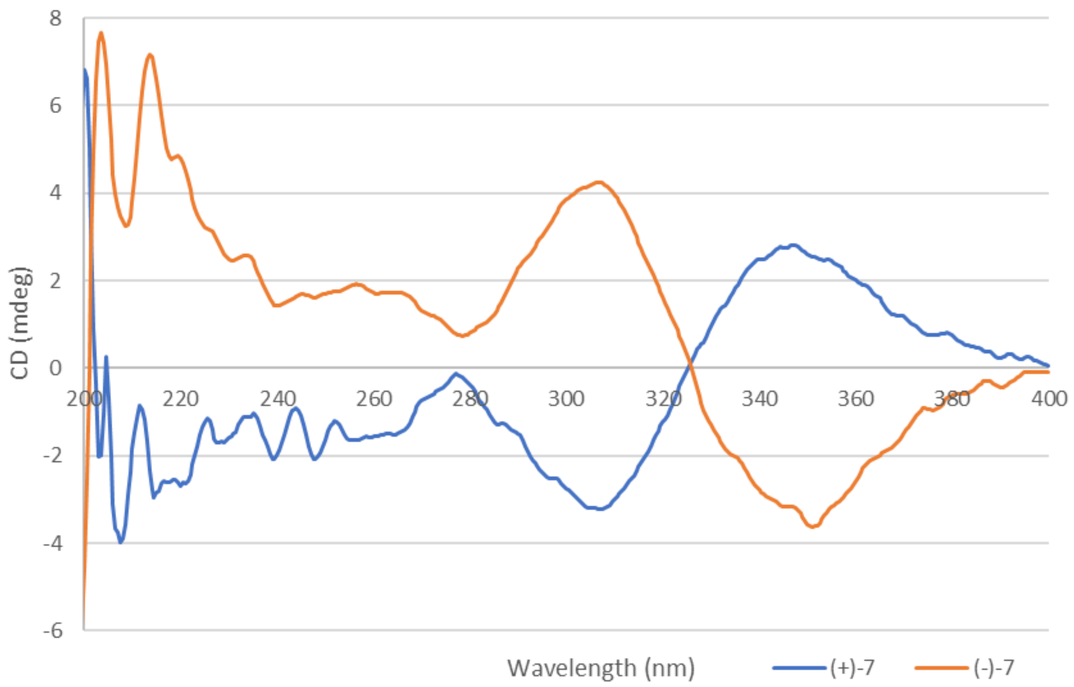

Figure S50 The comparison of optical rotation of **7** with related compounds

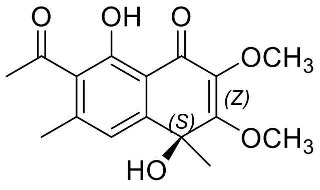

compound 7
−15.7 (c 0.12, $CHCl_3$)

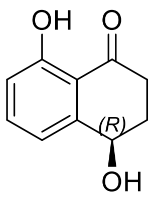

(−)-4*R*-regiolone[1]
−26.0 (c 0.03, $CHCl_3$)

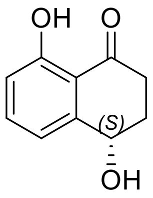

(+)-4*S*-isosclerone[1]
+24.5 (c 0.14, $CHCl_3$)

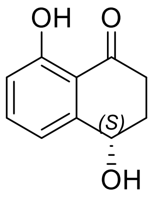

(+)-4*S*-isosclerone[2]
+21.7 (calculated)

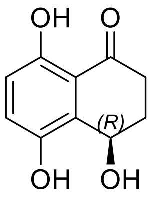

(−)-botrytone[3]
−20.0 (c 0.15, $CHCl_3$)

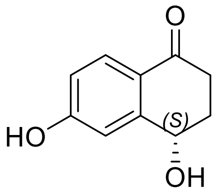

(4*S*)-4,6-dihydroxy-a -tetralone[4]
+19.0 (c 0.1, $CHCl_3$)

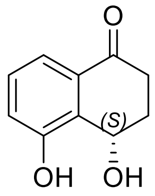

(4*S*)-4,5-dihydroxy-a -tetralone[4]
+35.0 (c 0.2, $CHCl_3$)

References

1. Machida, K.; Matsuoka, E.; Kasahara, T.; Kikuch, M.; Studies on the constituents of *Juglans species*. I. Structural determination of (4*S*)- and (4*R*)-4-hydroxy-α-tetralone derivatives from the fruit of *Juglans mandshurica* Maxim. var. sieboldiana Makino. *Chem Pharm Bull.* **2005**, *53*, 934–937.
2. Evidente, A.; Superchi, S.; Cimmino, A.; Mazzeo, G.; Mugnai, L.; Rubiales, D.; Andolfi. A.; Villegas-Fernández MA. Regiolone and Isosclerone, two enantiomeric phytotoxic naphthalenone pentaketides: computational assignment of absolute configuration and its relationship with phytotoxic activity. *Eur J Org Chem.* **2011**, *28*, 5564–5570.
3. Cimmino, A.; Villegas-Fernandez, AM.; Andolfi, A.; Melck, D.; Rubiales, D.; Evidente, A.; Botrytone, a new naphthalenone pentaketide produced by *Botrytis fabae*, the causal agent of chocolate spot disease on *Vicia faba*. *J Agric Food Chem.* **2011**, *59*, 9201–9206.
4. Liu, L.; Li, W.; Koike, K.; Zhang, S.; Nikaido, T.; New α-Tetralonyl Glucosides from the Fruit of *Juglans mandshurica*. *Chem Pharm Bull.* **2004**, *52*, 566–569.
